# Establishing a case definition of thiamine responsive disorders among infants and young children in Lao PDR: protocol for a prospective cohort study

**DOI:** 10.1136/bmjopen-2019-036539

**Published:** 2020-02-13

**Authors:** Sonja Y Hess, Taryn J Smith, Philip R Fischer, Indi Trehan, Laurent Hiffler, Charles D Arnold, Dalaphone Sitthideth, Daniel J Tancredi, Michael A Schick, Jay Yeh, Rebecca Stein-Wexler, Christine N McBeth, Xiuping Tan, Kouyang Nhiacha, Sengchanh Kounnavong

**Affiliations:** 1 Department of Nutrition, Institute for Global Nutrition, University of California Davis, Davis, California, USA; 2 Pediatric and Adolescent Medicine, Mayo, Rochester, Minnesota, USA; 3 Department of Pediatrics and Department of Global Health, University of Washington, Seattle, Washington, USA; 4 Lao Friends Hospital for Children, Luang Prabang, Lao People’s Democratic Republic; 5 Independent Pediatrician, Lagny sur Marne, France; 6 Lao Tropical and Public Health Institute, Vientiane, Lao People’s Democratic Republic; 7 Department of Pediatrics, University of California Davis Health System, Sacramento, California, USA; 8 Emergency Medicine, University of California Davis Health System, Sacramento, California, USA; 9 Department of Pediatrics, Division of Cardiology, University of California Davis Health System, Sacramento, California, USA; 10 Department of Radiology, University of California Davis Health System, Sacramento, California, USA; 11 Lao-Korea Children Hospital, Vientiane, Lao People’s Democratic Republic

**Keywords:** nutrition & dietetics, echocardiography, neuroradiology, paediatrics

## Abstract

**Introduction:**

Diagnosis of infantile thiamine deficiency disorders (TDD) is challenging due to the non-specific, highly variable clinical presentation, often leading to misdiagnosis. Our primary objective is to develop a case definition for thiamine responsive disorders (TRD) to determine among hospitalised infants and young children, which clinical features and risk factors identify those who respond positively to thiamine administration.

**Methods and analysis:**

This prospective study will enrol 662 children (aged 21 days to <18 months) seeking treatment for TDD symptoms. Children will be treated with intravenous or intramuscular thiamine (100 mg daily for a minimum of 3 days) alongside other interventions deemed appropriate. Baseline assessments, prior to thiamine administration, include a physical examination, echocardiogram and venous blood draw for the determination of thiamine biomarkers. Follow-up assessments include physical examinations (after 4, 8, 12, 24, 36, 48 and 72 hours), echocardiogram (after 24 and 48 hours) and one cranial ultrasound. During the hospital stay, maternal blood and breast-milk samples and diet, health, anthropometric and socio-demographic information will be collected for mother–child pairs. Using these data, a panel of expert paediatricians will determine TRD status for use as the dependent variable in logistic regression models. Models identifying predictors of TRD will be developed and validated for various scenarios. Clinical prediction model performance will be quantified by empirical area under the receiver operating characteristic curve, using resampling cross validation. A frequency-matched community-based cohort of mother–child pairs (n=265) will serve as comparison group for evaluation of potential risk factors for TRD.

**Ethics and dissemination:**

Ethical approval has been obtained from The National Ethics Committee for Health Research, Ministry of Health, Lao PDR and the Institutional Review Board of the University of California Davis. The results will be disseminated via scientific articles, presentations and workshops with representatives of the Ministry of Health.

**Trial registration number:**

NCT03626337.

**Strengths and limitations of this study**

- A standardised case definition for thiamine responsive disorder (TRD) will be proposed, through understanding of the interplay between clinical presentation, response to therapeutic thiamine administration and biochemical analyses, which will help more accurately diagnose TRD among infants and young children.
- The broad spectrum of eligibility criteria will ensure inclusion of hospitalised infants and young children who may have milder, underlying thiamine deficiency and may respond clinically and benefit from thiamine administration, as well as those with suspected beriberi.
- Associations between TRD and biomarkers of thiamine status will help establish deficiency cut-off thresholds for thiamine diphosphate and erythrocyte transketolase activity coefficient that are relevant to a population with thiamine deficiency.
- Due to ethical considerations, this study does not include a placebo control group and will rely on a community comparison group to cross-check risk factors identified in the hospitalised cohort to a healthier population.
- In addition to the thiamine administration, hospitalised children will continue to receive routine care, such as antibiotics and other medications for concurrent illnesses that may affect the children’s recovery, making the clear distinction between TRD cases and non-responders more challenging.

## Introduction

Thiamine (vitamin B_1_) is an essential micronutrient, required as a cofactor for several enzymes involved in carbohydrate metabolism and energy production. Thiamine deficiency has been described in several low-income and middle-income countries and remains a public health concern in many countries of Southeast Asia,^1 2^ including Lao People’s Democratic Republic (Lao PDR).^3–5^ Diets that lack in dietary diversity and rely too heavily on low-thiamine staple crops (such as polished white rice), food preparation practices (repetitive washing and soaking rice for many hours) and traditional postpartum restrictive diets, all place lactating women at high risk of thiamine deficiency in Lao PDR.^6–8^ Breast-milk thiamine concentrations and infant thiamine status are strongly dependent on maternal thiamine intake and status, and maternal thiamine deficiency rapidly results in low breast-milk thiamine concentrations.^9^ Infants are therefore especially vulnerable to thiamine deficiency in the first months of life, and exclusively breastfed infants of thiamine-deficient mothers are at the highest risk. According to the WHO, infantile thiamine deficiency is the only serious form of malnutrition that occurs in adequately breastfed infants and only when the mother herself is thiamine-deficient.^10^

Thiamine deficiency presents as a broad spectrum of non-specific clinical abnormalities, referred to as thiamine deficiency disorders (TDD), of which the classic forms of beriberi in adults and children are best known, although even classic beriberi often goes unrecognised.^11^ TDD can be characterised to some degree by age and features of clinical presentation.^2^ Infantile TDD commonly occurs between 1 and 9 months of age, with non-specific early signs such as refusal to breast feed, vomiting and irritability (1–3 months of age), which can progress to oedema, respiratory distress, heart failure and death if not rapidly treated. During later infancy, the cry may sound hoarse or change to a soundless cry (4–6 months of age) and neurological symptoms may present, including nystagmus, convulsions and unconsciousness (7–9 months of age). Infants with encephalitic beriberi were found to have abnormal cranial ultrasonography, with normalisation within 8 weeks of thiamine administration.^12^ In the second year of life and later in childhood, TDD more typically presents with a broad range of neurological symptoms, such as anorexia, irritability, agitation, muscle pain, reduced deep tendon reflexes, ataxia, paralysis and altered levels of consciousness.^11 13–15^ Due to uncertainties regarding the diagnostic criteria and overlapping symptoms with other conditions, TDD may frequently be misdiagnosed as bronchiolitis, pneumonia, typhoid fever, sepsis, meningitis or encephalitis, and the underlying thiamine deficiency may remain untreated.^1 2 11^ Cases of TDD are often suspected rather than confirmed, and a final diagnosis is usually based on a positive response to intramuscular or intravenous thiamine administration.

Diagnosis of TDD is further complicated by the fact that several studies have found little association between biomarkers of thiamine status and TDD-like symptoms.^16–18^ It has been suggested that low thiamine status alone cannot reliably discriminate between symptomatic and asymptomatic infants, and infectious co-pathologies and other physiological stressors may precipitate the onset of TDD.^17^ Conversely, low thiamine status appears to increase vulnerability to disease, as indicated by the observation that hospitalised infants with low thiamine status, but no clinical signs of beriberi, showed higher mortality rates in a region of Lao PDR.^4^

Thiamine status can be determined by assessing thiamine metabolites in blood or urine. The most widely used biochemical indicator of thiamine status is the activity of the thiamine-dependent transketolase enzyme in erythrocytes (ETK), which reflects the biological functionality of the vitamin and is sensitive to marginal thiamine deficiency.^19^ Thiamine diphosphate (ThDP) concentration in whole blood or erythrocytes is also a useful indicator as ThDP is the biologically active vitamer and is reflective of body stores. There are however many challenges to assessing biomarkers of thiamine status, especially in resource-limited settings, including cost, the required cold chain and limited availability of laboratories able to analyse biological specimens. Hence, thiamine biomarkers are not routinely assessed and a rapid positive response to therapeutic thiamine is considered confirmation of a deficient status. Furthermore, there are currently no universally agreed on adequacy or deficiency cut-off thresholds for erythrocyte or whole blood ThDP or ETK activity coefficient (ETKac), which have largely been studied in thiamine adequate adults in high-income countries. Consequently, a wide range of cut-offs has been used.^1^ Limited data derived from two studies in European adults at risk of thiamine deficiency from either chronic alcoholism or from an acute or chronic medical condition are available on associations between ETKac and ThDP.^20 21^ These studies suggest that elevated ETKac (≥25%) occurs when ThDP concentrations are below 280 mg/g haemoglobin. However, it is not clear if similar results would be apparent in other populations. Thus, there is a need to develop clinically meaningful cut-off thresholds for biomarkers indicative of thiamine deficiency in other at-risk populations, especially infants and young children in countries where thiamine intake is inadequate and exclusive breast feeding is prevalent.

In summary, owing to the wide variability in clinical presentation, a barrier for reliable diagnosis and targeted treatment of TDD is the lack of a clear clinical case definition with broad consensus. There is a need to understand the interaction between thiamine status, exposure to risk factors (including infections) and onset of clinical symptoms in areas where thiamine deficiency is prevalent. Thus, this study aims to develop a standardised case definition for thiamine responsive disorders (TRD), based on a combination of presenting clinical features most predictive of responding to therapeutic thiamine doses, alongside assessment of biochemical indicators of thiamine status and potential risk factors, in infants and young children aged 21 days to <18 months in Lao PDR.

## Objectives of study

The primary objective of this study is the development of a case definition for TRD among infants and young children with symptoms consistent with TDD, with the case definition based on those clinical symptoms and other predictors that we find are better able to distinguish those who respond positively to thiamine administration from those who do not respond. As diagnostic tools available to treating physicians may differ by setting, we will repeat analyses after excluding from the set of candidate predictors those that are derived from assessments only available in higher-resource settings, such as laboratory biomarkers and ultrasonography.

A secondary objective is to fill the knowledge gap surrounding biomarkers of thiamine status in at-risk populations. Biomarker cut-offs for TRD will be developed in the hospital cohort and distributions will be characterised in both the hospital and community cohorts. The performance of our proposed cut-off will be compared with the performance of existing literature cut-offs.

Additionally, all identified predictors and biomarker cut-offs will be compared across the community cohort, non-TRD hospital cohort and TRD hospital cohort to assess the prevalence of risk factors among apparently healthy infants and young children and assess the usefulness of the TRD case definition in various settings.

## Methods and analysis

### Study design and procedures

#### Study design

**Figure 1:**
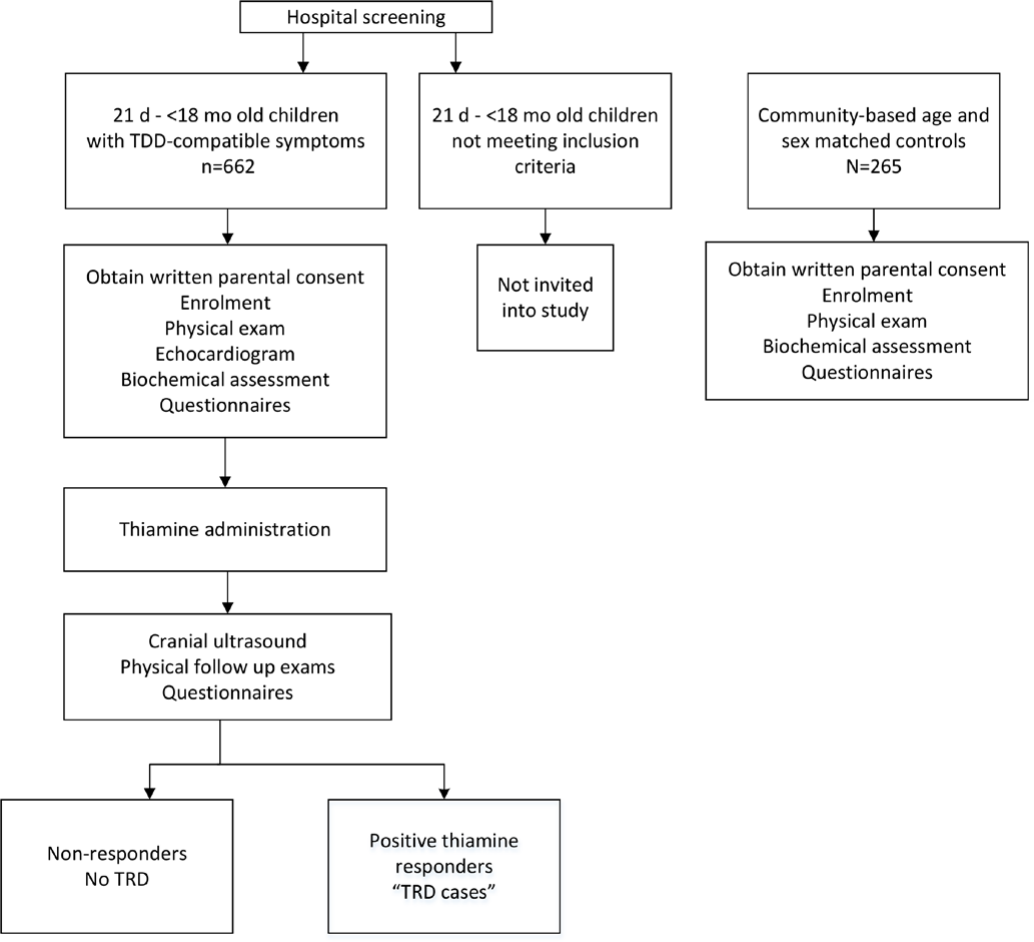
Study design overview of hospitalised and community cohort in the Lao Thiamine Study. At the onset of the Lao Thiamine Study, the eligibility age was 21 days to <12 months. The target age was expanded to <18 months after 18.1% of hospital infants had been enrolled, due to much lower enrolment rates than anticipated, and after consultation with expert paediatricians and the hospital leadership.

The study uses a prospective study design and includes a cohort of infants and young children 21 days to <18 months of age who are identified as having TDD-compatible symptoms (figure 1). Children will be recruited at the Lao Friends Hospital for Children (LFHC) in Luang Prabang, Lao PDR, and potentially eligible children presenting with any of the below described inclusion criteria will be identified through close collaboration between the LFHC hospital staff and the study team. Enrolled children will be treated with thiamine and all other clinically indicated treatments and followed closely over 48–72 hours, as described in more detail below. Based on the response to thiamine, children will either be defined as TRD cases or non-responders.

A community-based cohort of children frequency matched by age, sex and village of residence will be included in this study to serve as a comparison group for evaluation of potential predictors of TRD and thiamine deficiency in a non-hospitalised cohort. We will aim to achieve a comparison group that has a similar distribution of age, sex and location of residence as the hospitalised children to minimise confounding biases. To achieve a well-balanced match, we will summarise the characteristics of the hospitalised children and enrol frequency-matched community-based children weekly to avoid seasonal discordance. Prior to visiting the health district, the head of the health centre and the head of the village will be contacted to ask for assistance in identifying a child of the right target age (within a 2-week period) and living in a selected village or, if not available, a neighbouring village. The health centre staff will establish contact with the child’s parents/caregivers, briefly explain the study and invite the parents/caregivers for a study appointment at the health centre. All mothers of participating children in the hospital and community will also be invited into the study to obtain in-depth information on potential risk factors, including maternal thiamine status and thiamine concentration in breastmilk.

#### Inclusion and exclusion criteria

##### Inclusion and exclusion criteria of children at the hospital

Initially, infants 21 days to <12 months of age were the target population for the present study. This target age range was chosen because the risk of beriberi is highest during infancy, with highest mortality rates between 2 and 4 months of age.^10^ Very young infants from 0 to 20 days of age are not eligible, because beriberi is rarely reported in the first few weeks of life and we did not want to include very young infants at greater risk of other serious illnesses, such as newborn sepsis, which could confound the study findings. The target age range was expanded to <18 months of age after 18.1% of the target hospital sample size had been enrolled. This extension of eligibility criteria was implemented due to much lower enrolment rates than anticipated in the first few months of the study, after further review of the scientific literature and in consultation with expert paediatricians and the hospital leadership. Thus, throughout the majority of the study duration, the target age is 21 days to <18 months.

Children in the target age range, who are admitted to inpatient care at LFHC, will be screened by a study physician and/or study nurse to determine the presence of at least one of the following inclusion criteria:

- Liver enlargement (>2 cm below right costal margin, supine examination).
- Oedema.
- Tachypnoea (respiratory rate >60/min for 3–8 weeks; >50/min for 2–11 months; >40/min for 12–18 months).
- Tachycardia (heart rate >160/min for <12 months; >120/min for 12–18 months).
- Oxygen saturation <92%.
- Difficulty breathing (chest in-drawing, or nasal flaring).
- Refusal to breast feed or refusal of infant formula or food for greater than 24 hours.
- Repetitive or recurrent vomiting with no obvious other cause (ie, vomiting >3 times in past 24 hours)
- Persistent crying not relieved by soothing or feeding with no obvious other cause.
- Hoarse voice/cry or loss of voice.
- Nystagmus or other unusual eye movements.
- Muscle twitching.
- Loss of consciousness.
- Convulsion.
- Opisthotonus/abnormal posturing.
- Acute paralysis/flaccid paralysis.

The list of inclusion criteria has been developed based on TDD-compatible signs and symptoms in the broadest sense, in an effort to reduce the risk of potentially omitting children who would respond clinically to thiamine administration to correct any deficiencies. Tachycardia and two other inclusion criteria (opisthotonus/abnormal posturing and acute paralysis/flaccid paralysis) were added once the target age range was extended to <18 months of age to capture the full range of neurological signs and symptoms that might occur in the second year of life.

##### Inclusion and exclusion criteria of children in the community

The eligibility age for the community children is identical to that of the hospital. Thus, initially the target age range was 21 days to <12 months, and this was expanded to 21 days to <18 months after 13.6% of the community sample size had been enrolled. Other inclusion criteria for the community cohort are that community children match the characteristics of the hospitalised children with regard to age, sex and community of residence characteristics. Children are not excluded based on any ineligibility criteria, except in case of severe acute illness warranting immediate hospital referral.

##### Inclusion and exclusion criteria of study participants’ mothers

Once the hospitalised and community-based children are enrolled, their mothers will also be invited for study participation, unless the women meet one of the following exclusion criteria: (1) severe acute illness warranting immediate hospital referral or (2) unable to provide informed consent due to reduced decision-making ability.

#### Informed consent

Informed consent will be obtained from at least one primary caregiver (parent or legal guardian) of children for the child’s study participation as well as the mothers (or other female primary caregiver) for their own study participation. Since the study activities vary depending on the study participant (child vs mother) and the location (hospital vs community), different consent forms will be used. If the parents and/or mothers cannot read, an impartial witness will be present during the entire consent discussion to attest that the information in the consent form was accurately explained. If the parents do not understand Lao, but speak Hmong, a Hmong-speaking study team member will lead the consenting procedure. In case of other languages, an individual will be identified to help translate all information into the appropriate language. The consent form will be read with the parents/mothers and all information will be provided ensuring that the parents/mothers understand what it will be like for their child or the mothers, respectively, to participate in the study, and that the choice to participate will not affect the medical care that the child will receive while in the hospital or at any health centre. If the primary caregivers decide for the child to participate, one parent, either the mother, father or a legal guardian, or both, will be asked to sign or fingerprint the consent form of the child. The mother will be asked to sign or fingerprint her own consent form to confirm voluntary study participation. An additional signature or fingerprint will be asked to confirm voluntary consent to storage and future analyses of biological specimen.

Once a child is identified as potentially eligible during the initial physical examination by the hospital medical team, a brief summary of the research study will be provided to explore the interest in study participation. In case the parent expresses an initial interest, and the child’s health is stable, a study team member will give a copy of the approved consent form translated into Lao to the parent and the above described consenting procedure will be implemented. The same consenting procedure will be followed, but delayed, if the child’s condition is critical or life threatening. In case the child’s health does not allow postponing any examination or treatment, the child will be examined by a study physician and/or nurse and all health information will be recorded. A venous blood sample will be drawn and treatments (including intravenous or intramuscular thiamine) will be initiated prior to obtaining parental consent. Parental consent will be obtained as soon as the child’s condition has stabilised, following the above described procedures. Any data and blood specimen that were collected prior to consent will only be used if parental written consent is obtained.

#### Intervention

Children in the hospital cohort will be treated by LFHC staff following standard care practices at the discretion of the primary medical team. The only intervention provided in the present study is thiamine. There are currently no evidence-based recommendations for thiamine dosage to treat infants and young children with severe acute illness.^11^ Treatment guidelines in the pocket book of the WHO Western Pacific Region recommend treating an infant with heart failure with 25 mg thiamine intravenously and 25 mg intramuscularly, then 25 mg thiamine daily until the infant can eat, followed by 10 mg oral thiamine supplementation per day for 2–3 weeks.^22^ Considering that previous studies used dosages ranging from 50 to 1500 mg depending on the clinical condition,^17 23–26^ and neurological presentations of TDD tend to require higher doses and longer recovery time,^11^ a daily dose of 25–50 mg was considered potentially insufficient. Consequently, daily doses of 100 mg thiamine, the standard treatment procedure of infants presenting with TDD at the LFHC, was deemed most appropriate for the present study. All children in the hospital cohort will be treated with a daily dose of 100 mg thiamine by intramuscular or intravenous administration for a minimum of 3 days. In general, intravenous dosing will be preferred, unless intravenous access cannot be obtained after several attempts by LFHC nurses. Vials containing 100 mg thiamine as thiamine hydrochloride will be obtained locally (CBF Pharmaceutical Factory, Ban Kang, Pakse District, Lao PDR).

#### Procedures

##### Procedures at the hospital

Once the LFHC medical team have assessed the health of a potentially eligible patient, the study physician and/or nurse will complete the first standardised physical examination (ie, baseline health status) by following a structured data collection form. The physical examination includes questions for caregivers on the child’s recent health history, assessment of the child’s general appearance, presence of cyanosis, breathing difficulties, bulging fontanelle, hypotonia, unusual eye movements, ptosis, sound of the child’s voice/cry, muscle twitching, level of consciousness (assessed by the ‘alert, response to voice, response to pain, unresponsive’ scale^22^), liver palpation and oedema. Vital signs such as body temperature (Thermo Buddy Infrared Ear Thermometer TB-100, HuBDIC Co., Anyang-si, Gyeonggi-do, Korea) and oxygen saturation of arterial haemoglobin, pulse rate, perfusion index and respiration rate from pulse oximeter plethysmogram analysis will be measured using a Rad-G Pulse Oximeter (Masimo, Irvine, California, USA) (online supplementary table 1). A venous blood sample will be drawn and an echocardiogram will be performed, as described in more detail below. These data collection activities are performed prior to the first thiamine administration, unless the child’s condition is critical, in which case the echocardiogram may be delayed until after the LFHC medical staff have initiated the thiamine administration and any appropriate treatments and the child’s health is more stable. The date and time of each activity will be recorded for later consideration during statistical analyses. A cranial ultrasound will be performed during the hospital stay, typically within the first 12 hours.

10.1136/bmjopen-2019-036539.supp1 Supplementary data

**Figure 2:**
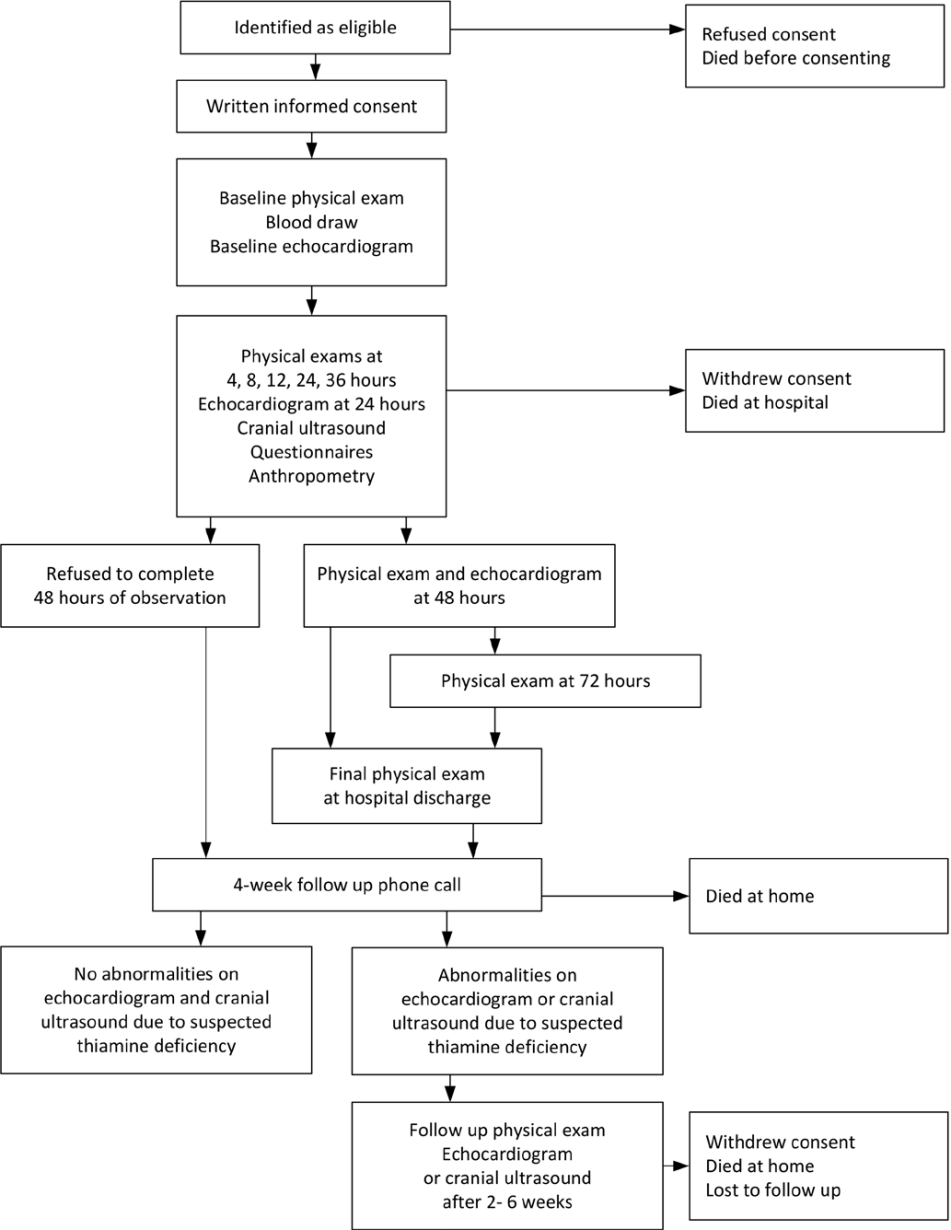
Flow chart of data collection timing among hospitalised infants and young children in the Lao Thiamine Study.

The data collection will follow a structured timeline after the first thiamine dose has been administered (table 1). In particular, the thiamine administration will be defined as hour zero, and the above described physical examination will be repeated 4, 8, 12, 24, 36, 48 and 72 hours after the initial thiamine administration (figure 2). At each follow-up time point, recovery status will also be assessed. Namely, at 4, 8, 12, 24, 36, 48 and 72 hours the child’s health status will be described by the study physicians/nurses using a five-point scale (seems worse; no change; seems a little bit better; seems a lot better; seems recovered). In addition, during the follow-up examination after 24, 36, 48 and 72 hours, the study physicians will consult with the LFHC treating physicians whether the child is well enough to be discharged from the hospital. If a child’s health has stabilised before 48 hours, the parents will be asked if they are willing to remain at the hospital until 48 hours. If a child’s health has stabilised by 48 hours and is ready to be discharged, direct observation will end after 48 hours. Otherwise data collection will continue until 72 hours. If a patient is unstable after 72 hours, he/she will remain at the hospital for further consultation by the hospital, but no further data will be collected until a final physical examination at discharge. Children who show abnormalities in the ultrasound scans that may be due to suspected thiamine deficiency and those with neurological symptoms at the time of discharge will be invited for a follow-up physical examination after 2–6 weeks.

**Table 1:** Timing of study procedures of hospitalised study children and their mothers

Activity	Prior to enrolment	Baseline	Medical treatment	Hours and weeks after thiamine administration										
				0–1 hour	4 hours	8 hours	12 hours	24 hours	36 hours	48 hours	72 hours	>72 hours*	2–4 weeks†	6 weeks‡
Child’s study participation and data collection														
Care seeking at hospital	X													
Parental consent for child’s participation§	X													
Enrolment		X												
Venous blood sample		X												
Thiamine administration			X					X		X				
Other clinically indicated treatments			XXXXXXXXXXXXXXXXXXXXXXXXXXXXXXXXXXXXXXXXXXXXXXXXXXXXXXXXXXXXXXXXXXXXXXX											
General physical examination		X			X	X	X	X	X	X	X	X	X	X
Liver palpation		X			X	X	X	X	X	X	X	X	X	X
Heart rate		X			X	X	X	X	X	X	X	X	X	X
Examination for oedema		X			X	X	X	X	X	X	X	X	X	X
Respiratory rate, oxygen saturation		X			X	X	X	X	X	X	X	X	X	X
Eye movement/contact assessment		X			X	X	X	X	X	X	X	X	X	X
Signs of ptosis and ophthalmoplegia		X			X	X	X	X	X	X	X	X	X	X
Hypotonia (head lag, floppy arms or legs in face-down position)		X			X	X	X	X	X	X	X	X	X	X
Consciousness evaluation		X			X	X	X	X	X	X	X	X	X	X
Vomiting frequency		X			X	X	X	X	X	X	X	X	X	X
Feeding behaviour		X			X	X	X	X	X	X	X	X	X	X
Anthropometry								X						
Echocardiogram		X						X		X			X	
Cranial ultrasound				X										X
Potential adverse events from thiamine injection			X	X	X	X	X	X	X	X	X	X		
Questionnaires														
Basic child characteristics		X												
Child’s birth and health history¶					X									
Socio-economic characteristics and food security of child’s family¶					X									
Dietary practices of child¶					X									
Mother’s study participation and data collection														
Maternal consent	X**													
Maternal health¶					X									
Maternal dietary practices¶					X									
Maternal blood††					X									
Breast milk††					X									
Maternal anthropometry††										X				

*Final physical examination prior to hospital discharge; timing will depend on hospital duration and time of discharge.

†Children with abnormal echocardiograms due to suspected thiamine deficiency will be invited for a follow-up physical examination and echocardiogram after 2–4 weeks.

‡Children with abnormal cranial ultrasounds or neurological symptoms due to suspected thiamine deficiency will be invited for a follow-up physical examination and cranial ultrasound after 6 weeks.

§In case of life-threatening condition, the child will be examined, blood will be drawn and all required medical interventions will be provided (including thiamine administration) prior to obtaining parental consent. Parental consent will be obtained as soon as the child’s condition has stabilised. Any data that have been collected will only be used, if parental written consent is obtained.

¶Questionnaires will be administered during the hospital stay of the child. Timing will depend on hospital procedures and physical examinations.

**Maternal consent will be obtained prior to enrolment into the study. The timing of obtaining maternal consent will depend on the health status of her child and hospital procedures.

††Maternal blood and breast milk will be collected as soon as possible after the mother’s written consent is obtained. Maternal anthropometry will be completed at a convenient time before the child’s discharge.

##### Procedures in the community

After obtaining written consent for the child’s and mother’s participation, a study nurse will perform a brief physical examination of the child, assessing the same vital signs as for hospitalised children (online supplementary table 1). A venous blood sample will be collected from both child and mother for immediate processing in a mobile laboratory; anthropometric measurements will be conducted; breast milk will be collected and questionnaires will be completed.

Children will be referred to the nearest health centre in the case of severe acute malnutrition (weight-for-length z score <−3 SD^27^) and acute illnesses. In case of haemoglobin concentrations <100 g/L in both children and their mothers, the parent of the anaemic child and/or the anaemic mother will be informed by phone and referred to the nearest health centre for moderate or severe anaemia.

#### Blood sample collection

Venous blood will be collected from children and their mothers by trained nurses or laboratory technicians. Prior to the first thiamine administration, 5 mL venous blood will be collected from children into 6 mL BD vacutainer tubes coated with K_2_ EDTA (ref. 367863, Becton, Dickinson & Company, Franklin Lakes, New Jersey, USA). From the children’s mothers, 10 mL venous blood samples will be collected into 10 mL BD vacutainer tubes coated with K_2_ EDTA (ref. 368589, Becton, Dickinson & Company). Blood samples will be processed immediately after they are collected, either at the hospital laboratory or in a mobile field laboratory station that will be established in each community. In particular, efforts will be made to analyse the blood for lactate concentration within 30 min and to wash the red blood cells within 2 hours of the blood draw, as described in more detail below.

Fresh venous blood will be used for determination of the lactate concentration by StatStrip Lactate (Nova Biomedical, Waltham, Massachusetts, USA) among hospitalised children, and analysis of the complete blood count (CBC) by haematology analyser (BC-3000 Plus Auto Hematology Analyzer, Shenzhen Mindray Bio-Medical Electronics Co., Nanshan, Shenzhen, China). In the community, a small whole blood aliquot will be stored at 4°C–10°C and analysed for CBC on arrival at the hospital laboratory, and within 8 hours of blood collection. For later assessment of ThDP, whole blood will be aliquoted into amber microcentrifuge tubes and stored at −80°C. From a convenience sample of mothers (n~200) with a complete 10 mL blood sample, 75 µL of blood will be pipetted into each of five circles of a dried blood spot card (Whatman 903 Protein Saver).

The remaining blood will be centrifuged at 3200 rpm for 10 min (DM0412S, DLAB Scientific Co., Beijing, China). This speed corresponds to 1202 × g for the children’s and to 1278 × g for the mothers’ vacutainer tubes, respectively. Plasma will be aliquoted into microcentrifuge tubes of various volumes (0.2 to 1.5 mL) and types (amber and clear). After transferring the entire buffy coat carefully into a 1.5 mL microcentrifuge tube, packed erythrocytes will be washed three times in physiological saline (0.9% NaCl) and centrifuged at 4000 rpm for 10 min each time, which corresponds to 1878 × g and 2057 × g for children’s and mother’s tubes, respectively. All samples aliquoted in the community for later assessment of thiamine and other nutritional and health indicators will be frozen in an electronic cool box at −20°C and transferred to the −80°C freezer on arrival at the hospital.

#### Blood biomarker analysis

The concentration of ThDP will be measured using the well-established thiochrome reaction coupled with high-performance liquid-chromatography fluorescence detection method.^28 29^ The assay of ETK will be completed by UV spectrophotometer at the NIHR BRC Nutritional Biomarker Laboratory at the University of Cambridge in the UK. The activity of transketolase is measured in several steps: (1) the basal activity of transketolase is measured in erythrocytes (basal ETK); (2) the ETK activity is then determined in vitro by adding exogenous ThDP in excess (ETK activity) and (3) the ETKac is then calculated by dividing the ETK activity by the basal ETK. C-reactive protein and alpha-1-acid-glycoprotein will be analysed by the the enzyme-linked immunosorbent assay (ELISA).

#### Breast-milk sample collection and biomarker analysis

Breast-milk collection will be attempted from all lactating mothers. Since mothers of children with suspected beriberi are routinely prescribed oral thiamine supplementation at LFHC (100 mg twice daily for 30 days as per WHO recommendations^22^), breast milk and maternal blood will be collected prior to any supplement intake.

Prior to breast-milk collection, the breast will be cleaned with a sanitary wipe (Hygea Obstetrical Towelette, PDI Healthcare, Orangeburg, New York, USA). Breast milk of the fuller breast will be collected into a breast-milk bag with an electronic hospital-grade breast pump (Symphony Pump, Medela AG, Baar, Switzerland) until the breast is emptied. Since circadian differences in concentrations of thiamine and other vitamins are small,^28^ the breast-milk sample will be collected at any time of day. Time of day, time since last meal, breast side, time since last emptying of that side and milk volume will be recorded. Samples will be mixed well before aliquoting into 1.5 mL amber and clear microcentrifuge tubes and frozen at −80°C until analysis at the USDA Western Human Nutrition Research Center at UC Davis (USA). Samples will be analysed for thiamine concentration by ultra-performance liquid chromatography tandem mass spectrometry using internal pooled breast-milk samples for quality control.^30^

#### Specimen banking

Any remaining biochemical samples (whole blood, red blood cells, plasma, dried blood spots and breast milk) will be stored at UC Davis for possible future analyses of indicators of interest.

#### Ultrasound performance and interpretation

An echocardiogram and cranial ultrasound will be performed with a portable ultrasound machine (M9, Shenzhen Mindray Bio-Medical Electronics Co.) in all hospitalised children. For the echocardiogram, study physicians will capture 2 s video clips of the heart in apical four chamber, parasternal long-axis and parasternal short-axis views. Required still images for standard measurements will include left ventricular dimensions in diastole and systole by M-mode, E-point septal separation in the parasternal long axis and tricuspid annular plane systolic excursion.^31^ For the cranial ultrasound, study physicians will capture six standard coronal and five standard sagittal still images of the brain, with additional images of the putamina, caudate nuclei and thalamus. Cranial ultrasound will primarily be performed among infants. However, we will attempt cranial ultrasound among >12 months old children in case the anterior fontanelle has not yet closed entirely, which may allow ultrasonography of the basal ganglia. The echocardiogram will be completed at baseline (ie, prior to or soon after the first thiamine administration) and repeated at 24 and 48 hours. Since normalisation of encephalitic beriberi occurs slowly,^12^ the cranial ultrasound will be performed once during the hospital stay, typically within the first 12 hours.

All ultrasonography completed by study physicians or nurses will be uploaded to Box (Box, Redwood City, California, USA). All baseline echocardiograms and cranial ultrasounds will be reviewed by one of the two expert sonographers at UC Davis. If the baseline echocardiogram is flagged as abnormal by one of these reviewers, the 24 and 48 hours echocardiograms will also be reviewed by these same reviewers. In addition, all abnormal echocardiograms and cranial ultrasounds will be reviewed by a paediatric cardiologist and a paediatric radiologist at UC Davis, respectively, who will provide a description of the observed abnormality. Any abnormality identified by the paediatric cardiologist and/or paediatric radiologist will be communicated to the medical leadership of the LFHC to initiate appropriate action. In case of abnormalities suggestive of thiamine deficiency, the study children will be invited for a follow-up examination after 2–4 weeks in case of an abnormal echocardiogram and after 6 weeks in case of an abnormal cranial ultrasound, respectively.

For quality assurance, 10% of echocardiograms and cranial scans judged as normal will be randomly selected and re-read by the paediatric cardiologists and the paediatric radiologist at UC Davis, respectively. If an echocardiogram is judged as normal during the first review, the 24 and 48 hours echocardiograms will typically not be reviewed. However, 10% of randomly selected 24 and 48 hours echocardiograms will be re-read by the UC Davis expert sonographers to verify the description of the baseline echocardiography.

#### Anthropometric assessment

Anthropometry of both children and their mothers will be assessed in the hospital and the community following protocols proposed by the Food and Nutrition Technical Assistance III Project.^32^ Unclothed or lightly dressed children will be weighed to the nearest 20 g (SECA 383, Hamburg, Germany). Children’s recumbent length (SECA 417, Hamburg, Germany), left mid-upper am circumference (MUAC; Shorr Child MUAC Tape, Weigh and Measure, Olney, Maryland, USA) and head circumference (ShorrTape 65 cm Measuring Tape, Weigh and Measure) will be measured to the nearest 0.1 cm. All measurements will be collected in duplicate and the average of the two measurements will be calculated. If measurements differ by >0.1 kg (weight), or >0.5 cm (length, MUAC, head circumference), the measurement will be repeated a third time and the average of the two closest measurements will be calculated. Maternal weight (SECA 874, Hamburg, Germany), height (SECA 213, Hamburg, Germany) and left MUAC (ShorrTape 65 cm Measuring Tape) will be assessed using a similar approach. Regular standardisation sessions will be implemented to compare the performance of anthropometry teams among themselves and with their supervisors.^33 34^

#### Risk factor and predictor assessment

A broad range of information will be collected using structured questionnaires (online supplementary table 1). Data collection forms were developed in English, programmed in Survey CTO (Dobility, Cambridge, Massachusetts, USA) and translated into Lao for electronic data collection.

Specific questionnaires were developed to assess the child’s birth history, and the child and mother’s health history. Since traditional restrictive postpartum diets are very common in the study population, an extensive questionnaire was developed to record dietary patterns both during pregnancy and the postpartum period. Other questionnaires, such as the assessment of socio-economic status, and child and mother dietary practice questionnaires, were adapted from the Lao Zinc Study.^35^ Since women and children may change their dietary intake due to their presence at the hospital in Luang Prabang, we will ask the mother to report food intake for the days prior to their arrival at the hospital. Household food security will be assessed using the Household Food Insecurity Access Scale,^36^ and social desirability of respondents will be assessed as proposed by Crowne and Marlowe and Menon *et al*.^37 38^ Two brief questionnaires will be used to record the reason for exiting the study by children and mothers, respectively (ie, completion of data collection, withdrawal of consent, death or other reasons).

#### Patient and public involvement

Patients and the public were not involved in the design of this study.

### Case report review

To develop a clinician-guided diagnostic impression of TRD, three expert paediatricians specialised in tropical medicine will review the case reports of each hospitalised child to judge whether a child had TRD using a three-point scale (probable TRD (including classic beriberi); possible TRD and not likely TRD). Specifically, each case report will be reviewed by three expert paediatricians. When there is disagreement (defined as separate raters classifying the same case as probable TRD and not likely TRD) or when there is no agreement (ie, each rater classifies a case into a different category), efforts will be made to reach consensus between the three experts. A case report template was developed for this purpose, which includes the basic demographics of the child (sex, age), the primary concerns of the parents for taking their child to the hospital, relevant data collected during the baseline and follow-up physical examinations (4, 8, 12, 24, 36, 48, 72 hours and hospital discharge), the recovery status over the course of the hospital stay, administered treatments, findings from the echocardiogram and the cranial ultrasound, primary and secondary diagnosis by the LFHC treating physicians at the child’s discharge from hospital, dietary practices and maternal characteristics. The latter include exclusive breast feeding, maternal dietary practices with regard to a restricted postpartum diet and whether the mother experiences tingling in her fingers, an indication that the mother herself may experience clinical manifestations of severe thiamine deficiency. In addition to the case report, the expert panel will be able to review additional study data as needed.

### Data management and statistics

#### Electronic data collection, data management and data quality control

Participants will be given a unique four-digit numeric study ID code, which will link all data collected from the individual. To link the data from a child–mother pair, the same four-digit ID code will be given to each with the exception that the mothers’ data will be identified with the letter ‘M’ before the four-digit ID code.

All data will be collected electronically using Survey CTO, and pre-designed, pre-coded paper forms will be available for backup, if needed. Data collection forms were developed in a customised Survey CTO application by a UC Davis data manager, and, where appropriate, forms include pre-determined checks and range validations. Forms will be deployed onto Samsung tablets (Samsung Galaxy Tab. 3 V, Seoul, South Korea) equipped for mobile internet access. All data forms collected will first be reviewed by one of two study data managers and then synchronised onto a central server.

Data quality checks will be run weekly and track information such as form completion, internal consistency, specimen sample processing, realistic data ranges, eligibility criteria, child’s timeline within the study and translation of local languages to English. Feedback will be regularly communicated to study staff to ensure consistent high-quality data collection.

#### Sample size

For our primary research question, we aim to develop and compare clinical prediction rules that are useful in determining which children with TDD-like symptoms would respond favourably to thiamine treatment. Given the context of the study, there is uncertainty around the expected prevalence of TRD in our sample and so we anticipate that between 10% and 90% of the hospitalised cohort will be classified as responders. Assuming that a clinically useful discriminative capacity will correspond to an area under the receiver operating characteristic curve (AUROC) of 0.70 for the responders versus non-responders classification, we will test the null hypothesis that the AUROC is 0.5 using a two-sided test with alpha=0.05. To account for the selection of independent variables for our multivariable model, we derived a variance inflation factor of 1.88 by computing the ratio of the χ^2^ non-centrality parameters corresponding to 95% power, two-sided alpha, with 10 and 1 df, respectively (24.386 (for 10 df) divided by 12.995 (for 1 df)). Additional variance inflation factors of 1.25 and 1.25 were needed to account for 20% loss to follow-up and 20% probability of blood collection failure, respectively. Hence, we anticipate that a total enrolment of 662 hospitalised children will, after correcting for these three variance inflation factors, result in an effective sample size of 225 for power and sample size purposes. According to the SAS ROCPOWER macro (SAS V.9.4; SAS Institute), an effective sample size of 225 would provide at least 90% power to detect a model with a discriminative capacity corresponding to an AUROC of 0.70, under two-sided testing with alpha=5% given a TRD prevalence of between 11% and 89%.

For our secondary research questions concerned with describing TRD risk factors across a community comparison cohort and our two hospital cohorts, we plan to enrol 212 region, age and sex frequency-matched children (increased to 265 after adding the inflation factor of 1.25 for potential blood collection failure). Given a hospital cohort split between TRD groups of sizes 60–106 and 424–470, this would provide at least 80% power to detect an independent variable for which a 1 SD increase is associated with an adjusted OR of 1.6, under two-sided testing (of the null hypothesis that the adjusted OR is 1) with alpha=5%, even when other covariates in the models account for as much as 20% of the variation in that independent variable. For a binary risk factor present in 20% of children, the detectable adjusted OR under the stated assumptions would be 2.45. Hence, we anticipate having adequate statistical power to detect risk factors associated with moderate to strong incremental discriminative capacity.

#### Statistical analysis

Prior to performing statistical analyses, detailed statistical analysis plans will be developed and published online.^39^

Our primary model development objective will seek to achieve case definition models that provide high discrimination capacity and acceptable calibration in external samples, using principled approaches to guard against overfitting biases. It is likely that there will be ambiguity in TRD determination due to a range of potential thresholds for what is considered ‘recovery‘. Consequently, sensitivity analyses may be repeated with different variations of TRD definitions. These definitions will be defined prior to analysis by expert paediatricians. We will follow a three-staged approach to identifying candidate risk factors and selecting among them. In the first stage, investigators will specify risk predictors that could be reliably and validly measured early in the hospitalisation in limited-resource settings and which are plausibly associated with TRD status. In the second stage, we will develop and validate logistic regression models, using TRD status as the dependent variable and using elastic net regression and related approaches to principled variable selection. In the third stage, we will repeat the analysis incorporating more sophisticated diagnostic assessments, such as biomarkers and ultrasound, which are available in higher resource settings. Finally, after each clinical prediction model is developed, we will use resampling methods with 10-fold cross validation to estimate model performance in terms of discriminative capacity, as measured by empirical AUROC, internally and externally.

Our secondary objective of establishing biomarker cut-offs for TRD will follow a similar receiver operating characteristic curve-based approach, except with specific biomarker concentrations as the principle predictor. A weighted evaluation of each cut-offs sensitivity and specificity will be used in a 10-fold cross-validation framework to select the optimal cut-off.^40^

The comparison of predictors, biomarkers and risk factors across the community cohort, non-TRD hospital cohort and TRD hospital cohort will be contextual and dependent on the type of variable considered. Analyses will be primarily descriptive, focused on substantive differences between cohorts, accompanied by visualisations and distribution-appropriate statistical testing.

## Ethics and dissemination

The executive leadership of LFHC approved the conduct of the study as described here.

The presented protocol version (Version 2019-11-12) includes changes to the inclusion criteria, described in detail above. Any future modifications to the protocol including changes of study objectives, study design, inclusion criteria, sample sizes, study procedures or significant administrative aspects will be agreed on by the principal investigators and co-investigators and approved by the National Ethics Committee for Health Research, Ministry of Health (MOH), Lao PDR and the UC Davis InstitutionalReview Board (IRB) prior to implementation. The present study will be implemented among children who will be admitted to the hospital for TDD-like symptoms. Study participants will be treated by the hospital physicians following the usual hospital procedures, and the study team will not interfere with the administration of required treatments and interventions. The one study-specific intervention administered to all hospitalised study participants is thiamine. Thiamine is a water-soluble vitamin prescribed in case of beriberi or thiamine deficiency.^10 22^ Documented side effects include nausea, flushing sensation, restlessness, sweating and weakness, and there may be hypersensitivity reaction to intravenous injection or tenderness at the intramuscular injection site.^41^ All children will be examined for these symptoms over the course of the study (table 1), and any observed adverse events will be documented. Serious adverse events that are unexpected and/or probably related to the research procedures will be reported immediately to the UC Davis IRB and the National Ethics Committee for Health Research in Lao PDR.

A Data and Safety Monitoring Board (DSMB) will be established for the purpose of this project. Three independent paediatricians will monitor patient safety while the study is ongoing. Interim analyses will be completed only for safety considerations. Reports of the occurrence of potential adverse events will be submitted to and reviewed by the DSMB members. A new report will be shared with the DSMB committee each time a quarter of study participants are enrolled. The principal investigators have the right to terminate the study if the incidence of side effects and/or severity of adverse events outweigh the benefits of the study. Since thiamine administration is an established intervention with mild temporary side effects, it is expected that the study will continue until planned completion. Detailed stopping rules have not been designed at this time.

Study results will be reported following recommendations by the International Committee of Medical Journal Editors,^42^ and in accordance with the TRIPOD Statement and other relevant guidelines from the EQUATOR Network.^43 44^ The knowledge and information gained from the present study will be promptly and broadly disseminated through peer-reviewed scientific open access articles, and through communications and presentations at conferences. Moreover, the present study is part of a larger multi-country effort to address critical questions related to thiamine deficiency and public health in Asia and Africa.^45 46^ An important aspect of the overall project is to exchange research results among the collaborators. In-country result dissemination will be led by the Lao Tropical and Public Health Institute through meetings and workshops with representatives of the MOH, the Provincial Health Departments and other key stakeholders. Research briefs will also be produced for in-country distribution. In addition, the findings will be presented at the Annual Lao Pediatric Continuing Medical Education Conference organised by the Lao Pediatric Association and at the National Health Research Forum in Vientiane, Lao PDR.

## Trial status

The first participants of the hospital and community cohort were enrolled in June 2019. Changes to the enrolment criteria were implemented in November 2019 (after 18.1% of the hospital and 13.6% of the community target sample size had been enrolled). We anticipate data collection will be completed by December 2020.

## Supplementary Material

Reviewer comments

Author's manuscript
