# Supplementary material for: Establishing a case definition of thiamine responsive disorders among infants and young children in Lao PDR: protocol for a prospective cohort study
Source: BMJ Open. 2020 Feb 13;10(2):e036539. doi: 10.1136/bmjopen-2019-036539 (PMC7044841; doi:10.1136/bmjopen-2019-036539)
Supplement: Supplementary data [file bmjopen-2019-036539supp001.pdf]

Supplementary Table 1: Inclusion criteria for study participants and outcomes of interest in the Lao Thiamine Study

**Inclusion criteria**	**Outcomes**		
	**Physical exams**	**Biochemical exams**	**Data collection via questionnaires**
Hospitalised children			
Target age range:<br>- 21 days to <18 months[1]<br><br>Plus at least one of the following criteria:<br>- Liver enlargement (>2cm below right costal margin, supine exam)<br>- OedemaTachypnea (> 60/min for 3-8 weeks; > 50/min for 2-11 moths; >40/min for 12 - 18 months)<br>- Tachycardia (heart rate >160/min for <12 months; >120/min for 12-18 months)<br>- Oxygen saturation < 92%<br>- Difficulty breathing (chest in-drawing or nasal flaring)<br>- Refusal to breastfeed or refusal of infant formula or food for greater than 24 hours<br>- Repetitive or recurrent vomiting with no obvious other cause (i.e. vomiting >3 times in past 24 hours)<br>- Persistent crying not relieved by soothing or feeding with no obvious other cause<br>- Hoarse voice/cry or loss of voice<br>- Nystagmus or other unusual eye movements<br>- Muscle twitching<br>- Loss of consciousness<br>- Convulsion<br>- Opisthotonus /abnormal posturing[2]<br>- Acute paralysis/flaccid paralysis[2]	- General physical exam performed by physician/nurse (calm, fussy, interactive; looks sick or well)<br>- Liver palpation<br>- Heart rate<br>- Exam for oedema (puffy eyelids, pitting oedema of back, genitals or ankles, whole body puffy)<br>- Respiratory distress (retractions, nasal flaring, paradoxical breathing, head bobbing)<br>- Respiratory rate, oxygen saturation, perfusion index<br>- Temperature<br>- Documentation of abnormal eye movements and eye contact<br>- Examine for signs of ptosis<br>- Hypotonia (head lag, floppy arms or legs in face-down position)<br>- Consciousness evaluation (AVPU scale)<br>- Documentation of timing of onset of each symptom<br>- Anthropometric indices (length, weight, mid-upper arm circumference, head circumference)<br>- Ultrasounds:<br>- Echocardiogram<br>- Cranial ultrasound<br><br>Follow up after thiamine administration:<br>- Repetition of physical exam after 4, 8 and 12 hours and every 12 hours for first 48 hours<br>- After 24 and 48 hrs: Echocardiogram<br>- After 72 hrs: Repetition of physical exam	- ETKac in washed RBC<br>- Whole blood ThDP<br>- CBC<br>- Troponin T<br>- Lactate<br>- Indicators of inflammation (CRP, AGP)	- Basic demographics of child (age, sex, location of residence)<br>- Socioeconomic status of mother and family<br>- Gestational age at birth<br>- Birth/perinatal history<br>- Birth weight and length<br>- History of severe illnesses since birth and hospitalisations<br>- History of any illnesses in 2 weeks prior to onset of current illness<br>- History of bowel movements in past 24 hours (number and consistency), urinary outputs in past 24 hours and constipation in previous 7 days<br>- Recent and current intake of medications, dietary supplements and use of Tiger Balm<br>- Documentation of treatments and medications prescribed due to current illness, and thiamine at discharge<br>- Vaccination record<br>- Dietary practices of child (breastfeeding, complementary feeding)<br>- Maternal report on recent changes in physical movements, motor skills and behaviour

	For DSMB:<br>- Response to thiamine injection/administration (such as local rash/redness, infection/swelling, temporary lethargy)		
Mothers of hospitalised children			
Exclude if:<br>- Severe acute illness warranting immediate hospital referral<br>- Unable to provide informed consent due to reduced decision making ability	- Maternal height, weight and mid-upper arm circumference	- ETKac in washed RBC<br>- Whole blood ThDP<br>- Indicators of inflammation (CRP, AGP)<br>- CBC<br>- Dried blood spots<br>- Breast milk thiamine concentration of lactating mothers	- Maternal characteristics (age, education, profession, marital status, ethnicity, religion, location of residence, family structure, number of children, etc.)<br>- Recent and current intake of multi-micronutrient supplements, medications and use of Tiger Balm<br>- Thiamine given at discharge<br>- Dietary practices of mother (dietary diversity questionnaire, food preparation, dietary beliefs/avoidance of certain foods, consumption of raw fish/fish paste, betel nut, tea and alcohol)<br>- Food security questionnaire<br>- History of beriberi (maternal beriberi and mother's children)<br>- Social desirability bias
Community-based age- and sex-matched children			
- Excluded if immediate hospital referral is required<br>- No other exclusion criteria	- General physical exam performed by study nurse<br>- Heart rate<br>- Respiratory rate, oxygen saturation, perfusion index<br>- Temperature<br>- Hypotonia (head lag, floppy arms or legs in face-down position)<br>- Anthropometric indices (length, weight, mid-upper arm circumference, head circumference)	- ETKac in washed RBC<br>- Whole blood ThDP<br>- CBC<br>- Indicators of inflammation (CRP, AGP)	- Basic demographics of child (age, sex, location of residence)<br>- Socioeconomic status of mother and family<br>- Gestational age at birth<br>- Birth/perinatal history<br>- Birth weight and length<br>- History of severe illnesses since birth and hospitalisations<br>- History of any illnesses in past 2 weeks<br>- History of bowel movements in past 24 hours (number and consistency), urinary outputs in past 24 hours and constipation in previous 7 days<br>- Recent and current intake of medications, dietary supplements and use of Tiger Balm<br>- Vaccination record<br>- Dietary practices of child (breastfeeding, complementary feeding)

			- Maternal report on recent changes in physical movements, motor skills and behaviour
Mothers of children in the community			
Exclude if:<br>- Severe acute illness warranting immediate hospital referral<br>- Unable to provide informed consent due to reduced decision making ability	- Maternal height and weight and mid-upper arm circumference	- ETKac in washed RBC<br>- Whole blood ThDP<br>- Indicators of inflammation (CRP, AGP)<br>- CBC<br>- Dried blood spots<br>- Breast milk thiamine concentration of lactating mothers	- Maternal characteristics (age, education, profession, marital status, ethnicity, religion, location of residence, family structure, number of children, etc.)<br>- Recent and current intake of multi-micronutrient supplements, medications and Tiger Balm<br>- Dietary practices of mother (dietary diversity questionnaire, food preparation, dietary beliefs/avoidance of certain foods, consumption of raw fish/fish paste, betel nut, tea and alcohol)<br>- Food security questionnaire<br>- History of beriberi (maternal beriberi and mother's children)<br>- Social desirability bias

AGP, α-1-acid glycoprotein; CBC, complete blood count; CRP, C-reactive protein; DSMB, data safety monitoring board; ETKac, erythrocyte transketolase activity coefficient; RBC, red blood cells; ThDP, thiamine diphosphate

[1] At the onset of the study, the eligibility age was 21 days to <12 months. The target age was expanded to <18 months after 18.1% hospital infants were enroled due to much lower enrolment rates than anticipated, and after consultation with expert paediatricians and the hospital leadership.
[2] Tachycardia, opisthotonus/abnormal posturing and acute paralysis/flaccid paralysis were added as inclusion criteria at the time when the target age range was extended to <18 months of age.
