# Supplementary material for: Establishing a case definition of thiamine responsive disorders among infants and young children in Lao PDR: protocol for a prospective cohort study
Source: BMJ Open. 2020 Feb 13;10(2):e036539. doi: 10.1136/bmjopen-2019-036539 (PMC7044841; doi:10.1136/bmjopen-2019-036539)
Supplement: Author's manuscript [file bmjopen-2019-036539.draft_revisions.pdf]

BMJ Open

BMJ Open is committed to open peer review. As part of this commitment we make the peer review history of every article we publish publicly available.

When an article is published we post the peer reviewers' comments and the authors' responses online. We also post the versions of the paper that were used during peer review. These are the versions that the peer review comments apply to.

The versions of the paper that follow are the versions that were submitted during the peer review process. They are not the versions of record or the final published versions. They should not be cited or distributed as the published version of this manuscript.

BMJ Open is an open access journal and the full, final, typeset and author-corrected version of record of the manuscript is available on our site with no access controls, subscription charges or pay-per-view fees (http://bmjopen.bmj.com).

If you have any questions on BMJ Open's open peer review process please email info.bmjopen@bmj.com

BMJ Open

Establishing a case definition of thiamine responsive disorders among infants and young children in Lao PDR: Protocol for a prospective study

Journal:	*BMJ Open*
Manuscript ID	bmjopen-2019-036539
Article Type:	Protocol
Date Submitted by the Author:	18-Dec-2019
Complete List of Authors:	Hess, Sonja; University of California Davis, Institute for Global Nutrition<br>Smith, Taryn; University of California Davis, Institute for Global Nutrition<br>Fischer, Philip; Mayo Clinic, Department of Pediatric and Adolescent Medicine<br>Trehan, Indi; University of Washington, Department of Pediatrics and Department of Global Health; Lao Friends Hospital for Children<br>Hiffler, Laurent ; Independent Consultant Pediatrician<br>Arnold, Charles D; University of California Davis, Institute for Global Nutrition<br>Sitthideth, Dalaphone; Lao Tropical and Public Health Institute<br>Tancredi, Daniel; University of California Davis Health System, Department of Pediatrics<br>Schick, Michael; University of California Davis Health System, Emergency Medicine<br>Yeh, Jay; University of California Davis Health System, Department of Pediatrics, Division of Cardiology<br>Stein-Wexler, Rebecca; University of California Davis Health System, Department of Radiology<br>McBeth, Christine; University of California Davis Health System, Emergency Medicine<br>Tan, Xiuping; University of California Davis, Institute for Global Nutrition<br>Nhiacha, Kouyang; Lao-Korea Children Hospital<br>Kounnavong, Sengchanh; Lao Tropical and Public Health Institute
Keywords:	NUTRITION & DIETETICS, Echocardiography < CARDIOLOGY, Neuroradiology < NEUROLOGY

SCHOLARONE™
Manuscripts

I, the Submitting Author has the right to grant and does grant on behalf of all authors of the Work (as defined in the below author licence), an exclusive licence and/or a non-exclusive licence for contributions from authors who are: i) UK Crown employees; ii) where BMJ has agreed a CC-BY licence shall apply, and/or iii) in accordance with the terms applicable for US Federal Government officers or employees acting as part of their official duties; on a worldwide, perpetual, irrevocable, royalty-free basis to BMJ Publishing Group Ltd ("BMJ") its licensees and where the relevant Journal is co-owned by BMJ to the co-owners of the Journal, to publish the Work in this journal and any other BMJ products and to exploit all rights, as set out in our licence.

The Submitting Author accepts and understands that any supply made under these terms is made by BMJ to the Submitting Author unless you are acting as an employee on behalf of your employer or a postgraduate student of an affiliated institution which is paying any applicable article publishing charge ("APC") for Open Access articles. Where the Submitting Author wishes to make the Work available on an Open Access basis (and intends to pay the relevant APC), the terms of reuse of such Open Access shall be governed by a Creative Commons licence – details of these licences and which Creative Commons licence will apply to this Work are set out in our licence referred to above.

Other than as permitted in any relevant BMJ Author's Self Archiving Policies, I confirm this Work has not been accepted for publication elsewhere, is not being considered for publication elsewhere and does not duplicate material already published. I confirm all authors consent to publication of this Work and authorise the granting of this licence.

For peer review only

Establishing a case definition of thiamine responsive disorders among infants and young children in Lao PDR: Protocol for a prospective study

Sonja Y Hess[1], Taryn J Smith[1], Philip R Fischer[2], Indi Trehan[3,4], Laurent Hiffler[5], Charles D Arnold[1], Dalaphone Sitthideth[6], Daniel J Tancredi[7], Michael A Schick[8], Jay Yeh[9], Rebecca Stein-Wexler[10], Christine N McBeth[8], Xiuping Tan[1], Kouyang Nhiacha[11], Sengchanh Kounnavong[6]

[1] Institute for Global Nutrition, University of California Davis, Davis, CA, USA
[2] Department of Pediatric and Adolescent Medicine, Mayo Clinic, Rochester, MN, USA
[3] Lao Friends Hospital for Children, Luang Prabang, Lao PDR
[4] Department of Pediatrics and Department of Global Health, University of Washington, Seattle, WA, USA
[5] Independent Consultant Pediatrician, Lagny sur Marne, France
[6] Lao Tropical and Public Health Institute, Ministry of Health, Vientiane, Lao PDR
[7] Department of Pediatrics, University of California Davis Health, Sacramento, CA, USA
[8] Emergency Medicine, University of California Davis Health, Sacramento, CA, USA
[9] Department of Pediatrics, Division of Cardiology, University of California Davis Health, Sacramento, CA, USA
[10] Department of Radiology, University of California Davis Health, Sacramento, CA, USA
[11] Lao-Korean Children Hospital, Vientiane, Lao PDR

Correspondence to: Sonja Y. Hess, Institute for Global Nutrition, University of California Davis, One Shields Avenue, Davis, CA 95616, USA. Email: syhess@ucdavis.edu Phone: 530 752 1992

Co-author contact information:
Sonja Y Hess: syhess@ucdavis.edu
Taryn J Smith: tjsm@ucdavis.edu
Philip R Fischer: Fischer.Phil@mayo.edu
Indi Trehan: indi@alum.berkeley.edu
Laurent Hiffler: Laurenthiffler@gmail.com
Charles D Arnold: cdarnold@ucdavis.edu
Dalaphone Sitthideth: dsitthideth@yahoo.com
Daniel J Tancredi: djtancredi@ucdavis.edu
Michael A Schick: mschick@ucdavis.edu
Jay Yeh: jayyeh@ucdavis.edu
Rebecca Stein-Wexler: rsteinwexler@ucdavis.edu

For peer review only
For peer review only - http://bmjopen.bmj.com/site/about/guidelines.xhtml

Christine N McBeth: cmcbeth@UCDAVIS.EDU
Xiuping Tan: xptan@ucdavis.edu
Kouyang Nhiacha: nhiacha.kouyang@gmail.com
Sengchanh Kounnavong: sengchanhkounnavong@hotmail.com

Word count: 7073

Word limit: 4000

Key words: Thiamine deficiency, beriberi, infant, children, Lao PDR

For peer review only

ABSTRACT

Introduction Diagnosis of infantile thiamine deficiency disorders (TDD) is challenging due to the non-specific, highly variable clinical presentation, often leading to misdiagnosis. Our primary objective is to develop a case definition for thiamine responsive disorders (TRD) to determine among hospitalised infants and young children, which clinical features and risk factors identify those who respond positively to thiamine administration.

Methods and analysis This prospective study will enrol 662 children (aged 21 days - <18 months) seeking treatment for TDD symptoms. Children will be treated with intravenous or intramuscular thiamine (100mg daily for a minimum of 3 days) alongside other interventions deemed appropriate. Baseline assessments, prior to thiamine administration, include a physical exam, echocardiogram and venous blood draw for determination of thiamine biomarkers. Follow up assessments include physical exams (after 4, 8, 12, 24, 36, 48 and 72 hours), echocardiogram (after 24 and 48 hours) and one cranial ultrasound. During the hospital stay, maternal blood and breastmilk samples and diet, health, anthropometric and sociodemographic information will be collected for mother-child pairs. Using this data, a panel of expert paediatricians will determine TRD status for use as the dependent variable in logistic regression models. Models identifying predictors of TRD will be developed and validated for various scenarios. Clinical prediction model performance will be quantified by empirical area under the receiver operating characteristic curve, using resampling cross-validation. A frequency matched community-based cohort of mother-child pairs (n = 265) will serve as comparison group for evaluation of potential risk factors for TRD.

Ethics and dissemination Ethical approval has been obtained from The National Ethics Committee for Health Research, Ministry of Health, Lao PDR and the Institutional Review Board of the University of California Davis. Results will be disseminated via scientific articles, presentations and workshops with representatives of the Ministry of Health.

Trial registration number NCT03626337.

For peer review only

For peer review only - http://bmjopen.bmj.com/site/about/guidelines.xhtml

ARTICLE SUMMARY

STRENGTHS AND LIMITATIONS OF THIS STUDY

- A standardised case definition for thiamine responsive disorder (TRD) will be proposed, through understanding of the interplay between clinical presentation, response to therapeutic thiamine administration and biochemical analyses, which will help more accurately diagnose infantile TRD.
- The broad spectrum of eligibility criteria will ensure inclusion of hospitalised infants and young children who may have milder, underlying thiamine deficiency and may respond clinically and benefit from thiamine administration, as well as those with suspected beriberi.
- Associations between TRD and biomarkers of thiamine status will help establish deficiency cut-off thresholds for thiamine diphosphate (ThDP) and erythrocyte transketolase activity coefficient (ETKac) that are relevant to a population with thiamine deficiency.
- Due to ethical considerations, the study does not include a placebo control group and will rely on a community comparison group to cross-check risk factors identified in the hospitalised cohort to a healthier population.
- In addition to the thiamine administration, hospitalised children will continue to receive routine care, such as antibiotics and other medications for concurrent illnesses that may affect the children’s recovery, making the clear distinction between TRD cases and non-responders more challenging.

For peer review only

For peer review only - http://bmjopen.bmj.com/site/about/guidelines.xhtml

INTRODUCTION

Thiamine (vitamin B1) is an essential micronutrient, required as a cofactor for several enzymes involved in carbohydrate metabolism and energy production. Thiamine deficiency has been described in several low and middle income countries and remains a public health concern in many countries of Southeast Asia[1,2] , including Lao People's Democratic Republic (Lao PDR)[3-5]. Diets that lack in dietary diversity and rely too heavily on low-thiamine staple crops (such as polished white rice), food preparation practices (repetitive washing and soaking rice for many hours) and traditional postpartum restrictive diets, all place lactating women at high risk of thiamine deficiency in Lao PDR[6-8]. Breastmilk thiamine concentrations and infant thiamine status are strongly dependent on maternal thiamine intake and status, and maternal thiamine deficiency rapidly results in low breastmilk thiamine concentrations[9]. Infants are therefore especially vulnerable to thiamine deficiency in the first months of life, and exclusively breastfed infants of thiamine-deficient mothers are at the highest risk. According to the World Health Organization (WHO), infantile thiamine deficiency is the only serious form of malnutrition that occurs in adequately breastfed infants and only when the mother herself is thiamine deficient[10].

Thiamine deficiency presents as a broad spectrum of non-specific clinical abnormalities, referred to as thiamine deficiency disorders (TDD), of which the classic forms of beriberi in adults and children are best known, although even classic beriberi often goes unrecognised[11]. TDD can be characterised to some degree by age and features of clinical presentation[2]. Infantile TDD commonly occurs between 1 and 9 months of age, with non-specific early signs such as refusal to breastfeed, vomiting and irritability (1-3 months of age), which can progress to oedema, respiratory distress, heart failure and death if not rapidly treated. During later infancy, the cry may sound hoarse or change to a soundless cry (4-6 months of age) and neurological symptoms may present, including nystagmus, convulsions and unconsciousness (7-9 months of age). Infants with encephalitic beriberi were found to have abnormal cranial ultrasonography, with normalisation within 8 weeks of thiamine administration[12]. In the second year of life and later in childhood, TDD more typically presents with a broad range of neurological symptoms, such as anorexia, irritability, agitation, muscle pain, reduced deep tendon reflexes, ataxia, paralysis and altered levels of consciousness [11,13-15]. Due to uncertainties regarding the diagnostic criteria and overlapping symptoms with other conditions, TDD may frequently be misdiagnosed as bronchiolitis, pneumonia, typhoid fever, sepsis, meningitis or encephalitis, and the underlying thiamine deficiency may remain untreated[1,2,11]. Cases of TDD are often suspected rather than confirmed, and a final diagnosis is usually based upon a positive response to intramuscular or intravenous thiamine administration.

For peer review only - http://bmjopen.bmj.com/site/about/guidelines.xhtml

Diagnosis of TDD is further complicated by the fact that several studies have found little association between biomarkers of thiamine status and TDD-like symptoms[16-18]. It has been suggested that low thiamine status alone cannot reliably discriminate between symptomatic and asymptomatic infants, and infectious co-pathologies and other physiological stressors may precipitate the onset of TDD[17]. Conversely, low thiamine status appears to increase vulnerability to disease, as indicated by the observation that hospitalised infants with low thiamine status, but no clinical signs of beriberi, showed higher mortality rates in a region of Lao PDR[4].

Thiamine status can be determined by assessing thiamine metabolites in blood or urine. The most widely used biochemical indicator of thiamine status is the activity of the thiamine dependent transketolase enzyme in erythrocytes (ETK), which reflects the biological functionality of the vitamin and is sensitive to marginal thiamine deficiency[19]. Thiamine diphosphate (ThDP) concentration in whole blood or erythrocytes is also a useful indicator as ThDP is the biologically active vitamer and is reflective of body stores. There are however many challenges to assessing biomarkers of thiamine status, especially in resource-limited settings, including cost, the required cold chain and limited availability of laboratories able to analyse biological specimens. Hence, thiamine biomarkers are not routinely assessed and a rapid positive response to therapeutic thiamine is considered confirmation of a deficient status. Furthermore, there are currently no universally agreed upon adequacy or deficiency cut-off thresholds for erythrocyte or whole blood ThDP or ETK activity coefficient (ETKac), which have largely been studied in thiamine adequate adults in high-income countries. Consequently a wide range of cut-offs has been used[1]. Limited data derived from two studies in European adults at risk of thiamine deficiency from either chronic alcoholism or from an acute or chronic medical condition are available on associations between ETKac and ThDP[20,21]. These studies suggest that elevated ETKac (≥ 25%) occurs when ThDP concentrations are below 280 mg/g haemoglobin. However, it is not clear if similar results would be apparent in other populations. Thus, there is a need to develop clinically meaningful cut-off thresholds for biomarkers indicative of thiamine deficiency in other at-risk populations, especially infants and young children in countries where thiamine intake is inadequate and exclusive breastfeeding prevalent.

In summary, owing to the wide variability in clinical presentation, a barrier for reliable diagnosis and targeted treatment of TDD is the lack of a clear clinical case definition with broad consensus. There is a need to understand the interaction between thiamine status, exposure to risk factors (including infections) and onset of clinical symptoms in areas where thiamine deficiency is prevalent. Thus, this study aims to develop a

For peer review only - http://bmjopen.bmj.com/site/about/guidelines.xhtml

standardised case definition for thiamine responsive disorders (TRD), based on a combination of presenting clinical features most predictive of responding to therapeutic thiamine doses, alongside assessment of biochemical indicators of thiamine status and potential risk factors, in infants and young children aged 21 days to <18 months in Lao PDR.

OBJECTIVES OF STUDY

The study's primary objective is the development of a case definition for TRD among infants and young children with symptoms consistent with TDD, with the case definition being based on those clinical symptoms and other predictors that we find are better able to distinguish those who respond positively to thiamine administration from those who do not respond. As diagnostic tools available to treating physicians may differ by setting, we will repeat analyses after excluding from the set of candidate predictors those that are derived from assessments only available in higher-resource settings, such as laboratory biomarkers and ultrasonography.
A secondary objective is to fill the knowledge gap surrounding biomarkers of thiamine status in at-risk populations. Biomarker cut-offs for TRD will be developed in the hospital cohort and distributions will be characterised in both the hospital and community cohorts. The performance of our proposed cut-off will be compared to the performance of existing literature cut-offs.
Additionally, all identified predictors and biomarker cut-offs will be compared across the community cohort, non-TRD hospital cohort, and TRD hospital cohort to assess the prevalence of risk factors among apparently healthy infants and young children and assess the usefulness of the TRD case definition in various settings.

For peer review only

METHODS AND ANALYSIS

Study design and procedures

Study design

For peer review only - http://bmjopen.bmj.com/site/about/guidelines.xhtml

The study uses a prospective study design and includes a cohort of infants and young children 21 days to <18 months of age who are identified as having TDD-compatible symptoms (**Figure 1**). Children will be recruited at the Lao Friends Hospital for Children (LFHC) in Luang Prabang, Lao PDR, and potentially eligible children presenting with any of the below described inclusion criteria will be identified through close collaboration between the LFHC hospital staff and the study team. Enroled children will be treated with thiamine and all other clinically indicated treatments and followed closely over 48 – 72 hours, as described in more detail below. Based on the response to thiamine, children will either be defined as TRD cases or non-responders.

A community-based cohort of children frequency matched by age, sex and village of residence will be included in the study to serve as a comparison group for evaluation of potential predictors of TRD and thiamine deficiency in a non-hospitalised cohort. We will aim to achieve a comparison group that has a similar distribution of age, sex and location of residence as the hospitalised children, to minimise confounding biases. To achieve a well-balanced match, we will summarise the characteristics of the hospitalised children and enrol frequency matched community-based children weekly, to avoid seasonal discordance. Prior to visiting the health district, the head of the health centre and the head of the village will be contacted to ask for assistance in identifying a child of the right target age (within a 2-week period) and living in a selected village or, if not available, a neighbouring village. The health centre staff will establish contact with the child's parents/caregivers, briefly explain the study and invite the parents/caregivers for a study appointment at the health centre. All mothers of participating children in the hospital and community will also be invited into the study to obtain in-depth information on potential risk factors, including maternal thiamine status and thiamine concentration in breastmilk.

Inclusion and exclusion criteria

Inclusion and exclusion criteria of children at the hospital

Initially, infants 21 days to <12 months of age were the target population for the present study. This target age range was chosen because the risk of beriberi is highest during infancy, with highest mortality rates between 2-4 months of age[10]. Very young infants from 0 – 20 days of age are not eligible, because beriberi is rarely reported in the first few weeks of life and we did not want to include very young infants at greater risk of other serious illnesses, such as newborn sepsis, which could confound the study findings. The target age range was expanded to <18 months of age after 18.1% of the target hospital sample size had been enroled. This extension

For peer review only - http://bmjopen.bmj.com/site/about/guidelines.xhtml

For peer review only

of eligibility criteria was implemented due to much lower enrolment rates than anticipated in the first few months of the study, after further review of the scientific literature, and in consultation with expert paediatricians and the hospital leadership. Thus, throughout the majority of the study duration, the target age is 21 days to <18 months.

Children in the target age range, who are admitted to inpatient care at LFHC, will be screened by a study physician and/or study nurse to determine the presence of at least one of the following inclusion criteria:

- Liver enlargement (>2cm below right costal margin, supine exam)
- Oedema
- Tachypnea (respiratory rate > 60/min for 3-8 weeks; > 50/min for 2-11 months; >40/min for 12 – 18 months)
- Tachycardia (heart rate >160/min for <12 months; >120/min for 12-18 months)
- Oxygen saturation < 92%
- Difficulty breathing (chest in-drawing, or nasal flaring)
- Refusal to breastfeed or refusal of infant formula or food for greater than 24 hours
- Repetitive or recurrent vomiting with no obvious other cause (i.e. vomiting >3 times in past 24 hours)
- Persistent crying not relieved by soothing or feeding with no obvious other cause
- Hoarse voice/cry or loss of voice
- Nystagmus or other unusual eye movements
- Muscle twitching
- Loss of consciousness
- Convulsion
- Opisthotonus/abnormal posturing
- Acute paralysis/flaccid paralysis

The list of inclusion criteria has been developed based on TDD-compatible signs and symptoms in the broadest sense, in an effort to reduce the risk of potentially omitting children who would respond clinically to thiamine administration to correct any deficiencies. Tachycardia and two other inclusion criteria (opisthotonus/abnormal posturing and acute paralysis/flaccid paralysis) were added once the target age range was extended to <18 months of age to capture the full range of neurological signs and symptoms that might occur in the second year of life.

For peer review only - http://bmjopen.bmj.com/site/about/guidelines.xhtml

Inclusion and exclusion criteria of children in the community

The eligibility age for the community children is identical to that of the hospital. Thus, initially the target age range was 21 days to <12 months, and this was expanded to 21 days to <18 months after 13.6% of the community sample size had been enroled. Other inclusion criteria for the community cohort are that community children match the characteristics of the hospitalised children with regard to age, sex and community of residence characteristics. Children are not excluded based on any ineligibility criteria, except in case of severe acute illness warranting immediate hospital referral.

Inclusion and exclusion criteria of study participants' mothers

Once the hospitalised and community-based children are enroled, their mothers will also be invited for study participation, unless the women meet one of the following exclusion criteria: 1) severe acute illness warranting immediate hospital referral; or 2) unable to provide informed consent due to reduced decision-making ability.

Informed consent

Informed consent will be obtained from at least one primary caregiver (parent or legal guardian) of children for the child's study participation as well as the mothers (or other female primary caregiver) for their own study participation. Because the study activities vary depending on the study participant (child *vs*. mother) and the location (hospital *vs*. community), different consent forms will be used. If the parents and/or mother cannot read, an impartial witness will be present during the entire consent discussion to attest that the information in the consent form was accurately explained. If the parents do not understand Lao, but speak Hmong, a Hmong speaking study team member will lead the consenting procedure. In case of other languages, an individual will be identified to help translate all information into the appropriate language. The consent form will be read with the parents/mother and all information will be provided ensuring that the parents/mother understand what it will be like for their child or the mother, respectively, to participate in the study, and that the choice to participate will not affect the medical care that the child will receive while in the hospital or at any health centre. If the primary caregivers decide for the child to participate, one parent, either the mother, father or a legal guardian, or both, will be asked to sign or fingerprint the consent form of the child. The mother will be asked to sign or fingerprint her own consent form to confirm voluntary study participation. An additional

For peer review only - http://bmjopen.bmj.com/site/about/guidelines.xhtml

signature or fingerprint will be asked to confirm voluntary consent to storage and future analyses of biological specimen.

Once a child is identified as potentially eligible during the initial physical exam by the hospital medical team, a brief summary of the research study will be provided to explore the interest in study participation. In case the parent expresses an initial interest, and the child's health is stable, a study team member will give a copy of the approved consent form translated into Lao to the parent and the above described consenting procedure will be implemented. The same consenting procedure will be followed, but delayed, if the child's condition is critical or life threatening. In case the child's health does not allow postponing any exam or treatment, the child will be examined by a study physician and/or nurse and all health information will be recorded. A venous blood sample will be drawn and treatments (including intravenous or intramuscular thiamine) will be initiated prior to obtaining parental consent. Parental consent will be obtained as soon as the child's condition has stabilised, following the above described procedures. Any data and blood specimen that were collected prior to consent will only be used if parental written consent is obtained.

Intervention

Children in the hospital cohort will be treated by LFHC staff following standard care practices at the discretion of the primary medical team. The only intervention provided in the present study is thiamine. There are currently no evidence-based recommendations for thiamine dosage to treat infants and young children with severe acute illness[11]. Treatment guidelines in the pocket book of the WHO Western Pacific Region recommend treating an infant with heart failure with 25 mg thiamine intravenously and 25 mg intramuscularly, then 25 mg thiamine daily until the infant can eat, followed by 10 mg oral thiamine supplementation per day for 2 – 3 weeks[22]. Considering that previous studies used dosages ranging from 50 mg to 1500 mg depending on the clinical condition[17,23-26], and neurological presentations of TDD tend to require higher doses and longer recovery time[11], a daily dose of 25 - 50 mg was considered potentially insufficient. Consequently, daily doses of 100 mg thiamine, the standard treatment procedure of infants presenting with TDD at the LFHC, was deemed most appropriate for the present study. All children in the hospital cohort will be treated with a daily dose of 100 mg thiamine by intramuscular or intravenous administration for a minimum of three days. In general, intravenous dosing will be preferred, unless intravenous access cannot be obtained after several

For peer review only - http://bmjopen.bmj.com/site/about/guidelines.xhtml

attempts by LFHC nurses. Vials containing 100 mg thiamine as thiamine hydrochloride will be obtained locally (CBF Pharmaceutical Factory, Ban Kang, Pakse District, Lao PDR).

Procedures

Procedures at the hospital

Once the LFHC medical team have assessed the health of a potentially eligible patient, the study physician and/or nurse will complete the first standardised physical exam (i.e. baseline health status) by following a structured data collection form. The physical exam includes questions for caregivers on the child's recent health history, assessment of the child's general appearance, presence of cyanosis, breathing difficulties, bulging fontanelle, hypotonia, unusual eye movements, ptosis, sound of the child's voice/cry, muscle twitching, level of consciousness (assessed by the "alert, response to voice, response to pain, unresponsive" (AVPU) scale[22]), liver palpation and oedema. Vital signs, such as body temperature (Thermo Buddy Infrared Ear Thermometer TB-100, HuBDIC Co. Ltd., Anyang-si, Gyeonggi-do, Korea) and oxygen saturation of arterial haemoglobin, pulse rate, perfusion index, and respiration rate from pulse oximeter plethysmogram analysis will be measured using a Rad-G™ Pulse Oximeter (Masimo, Irvine, CA, USA) (Supplementary Table 1). A venous blood sample will be drawn and an echocardiogram will be performed, as described in more detail below. These data collection activities are performed prior to the first thiamine administration, unless the child's condition is critical, in which case the echocardiogram may be delayed until after the LFHC medical staff have initiated the thiamine administration and any appropriate treatments and the child's health is more stable. The date and time of each activity will be recorded for later consideration during statistical analyses. A cranial ultrasound will be performed during the hospital stay, typically within the first 12 hours.

The data collection will follow a structured timeline after the first thiamine dose has been administered (**Table 1**). In particular, the thiamine administration will be defined as hour zero, and the above described physical exam will be repeated 4, 8, 12, 24, 36, 48, and 72 hours after the initial thiamine administration (**Figure 2**). At each follow up time point, recovery status will also be assessed. Namely, at 4, 8, 12, 24, 36, 48, and 72 hours the child's health status will be described by the study physicians/nurses using a 5-point scale (seems worse; no change; seems a little bit better; seems a lot better; seems recovered). In addition, during the follow up exam after 24, 36, 48 and 72 hours, the study physicians will consult with the LFHC treating physicians whether the child is well enough to be discharged from the hospital. If a child's health has stabilised before 48 hours, the

For peer review only - http://bmjopen.bmj.com/site/about/guidelines.xhtml

parents will be asked if they are willing to remain at the hospital until 48 hours. If a child's health has stabilised by 48 hours and is ready to be discharged, direct observation will end after 48 hours. Otherwise data collection will continue until 72 hours. If a patient is unstable after 72 hours, he/she will remain at the hospital for further consultation by the hospital, but no further data will be collected until a final physical exam at discharge. Children who show abnormalities in the ultrasound scans that may be due to suspected thiamine deficiency and those with neurological symptoms at the time of discharge will be invited for a follow up physical exam after 2-6 weeks.

Procedures in the community

After obtaining written consent for the child's and mother's participation, a study nurse will perform a brief physical exam of the child, assessing the same vital signs as for hospitalised children (Supplementary Table 1). A venous blood sample will be collected from both child and mother for immediate processing in a mobile laboratory, anthropometric measurements will be conducted, breastmilk collected and questionnaires completed.

Children will be referred to the nearest health centre in the case of severe acute malnutrition (weight-for-length z-score <-3 SD[27]) and acute illnesses. In case of haemoglobin concentrations <100 g/L in both children and their mothers, the parent of the anemic child and/or the anemic mother will be informed by phone and referred to the nearest health centre for moderate or severe anaemia.

Blood sample collection

Venous blood will be collected from children and their mothers by trained nurses or laboratory technicians. Prior to the first thiamine administration, 5 mL venous blood will be collected from children into 6 mL BD vacutainer® tubes coated with K_2 EDTA (ref 367863, Becton, Dickinson & Company, Franklin Lakes, NJ, USA). From the children's mothers, 10 mL venous blood samples will be collected into 10 mL BD vacutainer® tubes coated with K_2 EDTA (ref 368589, Becton, Dickinson & Company, Franklin Lakes, NJ, USA). Blood samples will be processed immediately after they are collected, either at the hospital laboratory or in a mobile field laboratory station that will be established in each community. In particular, efforts will be made to analyse the blood for

For peer review only - http://bmjopen.bmj.com/site/about/guidelines.xhtml

lactate concentration within 30 minutes and to wash the red blood cells within 2 hours of the blood draw, as described in more detail below.

Fresh venous blood will be used for determination of the lactate concentration by StatStrip® Lactate (Nova Biomedical, Waltham, MA, USA) among hospitalised children, and analysis of the complete blood count (CBC) by haematology analyser (BC-3000 Plus Auto Hematology Analyzer, Shenzhen Mindray Bio-Medical Electronics Co. Ltd., Nanshan, Shenzhen, China). In the community, a small whole blood aliquot will be stored at 4-10°C and analysed for CBC upon arrival at the hospital laboratory, and within 8 hours of blood collection. For later assessment of ThDP, whole blood will be aliquoted into amber microcentrifuge tubes and stored at -80°C. From a convenience sample of mothers (n~200) with a complete 10 mL blood sample, 75 µl of blood will be pipetted into each of five circles of a dried blood spot card (Whatman 903 Protein Saver).

The remaining blood will be centrifuged at 3200 rpm for 10 minutes (DM0412S, DLAB Scientific Co., Ltd., Beijing, China). This speed corresponds to 1202 x g for the children's and to 1278 x g for the mothers' vacutainer tubes, respectively. Plasma will be aliquoted into microcentrifuge tubes of various volumes (0.2 to 1.5 mL) and types (amber and clear). After transferring the entire buffy coat carefully into a 1.5 mL microcentrifuge tube, packed erythrocytes will be washed three times in physiological saline (0.9% NaCl) and centrifuged at 4000 rpm for 10 minutes each time, which corresponds to 1878 x g and 2057 x g for children's and mother's tubes. All samples aliquoted in the community for later assessment of thiamine and other nutritional and health indicators will be frozen in an electronic cool box at -20°C and transferred to the -80°C freezer upon arrival at the hospital.

Blood biomarker analysis

The concentration of ThDP will be measured using the well-established thiochrome reaction coupled with high-performance liquid-chromatography fluorescence detection method[28,29]. The assay of ETK will be completed by UV spectrophotometer at the NIHR BRC Nutritional Biomarker Laboratory at the University of Cambridge in the United Kingdom. The activity of transketolase is measured in several steps: 1) the basal activity of transketolase is measured in erythrocytes (basal ETK); 2) the ETK activity is then determined in-vitro by adding exogenous thiamine diphosphate in excess (ETK activity), and; 3) the ETKac is then calculated by dividing the ETK activity by the basal ETK. C-reactive protein (CRP) and alpha-1-acid-glycoprotein (AGP) will be analysed by the enzyme-linked immunosorbent assay (ELISA).

For peer review only - http://bmjopen.bmj.com/site/about/guidelines.xhtml

For peer review only

Breastmilk sample collection and biomarker analysis

Breastmilk collection will be attempted from all lactating mothers. Because mothers of children with suspected beriberi are routinely prescribed oral thiamine supplementation at LFHC (100 mg twice daily for 30 days as per WHO recommendations[22]), breastmilk and maternal blood will be collected prior to any supplement intake.

Prior to breastmilk collection, the breast will be cleaned with a sanitary wipe (Hygea® Obstetrical Towelette, PDI Healthcare, Orangeburg, NY, USA). Breastmilk of the fuller breast will be collected into a breastmilk bag with an electronic hospital-grade breast pump (Symphony pump, Medela AG, Baar, Switzerland) until the breast is emptied. Because circadian differences in concentrations of thiamine and other vitamins are small[28], the breastmilk sample will be collected at any time of day. Time of day, time since last meal, breast side, time since last emptying of that side, and milk volume will be recorded. Samples will be mixed well before aliquoting into 1.5 mL amber and clear microcentrifuge tubes and frozen at -80°C until analysis at the USDA Western Human Nutrition Research Center at UC Davis (USA). Samples will be analysed for thiamine concentration by ultra-performance liquid chromatography tandem mass-spectrometry using internal pooled breastmilk samples for quality control[30].

Specimen banking

Any remaining biochemical samples (whole blood, red blood cells, plasma, dried blood spots and breastmilk) will be stored at UC Davis for possible future analyses of indicators of interest.

Ultrasound performance and interpretation

An echocardiogram and cranial ultrasound will be performed with a portable ultrasound machine (M9, Shenzhen Mindray Bio-Medical Electronics Co. Ltd., Nanshan, Shenzhen, China) in all hospitalised children. For the echocardiogram, study physicians will capture two second video clips of the heart in apical four chamber, parasternal long axis and parasternal short axis views. Required still images for standard measurements will include: left ventricular dimensions in diastole and systole by M-mode, E-point septal separation in the parasternal long axis, and tricuspid annular plane systolic excursion[31]. For the cranial ultrasound, study physicians will capture six standard coronal and five standard sagittal still images of the brain, with additional

For peer review only - http://bmjopen.bmj.com/site/about/guidelines.xhtml

images of the putamina, caudate nuclei, and thalamus. Cranial ultrasound will primarily be performed among infants. However, we will attempt cranial ultrasound among >12 months old children in case the anterior fontanelle has not yet closed entirely, which may allow ultrasonography of the basal ganglia. The echocardiogram will be completed at baseline (i.e. prior to or soon after the first thiamine administration) and repeated at 24 and 48 hours. Because normalisation of encephalitic beriberi occurs slowly[12], the cranial ultrasound will be performed once during the hospital stay, typically within the first 12 hours.

All ultrasonography completed by study physicians or nurses will be uploaded to Box (Box Inc, Redwood City, CA, USA). All baseline echocardiograms and cranial ultrasounds will be reviewed by one of two expert sonographers at UC Davis. If the baseline echocardiogram is flagged as abnormal by one of these reviewers, the 24 and 48 hour echocardiograms will also be reviewed by these same reviewers. In addition, all abnormal echocardiograms and cranial ultrasounds will be reviewed by a pediatric cardiologist and a pediatric radiologist at UC Davis respectively, who will provide a description of the observed abnormality. Any abnormality identified by the pediatric cardiologist and/or pediatric radiologist will be communicated to the medical leadership of the LFHC to initiate appropriate action. In case of abnormalities suggestive of thiamine deficiency, the study children will be invited for a follow up exam after 2-4 weeks in case of an abnormal echocardiogram and after 6 weeks in case of an abnormal cranial ultrasound, respectively.

For quality assurance, 10% of echocardiograms and cranial scans judged as normal will be randomly selected and re-read by one of the pediatric cardiologists and the pediatric radiologist at UC Davis, respectively. If an echocardiogram is judged as normal during the first review, the 24 and 48 hour echocardiograms will typically not be reviewed. However, 10% of randomly selected 24 and 48 hour echocardiograms will be re-read by the UC Davis expert sonographers to verify the description of the baseline echocardiography.

Anthropometric assessment

Anthropometry of both children and their mothers will be assessed in the hospital and the community following protocols proposed by the Food and Nutrition Technical Assistance III Project[32]. Unclothed or lightly dressed children will be weighed to the nearest 20 g (SECA 383, Hamburg, Germany). Children's recumbent length (SECA 417, Hamburg, Germany), left mid-upper am circumference (MUAC; Shorr Child MUAC Tape, Weigh and Measure, Olney, MD, USA) and head circumference (ShorrTape© 65cm Measuring Tape, Weigh and Measure, Olney, MD, USA) will be measured to the nearest 0.1 cm. All measurements will be

For peer review only - http://bmjopen.bmj.com/site/about/guidelines.xhtml

For peer review only

collected in duplicate and the average of the two measurements will be calculated. If measurements differ by >0.1 kg (weight), or >0.5 cm (length, MUAC, head circumference), the measurement will be repeated a third time and the average of the two closest measurements will be calculated. Maternal weight (SECA 874, Hamburg, Germany), height (SECA 213, Hamburg, Germany) and left MUAC (ShorrTape© 65cm Measuring Tape) will be assessed using a similar approach. Regular standardisation sessions will be implemented to compare the performance of anthropometry teams among themselves and with their supervisors[33,34].

Risk factor and predictor assessment

A broad range of information will be collected using structured questionnaires (**Supplementary Table 1**). Data collection forms were developed in English, and programmed in Survey CTO (Dobility Inc., Cambridge, MA, USA) and translated into Lao for electronic data collection.

Specific questionnaires were developed to assess the child's birth history, and the child and mother's health history. Because traditional restrictive postpartum diets are very common in the study population, an extensive questionnaire was developed to record dietary patterns both during pregnancy and the postpartum period. Other questionnaires, such as the assessment of socio-economic status, and child and mother dietary practice questionnaires, were adapted from the Lao Zinc Study[35]. Because women and children may change their dietary intake due to their presence at the hospital in Luang Prabang, we will ask the mother to report food intake for the days prior to their arrival at the hospital. Household food security will be assessed using the Household Food Insecurity Access Scale[36], and social desirability of respondents will be assessed as proposed by Crowne and Marlowe [37,38]. Two brief questionnaires will be used to record the reason for exiting the study by children and mothers, respectively (i.e. completion of data collection, withdrawal of consent, death, or other reasons).

Patient and public involvement

Patients and the public were not involved in the design of this study.

For peer review only - http://bmjopen.bmj.com/site/about/guidelines.xhtml

Case report review

To develop a clinician guided diagnostic impression of TRD, three expert paediatricians specialised in tropical medicine will review the case reports of each hospitalised child to judge whether a child had TRD using a 3 point scale (probable TRD (including classic beriberi); possible TRD; not likely TRD). Specifically, each case report will be reviewed by three expert paediatricians. When there is disagreement (defined as separate raters classifying the same case as probable TRD and not likely TRD) or when there is no agreement (i.e. each rater classifies a case into a different category) efforts will be made to reach consensus between the three experts. A case report template was developed for this purpose, which includes the basic demographics of the child (sex, age), the primary concerns of the parents for taking their child to the hospital, relevant data collected during the baseline and follow up physical exams (4, 8, 12, 24, 36, 48, 72 hrs and hospital discharge), the recovery status over the course of the hospital stay, administered treatments, findings from the echocardiogram and the cranial ultrasound, primary and secondary diagnosis by the LFHC treating physicians at the child's discharge from hospital, dietary practices and maternal characteristics. The latter include exclusive breastfeeding, maternal dietary practices with regard to a restricted postpartum diet and whether the mother experiences tingling in her fingers, an indication that the mother herself may experience clinical manifestations of severe thiamine deficiency. In addition to the case report, the expert panel will be able to review additional study data as needed.

Data management and statistics

Electronic data collection, data management and data quality control

Participants will be given a unique 4-digit numeric study ID code, which will link all data collected from this individual. To link the data from a child mother pair, the same 4-digit ID code will be given to each with the exception that the mothers' data will be identified with the letter "M" before the 4-digit ID code.

All data will be collected electronically using Survey CTO, and pre-designed, pre-coded paper forms will be available for back up, if needed. Data collection forms were developed in a customised Survey CTO application by a UC Davis data manager, and, where appropriate, forms include pre-determined checks and range validations. Forms will be deployed onto Samsung tablets (Samsung Galaxy Tab. 3 V, Seoul, South Korea)

For peer review only - http://bmjopen.bmj.com/site/about/guidelines.xhtml

equipped for mobile internet access. All data forms collected will first be reviewed by one of two study data managers and then synchronised onto a central server.

Data quality checks will be run weekly and track information such as form completion, internal consistency, specimen sample processing, realistic data ranges, eligibility criteria, child's timeline within the study, and translation of local languages to English. Feedback will be regularly communicated to study staff to ensure consistent high quality data collection.

Sample size

For our primary research question, we aim to develop and compare clinical prediction rules that are useful in determining which children with TDD-like symptoms would respond favourably to thiamine treatment. Given the context of the study, there is uncertainty around the expected prevalence of TRD in our sample and so we anticipate that between 10 to 90% of the hospitalised cohort will be classified as responders. Assuming that a clinically useful discriminative capacity will correspond to an area under the receiver operating characteristic curve (AUROC) of 0.70 for the responders *vs.* nonresponder classification, we will test the null hypothesis that the AUROC is 0.5 using a two-sided test with alpha = 0.05. To account for the selection of independent variables for our multivariable model, we derived a variance inflation factor of 1.88 by computing the ratio of the Chi-square noncentrality parameters corresponding to 95% power, 2-sided alpha, with 10 and 1 degrees of freedom, respectively (24.386 (for 10 df) divided by 12.995 (for 1 df)). Additional variance inflation factors of 1.25 and 1.25 were needed to account for 20% loss to follow-up and 20% probability of blood collection failure, respectively. Hence, we anticipate that a total enrolment of 662 hospitalised children will, after correcting for these three variance inflation factors, result in an effective sample size of 225 for power and sample size purposes. According to the SAS ROCPOWER macro (SAS 9.4; SAS Institute), an effective sample size of 225 would provide at least 90% power to detect a model with a discriminative capacity corresponding to an AUROC of 0.70, under two-sided testing with alpha=5% given a TRD prevalence of between 11 and 89%.

For our secondary research questions concerned with describing TRD risk factors across a community comparison cohort and our two hospital cohorts, we plan to enrol 212 region, age and sex frequency-matched children (increased to 265 after adding the inflation factor of 1.25 for potential blood collection failure). Given a hospital cohort split between TRD groups of sizes 60 to 106 and 424 to 470, this would provide at least 80% power to detect an independent variable for which a 1-standard deviation increase is associated with an adjusted odds ratio of 1.6, under two-sided testing (of the null hypothesis that the adjusted odds ratio is 1)

For peer review only - http://bmjopen.bmj.com/site/about/guidelines.xhtml

with alpha=5%, even when other covariates in the models account for as much as 20% of the variation in that independent variable. For a binary risk factor present in 20% of children, the detectable adjusted odds-ratio under the stated assumptions would be 2.45. Hence, we anticipate having adequate statistical power to detect risk factors associated with moderate to strong incremental discriminative capacity.

Statistical analysis

Prior to performing statistical analyses, detailed statistical analysis plans will be developed and published online[39].

Our primary model development objective will seek to achieve case definition models that provide high discrimination capacity and acceptable calibration in external samples, using principled approaches to guard against overfitting biases. It is likely there will be ambiguity in TRD determination due to a range of potential thresholds for what is considered "recovery". Consequently, sensitivity analyses may be repeated with different variations of TRD definitions. These definitions will be defined prior to analysis by expert paediatricians. We will follow a three-staged approach to identifying candidate risk factors and selecting from among them. In the first stage, investigators will specify risk predictors that could be reliably and validly measured early in the hospitalisation in limited-resource settings and which are plausibly associated with TRD response status. In the second stage, we will develop and validate logistic regression models, using TRD response status as the dependent variable, and using elastic net regression and related approaches to principled variable selection. In the third stage, we will repeat the analysis incorporating more sophisticated diagnostic assessments, such as biomarkers and ultrasound, which are available in higher resource settings. Finally, after each clinical prediction model is developed, we will use resampling methods with 10-fold cross-validation to estimate model performance in terms of discriminative capacity, as measured by empirical AUROC, internally and externally.

Our secondary objective of establishing biomarker cut-offs for TRD will follow a similar receiver operating characteristic curve based approach, except with specific biomarker concentrations as the principle predictor. A weighted evaluation of each cut-offs sensitivity and specificity will be used in a 10-fold cross-validation framework to select the optimal cut-off[40].

The comparison of predictors, biomarkers, and risk factors across the community cohort, non-TRD hospital cohort, and TRD hospital cohort will be contextual and dependent on the type of variable considered. Analyses will be primarily descriptive, focused on substantive differences between cohorts, accompanied by visualisations and distribution appropriate statistical testing.

For peer review only - http://bmjopen.bmj.com/site/about/guidelines.xhtml

ETHICS AND DISSEMINATION

The study protocol was reviewed and approved by a steering committee at the Ministry of Health (MOH) and the Ministry of Foreign Affairs in Lao PDR. The study and consenting procedures were approved by the National Ethics Committee for Health Research, Ministry of Health, Lao PDR (ref. 11/2019) and the Institutional Review Board (IRB) of the UC Davis (ref. 1329444). The trial is registered at https://clinicaltrials.gov (NCT03626337). The executive leadership of LFHC also approved the conduct of the study as described here.

The presented protocol version (Version 2019-11-12) includes changes to the inclusion criteria, described in detail above. Any future modifications to the protocol including changes of study objectives, study design, inclusion criteria, sample sizes, study procedures, or significant administrative aspects will be agreed upon by the principal investigators and co-investigators, and approved by the National Ethics Committee for Health Research, Ministry of Health, Lao PDR and the UC Davis IRB prior to implementation. The present study will be implemented among children who will be admitted to the hospital for TDD like symptoms. Study participants will be treated by the hospital physicians following the usual hospital procedures, and the study team will not interfere with the administration of required treatments and interventions. The one study-specific intervention administered to all hospitalised study participants is thiamine. Thiamine is a water-soluble vitamin prescribed in case of beriberi or thiamine deficiency[10,22]. Documented side effects include: nausea, flushing sensation, restlessness, sweating and weakness, and there may be hypersensitivity reaction to intravenous injection or tenderness at the intramuscular injection site[41]. All children will be examined for these symptoms over the course of the study (**Table 1**), and any observed adverse events will be documented. Serious adverse events that are unexpected and/or probably related to the research procedures will be reported immediately to the UC Davis IRB and the National Ethics Committee for Health Research in Lao PDR.

A Data and Safety Monitoring Board (DSMB) will be established for the purpose of this project. Three independent paediatricians will monitor patient safety while the study is ongoing. Interim analyses will be completed only for safety considerations. Reports of the occurrence of potential adverse events will be submitted to and reviewed by the DSMB members. A new report will be shared with the DSMB committee each time a quarter of study participants are enroled. The principal investigators have the right to terminate

For peer review only - http://bmjopen.bmj.com/site/about/guidelines.xhtml

the study if the incidence of side effects and/or severity of adverse events outweigh the benefits of the study. Because thiamine administration is an established intervention with mild temporary side effects, it is expected that the study will continue until planned completion. Detailed stopping rules have not been designed at this time.

Study results will be reported following recommendations by the International Committee of Medical Journal Editors[42], and in accordance with the TRIPOD Statement and other relevant guidelines from the EQUATOR Network[43,44]. The knowledge and information gained from the present study will be promptly and broadly disseminated through peer-reviewed scientific open access articles, and through communications and presentations at conferences. Moreover, the present study is part of a larger multi-country effort to address critical questions related to thiamine deficiency and public health in Asia and Africa[45,46]. An important aspect of the overall project is to exchange research results among the collaborators. In-country result dissemination will be led by the Lao Tropical and Public Health Institute through meetings and workshops with representatives of the Ministry of Health, the Provincial Health Departments and other key stakeholders. Research briefs will also be produced for in-country distribution. In addition, the findings will be presented at the Annual Lao Pediatric Continuing Medical Education Conference organised by the Lao Pediatric Association and at the National Health Research Forum in Vientiane, Lao PDR.

TRIAL STATUS

The first participants of the hospital and community cohort were enroled in June 2019. Changes to the enrolment criteria were implemented in November 2019 (after 18.1% of the hospital and 13.6% of the community target sample size had been enroled). We anticipate data collection will be completed by December 2020.

ACKNOWLEDGEMENTS

For peer review only - http://bmjopen.bmj.com/site/about/guidelines.xhtml

For peer review only

We are grateful for the valuable input during the proposal development by Professor Paul Newton (Lao-Oxford-Mahosot Hospital-Wellcome Trust Research Unit, Vientiane, Lao PDR), Professor Kenneth Brown (Institute for Global Nutrition, UC Davis, Davis, CA, USA), and Dr. Sommanikhone Phangmanixay (Children Hospital, Vientiane, Lao PDR), and for Dr. Somphou Sayasone's (Lao Tropical and Public Health Institute, Vientiane, Lao PDR) administrative support throughout the study preparation and implementation. We appreciate that Dr. David Brown (Department of Cardiology, Boston Children's Hospital, Boston, MA, USA) and Kirk Stonick (Kaiser Permanente, Portland, OR, USA) traveled to Luang Prabang to conduct a one-week training in echocardiograms and cranial ultrasonography, respectively, and Dr. Kethmany Ratsavong's (Lao Tropical and Public Health Institute, Vientiane, Lao PDR) help with English to Lao translations during study staff trainings. We thank all physicians, nurses, laboratory technicians and administrative staff of the Lao Friends Hospital for Children (Luang Prabang, Lao PDR) for their collaboration, with special thanks to the Medical Director, Dr. Lisa Rynn. We thank Dr. Aviva Fattal-Valevski (Tel Aviv University, Tel Aviv, Israel) for providing input on the physical data collection form and the members of the DSMB (Dr. Charles Larson, School of Population and Global Health, McGill University, Montreal, Canada; Dr. Bandith Soumpholphakdy, Mahosot Hospital, Vientiane, Lao PDR; Dr. Daniel Roth, Pediatrician, The Hospital for Sick Children, Toronto, Canada) for their willingness to review data. Lastly, we are grateful to Medela AG (Baar, Switzerland) to lend hospital-grade breastmilk pumps for the duration of the study and to donate breastmilk collection supplies.

AUTHOR CONTRIBUTORS

SYH led the proposal writing. All authors made intellectual contributions to the study design or study protocol specific to their respective expertise. The manuscript was drafted by SYH, TJS and CDA. All coauthors reviewed the manuscript critically and approved the final content.

FUNDING

This work is supported by the Bill & Melinda Gates Foundation (grant number OPP1182864). Dr. Kenneth Brown provided technical support in the design of this study, when working for the Bill & Melinda Gates Foundation. The funding source will not have any role during its execution, analyses, interpretation of the data, or decision to submit results.

For peer review only - http://bmjopen.bmj.com/site/about/guidelines.xhtml

COMPETING INTERESTS

All authors have completed the ICMJE uniform disclosure form at www.icmje.org/coi_disclosure.pdf and declare: SYH has completed a consultancy for the Bill & Melinda Gates Foundation and her spouse previously worked for the Bill & Melinda Gates Foundation and; no other relationships or activities that could appear to have influenced the submitted work.

DATA SHARING STATEMENT

The complete de-identified dataset will be made publically available 3 years after completion of data collection on Open Science Framework at https://osf.io/jfke3.

REFERENCES

1. Johnson CR, Fischer PR, Thacher TD, et al. Thiamin deficiency in low- and middle-income countries: Disorders, prevalences, previous interventions and current recommendations. *Nutrition and Health* 2019;25:127-51.
2. Whitfield KC, Bourassa MW, Adamolekun B, et al. Thiamine deficiency disorders: diagnosis, prevalence, and a roadmap for global control programs. *Annals of the New York Academy of Sciences* 2018;1430:3-43.
3. Barennes H, Sengkhamyong K, Rene JP, et al. Beriberi (thiamine deficiency) and high infant mortality in northern Laos. *PLoS Negl Trop Dis* 2015;9:e0003581.
4. Khounnorath S, Chamberlain K, Taylor AM, et al. Clinically unapparent infantile thiamin deficiency in Vientiane, Laos. *PLoS Negl Trop Dis* 2011;5:e969.
5. Soukaloun D, Lee SJ, Chamberlain K, et al. Erythrocyte transketolase activity, markers of cardiac dysfunction and the diagnosis of infantile beriberi. *PLoS Negl Trop Dis* 2011;5:e971.
6. Barennes H, Simmala C, Odermatt P, et al. Postpartum traditions and nutrition practices among urban Lao women and their infants in Vientiane, Lao PDR. *European Journal of Clinical Nutrition* 2009;63:323-31.
7. Holmes W, Hoy D, Lockley A, et al. Influences on maternal and child nutrition in the highlands of the northern Lao PDR. *Asia Pacific Journal of Clinical Nutrition* 2007;16:537-45.
8. Soukaloun D, Kounnavong S, Phengdy B, et al. Dietary and socio-economic factors associated with beriberi in breastfed Lao infants. *Annals of Tropical Paediatrics* 2003;23:181-6.
9. Allen LH. B vitamins in breast milk: relative importance of maternal status and intake, and effects on infant status and function. *Adv Nutr* 2012;3:362-9.
10. World Health Organization. Thiamine deficiency and its prevention and control in major emergencies. Geneva: World Health Organization, 1999.
11. Hiffler L, Rakotoambinina B, Lafferty N, et al. Thiamine Deficiency in Tropical Pediatrics: New Insights into a Neglected but Vital Metabolic Challenge. *Front Nutr* 2016;3:16.

For peer review only - http://bmjopen.bmj.com/site/about/guidelines.xhtml

12. Wani NA, Qureshi UA, Ahmad K, et al. Cranial Ultrasonography in Infantile Encephalitic Beriberi: A Useful First-Line Imaging Tool for Screening and Diagnosis in Suspected Cases. *AJNR American Journal of Neuroradiology* 2016;37:1535-40.
13. Carreras I, Martinesz Garcia D, Indi EP, et al. Thiamine deficiency in neurological acute disorders in resource-limited settings: case reports. 3rd World Congress on Controversies in Pediatrics; 2016; Barcelona, Spain.
14. Duce M, Escriba J, Masuet C, et al. Suspected thiamine deficiency in Angola. *Field Exch* 2003;20:26-28.
15. Ogunlesi TA. Thiamine deficiency: a cause of childhood ataxia not to be ignored. *Annals of Tropical Paediatrics* 2004;24:357-60.
16. Keating EM, Nget P, Kea S, et al. Thiamine deficiency in tachypnoeic Cambodian infants. *Paediatr Int Child Health* 2015;35:312-8.
17. Coats D, Shelton-Dodge K, Ou K, et al. Thiamine deficiency in Cambodian infants with and without beriberi. *Journal of Pediatrics* 2012;161:843-7.
18. Lima LF, Leite HP, Taddei JA. Low blood thiamine concentrations in children upon admission to the intensive care unit: risk factors and prognostic significance. *American Journal of Clinical Nutrition* 2011;93:57-61.
19. Gibson RS. Principles of nutritional assessment. 2nd ed. New York: Oxford University Press 2005.
20. Talwar D, Davidson H, Cooney J, et al. Vitamin B(1) status assessed by direct measurement of thiamin pyrophosphate in erythrocytes or whole blood by HPLC: comparison with erythrocyte transketolase activation assay. *Clinical Chemistry* 2000;46:704-10.
21. Herve C, Beyne P, Letteron P, et al. Comparison of erythrocyte transketolase activity with thiamine and thiamine phosphate ester levels in chronic alcoholic patients. *Clinica Chimica Acta* 1995;234:91-100.
22. World Health Organization Western Pacific Region. Pocket book of hospital care for children. Guidelines for the management of common childhood illnesses. 4 ed. Manila, The Philippines: World Health Organization Western Pacific Region 2017.
23. Frank LL. Thiamin in Clinical Practice. *JPEN Journal of Parenteral and Enteral Nutrition* 2015;39:503-20.
24. Crook MA, Sriram K. Thiamine deficiency: the importance of recognition and prompt management. *Nutrition* 2014;30:953-4.
25. McCandless DW. Beriberi. Thiamine Deficiency and Associated Clinical Disorders Contemporary Clinical Neuroscience: Humana Press 2010.
26. Park SW, Yi YY, Han JW, et al. Wernicke's encephalopathy in a child with high dose thiamine therapy. *Korean J Pediatr* 2014;57:496-9.
27. WHO Multicentre Growth Reference Study Group. WHO Child Growth Standards: Length/height-for-age, weight-for-age, weight-for-length, weight-for-height and body mass index-for-age: Methods and development. Geneva: World Health Organization, 2006.
28. Hampel D, Shahab-Ferdows S, Islam MM, et al. Vitamin Concentrations in Human Milk Vary with Time within Feed, Circadian Rhythm, and Single-Dose Supplementation. *Journal of Nutrition* 2017;147:603-11.
29. Lu J, Frank EL. Rapid HPLC measurement of thiamine and its phosphate esters in whole blood. *Clinical Chemistry* 2008;54:901-6.
30. Hampel D, York ER, Allen LH. Ultra-performance liquid chromatography tandem mass-spectrometry (UPLC–MS/MS) for the rapid, simultaneous analysis of thiamin, riboflavin, flavin adenine dinucleotide, nicotinamide and pyridoxal in human milk. *Journal of Chromotography B* 2012;903:7-13.
31. Lopez L, Colan SD, Frommelt PC, et al. Recommendations for quantification methods during the performance of a pediatric echocardiogram: a report from the Pediatric Measurements Writing Group of the American Society of Echocardiography Pediatric and Congenital Heart Disease Council. *Journal of the American Society of Echocardiography* 2010;23:465-95; quiz 576-7.

For peer review only - http://bmjopen.bmj.com/site/about/guidelines.xhtml

32. Cashin K, Oot L. Guide to anthropometry: A practical tool for program planners, managers, and implementers. Washington DC: Food and Nutrition Technical Assistance III Project (FANTA)/ FHI 360, 2018.
33. de Onis M, Onyango AW, Van den Broeck J, et al. Measurement and standardization protocols for anthropometry used in the construction of a new international growth reference. *Food and Nutrition Bulletin* 2004;25:S27-36.
34. World Health Organization. Recommendations for data collection, analysis and reporting on anthropometric indicators in children under 5 years old. Geneva, Switzerland: World Health Organization and United Nations Children's Fund, 2019.
35. Wessells KR, Brown KH, Kounnavong S, et al. Comparison of two forms of daily preventive zinc supplementation versus therapeutic zinc supplementation for diarrhea on young children's physical growth and risk of infection: study design and rationale for a randomized controlled trial. *BMC Nutrition* 2018;4:39.
36. Coates J, Swindale A, Bilinsky P. Household Food Insecurity Access Scale (HFIAS) for measurement of food access: Indicator guide. In: FANTA, ed. Washington, DC: USAID, 2007.
37. Crowne DP, Marlowe D. A new scale of social desirability independent of psychopathology. *Journal of Consulting Psychology* 1960;24:349-54.
38. Menon P, Nguyen PH, Saha KK, et al. Combining Intensive Counseling by Frontline Workers with a Nationwide Mass Media Campaign Has Large Differential Impacts on Complementary Feeding Practices but Not on Child Growth: Results of a Cluster-Randomized Program Evaluation in Bangladesh. *Journal of Nutrition* 2016;146:2075-84.
39. Hess SY, Smith TJ, Arnold CD. Lao Thiamine Study: Open Science Framework; 2019 [Available from: https://osf.io/jfke3 accessed July 31 2019.
40. Perkins NJ, Schisterman EF. The inconsistency of "optimal" cutpoints obtained using two criteria based on the receiver operating characteristic curve. *American Journal of Epidemiology* 2006;163:670-5.
41. American Pharmacists Association. Lexicomp Drug Information Handbook: A Clinically Relevant Resource for All Healthcare Professionals 27 ed: Wolters Kluver 2018-2019.
42. International Committee of Medical Journal Editors. Recommendations for the conduct, reporting, editing, and publication of scholarly work in medical journals: ICMJE; 2019 [Available from: http://www.icmje.org/recommendations/ accessed November 19 2019.
43. Collins GS, Reitsma JB, Altman DG, et al. Transparent reporting of a multivariable prediction model for individual prognosis or diagnosis (TRIPOD): the TRIPOD statement. *BMJ* 2015;350:g7594.
44. Equator Network. Enhancing the quality and transparency of health research 2019 [cited 2019. Available from: http://www.equator-network.org/ accessed September 9 2019.
45. Whitfield KC, Kroeun H, Green T, et al. Thiamine dose response in human milk with supplementation among lactating women in Cambodia: study protocol for a double-blind, four-parallel arm randomised controlled trial. *BMJ Open* 2019;9:e029255.
46. Bergeron G, Bourassa M. Overcoming the current challenges of thiamine deficiency disorders *Current Developments in Nutrition* 2019;3:nzz034.P10-91-19.

For peer review only - http://bmjopen.bmj.com/site/about/guidelines.xhtml

Table 1: Timing of study procedures of hospitalised study children and their mothers

Activity	Prior to enrolment	Baseline	Medical treatment	Hours and weeks after thiamine administration										
				0-1 hr	4 hr	8 hr	12 hr	24 hr	36 hr	48 hr	72 hr	>72 hr[2]	2-4 wks[3]	6 wks[4]
Child's study participation and data collection														
Care seeking at hospital	X													
Parental consent for child's participation[1]	X													
Enrolment		X												
Venous blood sample		X												
Thiamine administration			X					X		X				
Other clinically indicated treatments			XXXXXXXXXXXXXXXXXXXXXXXXXXXXXXXXXXXXXXXXXXXXXXXXXXXXXXXXXXXXXXXXXXXX											
General physical exam		X			X	X	X	X	X	X	X	X	X	X
Liver palpation		X			X	X	X	X	X	X	X	X	X	X
Heart rate		X			X	X	X	X	X	X	X	X	X	X
Exam for oedema		X			X	X	X	X	X	X	X	X	X	X
Respiratory rate, oxygen saturation		X			X	X	X	X	X	X	X	X	X	X
Eye movement/contact assessment		X			X	X	X	X	X	X	X	X	X	X
Signs of ptosis and ophthalmoplegia		X			X	X	X	X	X	X	X	X	X	X
Hypotonia (head lag, floppy arms or legs in face-down position)		X			X	X	X	X	X	X	X	X	X	X
Consciousness evaluation		X			X	X	X	X	X	X	X	X	X	X
Vomiting frequency		X			X	X	X	X	X	X	X	X	X	X
Feeding behaviour		X			X	X	X	X	X	X	X	X	X	X
Anthropometry								X						
Echocardiogram		X						X		X			X	
Cranial ultrasound				X										X
Potential adverse events from thiamine injection			X	X	X	X	X	X	X	X	X	X		

For peer review only
For peer review only - http://bmjopen.bmj.com/site/about/guidelines.xhtml

Questionnaires														
Basic child characteristics		X												
Child’s birth and health history[5]					X									
SES characteristics and food security of child’s family[5]					X									
Dietary practices of child[5]					X									
Mother’s study participation and data collection														
Maternal consent	X[6]													
Maternal health[5]					X									
Maternal dietary practices[5]					X									
Maternal blood[7]					X									
Breast milk[7]					X									
Maternal anthropometry[7]										X				

[1] In case of life threatening condition, the child will be examined, blood will be drawn and all required medical interventions will be provided (including thiamine administration) prior to obtaining parental consent. Parental consent will be obtained as soon as the child’s condition has stabilised. Any data that has been collected will only be used, if parental written consent is obtained.

[2] Final physical exam prior to hospital discharge, timing will depend on hospital duration and time of discharge.

[3]Children with abnormal echocardiograms due to suspected thiamine deficiency will be invited for a follow up physical exam and echocardiogram after 2-4 weeks

[4] Children with abnormal cranial ultrasounds or neurological symptoms due to suspected thiamine deficiency will be invited for a follow up physical exam and cranial ultrasound after 6 weeks.

[5] Questionnaires will be administered during the hospital stay of the child. Timing will depend on hospital procedures and physical exams.

[6] Maternal consent will be obtained prior to enrolment into the study. The timing of obtaining maternal consent will depend on the health status of her child and hospital procedures.

[7] Maternal blood and breast milk will be collected as soon as possible after the mother’s written consent is obtained. Maternal anthropometry will be completed at a convenient time before the child’s discharge.

For peer review only

For peer review only - http://bmjopen.bmj.com/site/about/guidelines.xhtml

Figure 1: Study design overview of hospitalised and community cohort in the Lao Thiamine Study[1]

[1] At the onset of the study, the eligibility age was 21 days to <12 months. The target age was expanded to <18 months after 18.1% of hospital infants had been enroled, due to much lower enrolment rates than anticipated, and after consultation with expert paediatricians and the hospital leadership.

Figure 2: Flow chart of data collection timing among hospitalised infants and young children in the Lao Thiamine Study

For peer review only

For peer review only - http://bmjopen.bmj.com/site/about/guidelines.xhtml

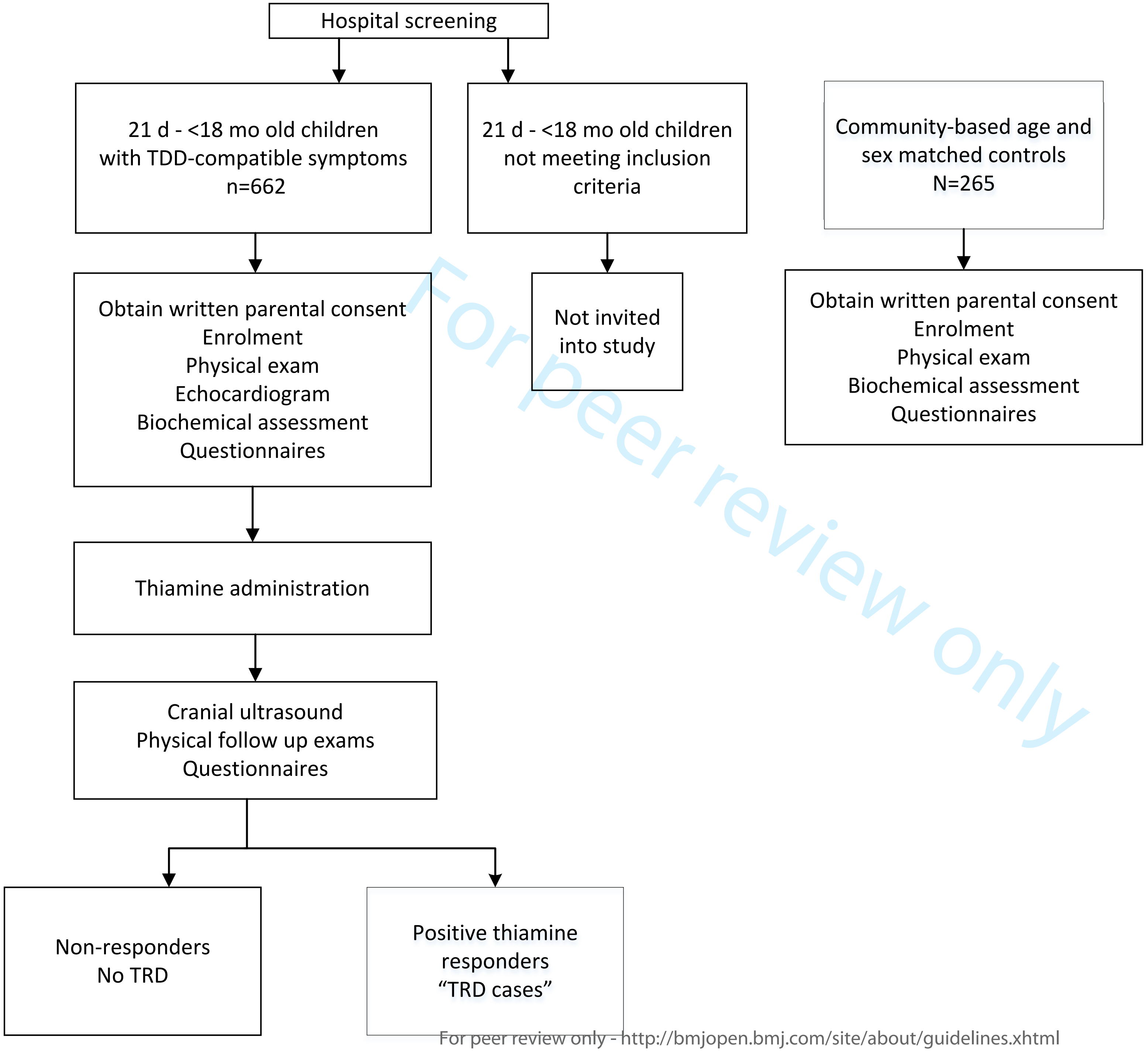

For peer review only

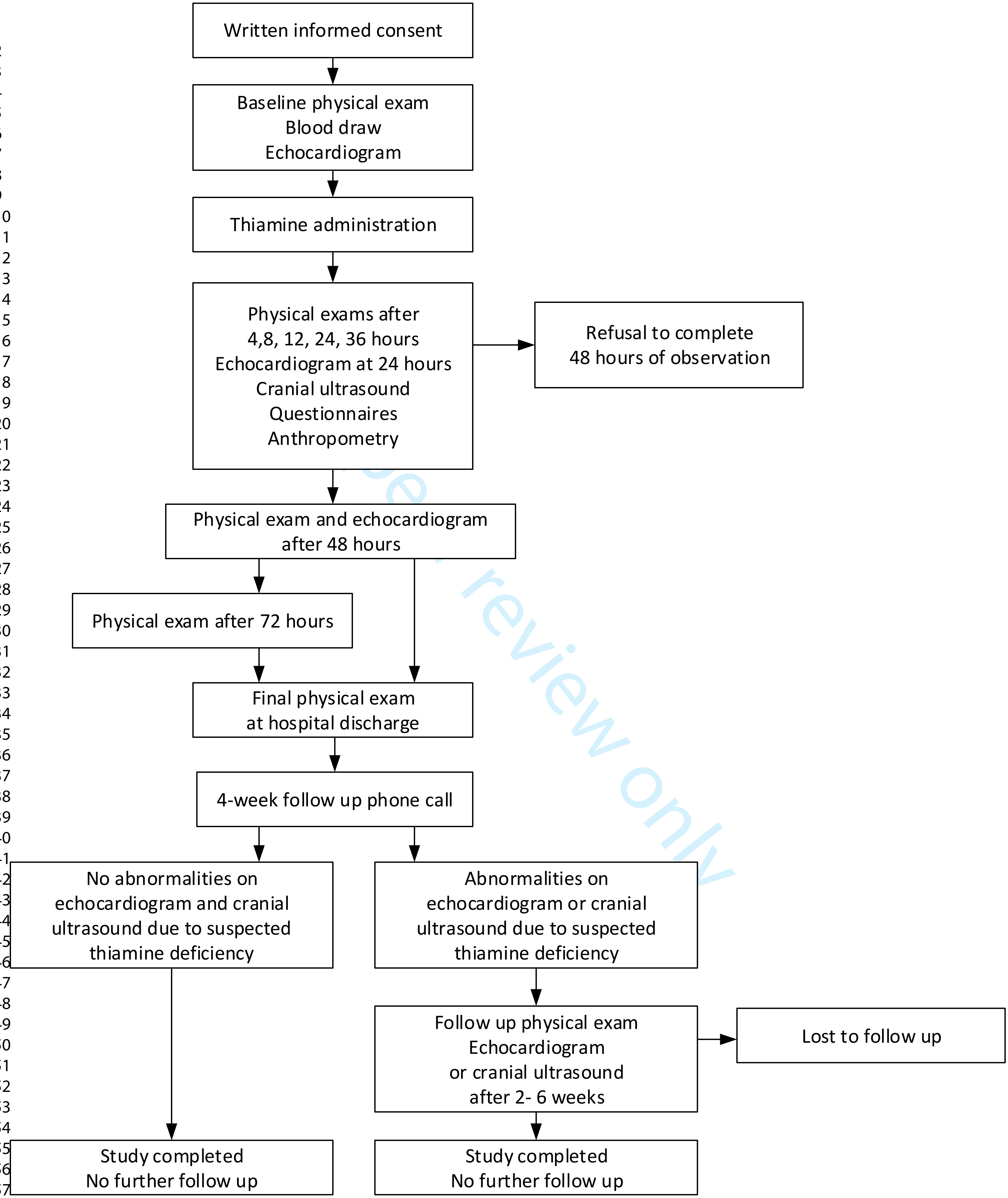
Written informed consent
Baseline physical exam
Blood draw
Echocardiogram
Thiamine administration
Physical exams after
4,8, 12, 24, 36 hours
Echocardiogram at 24 hours
Cranial ultrasound
Questionnaires
Anthropometry
Refusal to complete
48 hours of observation
Physical exam and echocardiogram
after 48 hours
Physical exam after 72 hours
Final physical exam
at hospital discharge
4-week follow up phone call
No abnormalities on
echocardiogram and cranial
ultrasound due to suspected
thiamine deficiency
Abnormalities on
echocardiogram or cranial
ultrasound due to suspected
thiamine deficiency
Follow up physical exam
Echocardiogram
or cranial ultrasound
after 2- 6 weeks
Lost to follow up
Study completed
No further follow up
Study completed
No further follow up

Supplementary Table 1: Inclusion criteria for study participants and outcomes of interest in the Lao Thiamine Study

**Inclusion criteria**	**Outcomes**		
	**Physical exams**	**Biochemical exams**	**Data collection via questionnaires**
Hospitalised children			
Target age range:<br>- 21 days to <18 months[1]<br><br>Plus at least one of the following criteria:<br>- Liver enlargement (>2cm below right costal margin, supine exam)<br>- OedemaTachypnea (> 60/min for 3-8 weeks; > 50/min for 2-11 moths; >40/min for 12 - 18 months)<br>- Tachycardia (heart rate >160/min for <12 months; >120/min for 12-18 months)<br>- Oxygen saturation < 92%<br>- Difficulty breathing (chest in-drawing or nasal flaring)<br>- Refusal to breastfeed or refusal of infant formula or food for greater than 24 hours<br>- Repetitive or recurrent vomiting with no obvious other cause (i.e. vomiting >3 times in past 24 hours)<br>- Persistent crying not relieved by soothing or feeding with no obvious other cause<br>- Hoarse voice/cry or loss of voice<br>- Nystagmus or other unusual eye movements<br>- Muscle twitching<br>- Loss of consciousness<br>- Convulsion<br>- Opisthotonus /abnormal posturing[2]<br>- Acute paralysis/flaccid paralysis[2]	- General physical exam performed by physician/nurse (calm, fussy, interactive; looks sick or well)<br>- Liver palpation<br>- Heart rate<br>- Exam for oedema (puffy eyelids, pitting oedema of back, genitals or ankles, whole body puffy)<br>- Respiratory distress (retractions, nasal flaring, paradoxical breathing, head bobbing)<br>- Respiratory rate, oxygen saturation, perfusion index<br>- Temperature<br>- Documentation of abnormal eye movements and eye contact<br>- Examine for signs of ptosis<br>- Hypotonia (head lag, floppy arms or legs in face-down position)<br>- Consciousness evaluation (AVPU scale)<br>- Documentation of timing of onset of each symptom<br>- Anthropometric indices (length, weight, mid-upper arm circumference, head circumference)<br>- Ultrasounds:<br>- Echocardiogram<br>- Cranial ultrasound<br><br>Follow up after thiamine administration:<br>- Repetition of physical exam after 4, 8 and 12 hours and every 12 hours for first 48 hours<br>- After 24 and 48 hrs: Echocardiogram<br>- After 72 hrs: Repetition of physical exam<br><br>For DSMB:	- ETKac in washed RBC<br>- Whole blood ThDP<br>- CBC<br>- Troponin T<br>- Lactate<br>- Indicators of inflammation (CRP, AGP)	- Basic demographics of child (age, sex, location of residence)<br>- Socioeconomic status of mother and family<br>- Gestational age at birth<br>- Birth/perinatal history<br>- Birth weight and length<br>- History of severe illnesses since birth and hospitalisations<br>- History of any illnesses in 2 weeks prior to onset of current illness<br>- History of bowel movements in past 24 hours (number and consistency), urinary outputs in past 24 hours and constipation in previous 7 days<br>- Recent and current intake of medications, dietary supplements and use of Tiger Balm<br>- Documentation of treatments and medications prescribed due to current illness, and thiamine at discharge<br>- Vaccination record<br>- Dietary practices of child (breastfeeding, complementary feeding)<br>- Maternal report on recent changes in physical movements, motor skills and behaviour

For peer review only

	- Response to thiamine injection/administration (such as local rash/redness, infection/swelling, temporary lethargy)		
Mothers of hospitalised children			
Exclude if:<br>- Severe acute illness warranting immediate hospital referral<br>- Unable to provide informed consent due to reduced decision making ability	- Maternal height, weight and mid-upper arm circumference	- ETKac in washed RBC<br>- Whole blood ThDP<br>- Indicators of inflammation (CRP, AGP)<br>- CBC<br>- Dried blood spots<br>- Breast milk thiamine concentration of lactating mothers	- Maternal characteristics (age, education, profession, marital status, ethnicity, religion, location of residence, family structure, number of children, etc.)<br>- Recent and current intake of multi-micronutrient supplements, medications and use of Tiger Balm<br>- Thiamine given at discharge<br>- Dietary practices of mother (dietary diversity questionnaire, food preparation, dietary beliefs/avoidance of certain foods, consumption of raw fish/fish paste, betel nut, tea and alcohol)<br>- Food security questionnaire<br>- History of beriberi (maternal beriberi and mother's children)<br>- Social desirability bias
Community-based age- and sex-matched children			
- Excluded if immediate hospital referral is required<br>- No other exclusion criteria	- General physical exam performed by study nurse<br>- Heart rate<br>- Respiratory rate, oxygen saturation, perfusion index<br>- Temperature<br>- Hypotonia (head lag, floppy arms or legs in face-down position)<br>- Anthropometric indices (length, weight, mid-upper arm circumference, head circumference)	- ETKac in washed RBC<br>- Whole blood ThDP<br>- CBC<br>- Indicators of inflammation (CRP, AGP)	- Basic demographics of child (age, sex, location of residence)<br>- Socioeconomic status of mother and family<br>- Gestational age at birth<br>- Birth/perinatal history<br>- Birth weight and length<br>- History of severe illnesses since birth and hospitalisations<br>- History of any illnesses in past 2 weeks<br>- History of bowel movements in past 24 hours (number and consistency), urinary outputs in past 24 hours and constipation in previous 7 days<br>- Recent and current intake of medications, dietary supplements and use of Tiger Balm<br>- Vaccination record<br>- Dietary practices of child (breastfeeding, complementary feeding)

For peer review only

			- Maternal report on recent changes in physical movements, motor skills and behaviour
Mothers of children in the community			
Exclude if:<br>- Severe acute illness warranting immediate hospital referral<br>- Unable to provide informed consent due to reduced decision making ability	- Maternal height and weight and mid-upper arm circumference	- ETKac in washed RBC<br>- Whole blood ThDP<br>- Indicators of inflammation (CRP, AGP)<br>- CBC<br>- Dried blood spots<br>- Breast milk thiamine concentration of lactating mothers	- Maternal characteristics (age, education, profession, marital status, ethnicity, religion, location of residence, family structure, number of children, etc.)<br>- Recent and current intake of multi-micronutrient supplements, medications and Tiger Balm<br>- Dietary practices of mother (dietary diversity questionnaire, food preparation, dietary beliefs/avoidance of certain foods, consumption of raw fish/fish paste, betel nut, tea and alcohol)<br>- Food security questionnaire<br>- History of beriberi (maternal beriberi and mother's children)<br>- Social desirability bias

AGP, α-1-acid glycoprotein; CBC, complete blood count; CRP, C-reactive protein; DSMB, data safety monitoring board; ETKac, erythrocyte transketolase activity coefficient; RBC, red blood cells; ThDP, thiamine diphosphate

[1] At the onset of the study, the eligibility age was 21 days to <12 months. The target age was expanded to <18 months after 18.1% hospital infants were enroled due to much lower enrolment rates than anticipated, and after consultation with expert paediatricians and the hospital leadership.
[2] Tachycardia, opisthotonus/abnormal posturing and acute paralysis/flaccid paralysis were added as inclusion criteria at the time when the target age range was extended to <18 months of age.

For peer review only

STANDARD PROTOCOL ITEMS: RECOMMENDATIONS FOR INTERVENTIONAL TRIALS

SPIRIT 2013 Checklist: Recommended items to address in a clinical trial protocol and related documents*

Section/item	ItemNo	Description	Page
**Administrative information**			
Title	1	Descriptive title identifying the study design, population, interventions, and, if applicable, trial acronym	1
Trial registration	2a	Trial identifier and registry name. If not yet registered, name of intended registry	20
	2b	All items from the World Health Organization Trial Registration Data Set	n/a
Protocol version	3	Date and version identifier	22
Funding	4	Sources and types of financial, material, and other support	23
Roles and responsibilities	5a	Names, affiliations, and roles of protocol contributors	22
	5b	Name and contact information for the trial sponsor	1
	5c	Role of study sponsor and funders, if any, in study design; collection, management, analysis, and interpretation of data; writing of the report; and the decision to submit the report for publication, including whether they will have ultimate authority over any of these activities	23
	5d	Composition, roles, and responsibilities of the coordinating centre, steering committee, endpoint adjudication committee, data management team, and other individuals or groups overseeing the trial, if applicable (see Item 21a for data monitoring committee)	n/a
**Introduction**			
Background and rationale	6a	Description of research question and justification for undertaking the trial, including summary of relevant studies (published and unpublished) examining benefits and harms for each intervention	6-8

For peer review only

	6b	Explanation for choice of comparators	5
Objectives	7	Specific objectives or hypotheses	8
Trial design	8	Description of trial design including type of trial (eg, parallel group, crossover, factorial, single group), allocation ratio, and framework (eg, superiority, equivalence, noninferiority, exploratory)	8
**Methods: Participants, interventions, and outcomes**			
Study setting	9	Description of study settings (eg, community clinic, academic hospital) and list of countries where data will be collected. Reference to where list of study sites can be obtained	9
Eligibility criteria	10	Inclusion and exclusion criteria for participants. If applicable, eligibility criteria for study centres and individuals who will perform the interventions (eg, surgeons, psychotherapists)	9-11
Interventions	11a	Interventions for each group with sufficient detail to allow replication, including how and when they will be administered	12
	11b	Criteria for discontinuing or modifying allocated interventions for a given trial participant (eg, drug dose change in response to harms, participant request, or improving/worsening disease)	n/a
	11c	Strategies to improve adherence to intervention protocols, and any procedures for monitoring adherence (eg, drug tablet return, laboratory tests)	n/a
	11d	Relevant concomitant care and interventions that are permitted or prohibited during the trial	13
Outcomes	12	Primary, secondary, and other outcomes, including the specific measurement variable (eg, systolic blood pressure), analysis metric (eg, change from baseline, final value, time to event), method of aggregation (eg, median, proportion), and time point for each outcome. Explanation of the clinical relevance of chosen efficacy and harm outcomes is strongly recommended	21-22
Participant timeline	13	Time schedule of enrolment, interventions (including any run-ins and washouts), assessments, and visits for participants. A schematic diagram is highly recommended (see Figure)	14 Figure 2

For peer review only - http://bmjopen.bmj.com/site/about/guidelines.xhtml

Sample size	14	Estimated number of participants needed to achieve study objectives and how it was determined, including clinical and statistical assumptions supporting any sample size calculations	20
Recruitment	15	Strategies for achieving adequate participant enrolment to reach target sample size	9-10
**Methods: Assignment of interventions (for controlled trials)**			
Allocation:			
Sequence generation	16a	Method of generating the allocation sequence (eg, computer-generated random numbers), and list of any factors for stratification. To reduce predictability of a random sequence, details of any planned restriction (eg, blocking) should be provided in a separate document that is unavailable to those who enrol participants or assign interventions	n/a
Allocation concealment mechanism	16b	Mechanism of implementing the allocation sequence (eg, central telephone; sequentially numbered, opaque, sealed envelopes), describing any steps to conceal the sequence until interventions are assigned	n/a
Implementation	16c	Who will generate the allocation sequence, who will enrol participants, and who will assign participants to interventions	n/a
Blinding (masking)	17a	Who will be blinded after assignment to interventions (eg, trial participants, care providers, outcome assessors, data analysts), and how	n/a
	17b	If blinded, circumstances under which unblinding is permissible, and procedure for revealing a participant's allocated intervention during the trial	n/a
**Methods: Data collection, management, and analysis**			
Data collection methods	18a	Plans for assessment and collection of outcome, baseline, and other trial data, including any related processes to promote data quality (eg, duplicate measurements, training of assessors) and a description of study instruments (eg, questionnaires, laboratory tests) along with their reliability and validity, if known. Reference to where data collection forms can be found, if not in the protocol	18

	18b	Plans to promote participant retention and complete follow-up, including list of any outcome data to be collected for participants who discontinue or deviate from intervention protocols	14
Data management	19	Plans for data entry, coding, security, and storage, including any related processes to promote data quality (eg, double data entry; range checks for data values). Reference to where details of data management procedures can be found, if not in the protocol	20
Statistical methods	20a	Statistical methods for analysing primary and secondary outcomes. Reference to where other details of the statistical analysis plan can be found, if not in the protocol	21-22
	20b	Methods for any additional analyses (eg, subgroup and adjusted analyses)	22
	20c	Definition of analysis population relating to protocol non-adherence (eg, as randomised analysis), and any statistical methods to handle missing data (eg, multiple imputation)	21-22
**Methods: Monitoring**			
Data monitoring	21a	Composition of data monitoring committee (DMC); summary of its role and reporting structure; statement of whether it is independent from the sponsor and competing interests; and reference to where further details about its charter can be found, if not in the protocol. Alternatively, an explanation of why a DMC is not needed	23
	21b	Description of any interim analyses and stopping guidelines, including who will have access to these interim results and make the final decision to terminate the trial	23
Harms	22	Plans for collecting, assessing, reporting, and managing solicited and spontaneously reported adverse events and other unintended effects of trial interventions or trial conduct	22
Auditing	23	Frequency and procedures for auditing trial conduct, if any, and whether the process will be independent from investigators and the sponsor	n/a
**Ethics and dissemination**			

For peer review only - http://bmjopen.bmj.com/site/about/guidelines.xhtml

Research ethics approval	24	Plans for seeking research ethics committee/institutional review board (REC/IRB) approval	22
Protocol amendments	25	Plans for communicating important protocol modifications (eg, changes to eligibility criteria, outcomes, analyses) to relevant parties (eg, investigators, REC/IRBs, trial participants, trial registries, journals, regulators)	22
Consent or assent	26a	Who will obtain informed consent or assent from potential trial participants or authorised surrogates, and how (see Item 32)	11-12
	26b	Additional consent provisions for collection and use of participant data and biological specimens in ancillary studies, if applicable	10
Confidentiality	27	How personal information about potential and enrolled participants will be collected, shared, and maintained in order to protect confidentiality before, during, and after the trial	
Declaration of interests	28	Financial and other competing interests for principal investigators for the overall trial and each study site	23
Access to data	29	Statement of who will have access to the final trial dataset, and disclosure of contractual agreements that limit such access for investigators	18
Ancillary and post-trial care	30	Provisions, if any, for ancillary and post-trial care, and for compensation to those who suffer harm from trial participation	n/a
Dissemination policy	31a	Plans for investigators and sponsor to communicate trial results to participants, healthcare professionals, the public, and other relevant groups (eg, via publication, reporting in results databases, or other data sharing arrangements), including any publication restrictions	21
	31b	Authorship eligibility guidelines and any intended use of professional writers	21
	31c	Plans, if any, for granting public access to the full protocol, participant-level dataset, and statistical code	23

Appendices

For peer review only

Informed consent materials	32	Model consent form and other related documentation given to participants and authorised surrogates	osf
Biological specimens	33	Plans for collection, laboratory evaluation, and storage of biological specimens for genetic or molecular analysis in the current trial and for future use in ancillary studies, if applicable	n/a

*It is strongly recommended that this checklist be read in conjunction with the SPIRIT 2013 Explanation & Elaboration for important clarification on the items. Amendments to the protocol should be tracked and dated. The SPIRIT checklist is copyrighted by the SPIRIT Group under the Creative Commons "Attribution-NonCommercial-NoDerivs 3.0 Unported" license.

For peer review only

BMJ Open

Establishing a case definition of thiamine responsive disorders among infants and young children in Lao PDR: Protocol for a prospective cohort study

Journal:	*BMJ Open*
Manuscript ID	bmjopen-2019-036539.R1
Article Type:	Protocol
Date Submitted by the Author:	11-Jan-2020
Complete List of Authors:	Hess, Sonja; University of California Davis, Institute for Global Nutrition<br>Smith, Taryn; University of California Davis, Institute for Global Nutrition<br>Fischer, Philip; Mayo, Pediatric and Adolescent Medicine<br>Trehan, Indi; University of Washington, Department of Pediatrics and Department of Global Health; Lao Friends Hospital for Children<br>Hiffler, Laurent ; Independent Consultant Pediatrician<br>Arnold, Charles D; University of California Davis, Institute for Global Nutrition<br>Sitthideth, Dalaphone; Lao Tropical and Public Health Institute<br>Tancredi, Daniel; University of California Davis Health System, Department of Pediatrics<br>Schick, Michael; University of California Davis Health System, Emergency Medicine<br>Yeh, Jay; University of California Davis Health System, Department of Pediatrics, Division of Cardiology<br>Stein-Wexler, Rebecca; University of California Davis Health System, Department of Radiology<br>McBeth, Christine; University of California Davis Health System, Emergency Medicine<br>Tan, Xiuping; University of California Davis, Institute for Global Nutrition<br>Nhiacha, Kouyang; Lao-Korea Children Hospital<br>Kounnavong, Sengchanh; Lao Tropical and Public Health Institute
**Primary Subject Heading**:	Global health
Secondary Subject Heading:	Nutrition and metabolism, Cardiovascular medicine, Paediatrics, Radiology and imaging
Keywords:	NUTRITION & DIETETICS, Echocardiography < CARDIOLOGY, Neuroradiology < NEUROLOGY, PAEDIATRICS

SCHOLARONE™
Manuscripts

I, the Submitting Author has the right to grant and does grant on behalf of all authors of the Work (as defined in the below author licence), an exclusive licence and/or a non-exclusive licence for contributions from authors who are: i) UK Crown employees; ii) where BMJ has agreed a CC-BY licence shall apply, and/or iii) in accordance with the terms applicable for US Federal Government officers or employees acting as part of their official duties; on a worldwide, perpetual, irrevocable, royalty-free basis to BMJ Publishing Group Ltd ("BMJ") its licensees and where the relevant Journal is co-owned by BMJ to the co-owners of the Journal, to publish the Work in this journal and any other BMJ products and to exploit all rights, as set out in our licence.

The Submitting Author accepts and understands that any supply made under these terms is made by BMJ to the Submitting Author unless you are acting as an employee on behalf of your employer or a postgraduate student of an affiliated institution which is paying any applicable article publishing charge ("APC") for Open Access articles. Where the Submitting Author wishes to make the Work available on an Open Access basis (and intends to pay the relevant APC), the terms of reuse of such Open Access shall be governed by a Creative Commons licence – details of these licences and which Creative Commons licence will apply to this Work are set out in our licence referred to above.

Other than as permitted in any relevant BMJ Author's Self Archiving Policies, I confirm this Work has not been accepted for publication elsewhere, is not being considered for publication elsewhere and does not duplicate material already published. I confirm all authors consent to publication of this Work and authorise the granting of this licence.

For peer review only

Establishing a case definition of thiamine responsive disorders among infants and young children in Lao PDR: Protocol for a prospective cohort study

Sonja Y Hess[1], Taryn J Smith[1], Philip R Fischer[2], Indi Trehan[3,4], Laurent Hiffler[5], Charles D Arnold[1], Dalaphone Sitthideth[6], Daniel J Tancredi[7], Michael A Schick[8], Jay Yeh[9], Rebecca Stein-Wexler[10], Christine N McBeth[8], Xiuping Tan[1], Kouyang Nhiacha[11], Sengchanh Kounnavong[6]

[1] Institute for Global Nutrition, University of California Davis, Davis, CA, USA
[2] Department of Pediatric and Adolescent Medicine, Mayo Clinic, Rochester, MN, USA
[3] Lao Friends Hospital for Children, Luang Prabang, Lao PDR
[4] Department of Pediatrics and Department of Global Health, University of Washington, Seattle, WA, USA
[5] Independent Consultant Pediatrician, Lagny sur Marne, France
[6] Lao Tropical and Public Health Institute, Ministry of Health, Vientiane, Lao PDR
[7] Department of Pediatrics, University of California Davis Health, Sacramento, CA, USA
[8] Emergency Medicine, University of California Davis Health, Sacramento, CA, USA
[9] Department of Pediatrics, Division of Cardiology, University of California Davis Health, Sacramento, CA, USA
[10] Department of Radiology, University of California Davis Health, Sacramento, CA, USA
[11] Lao-Korean Children Hospital, Vientiane, Lao PDR

Correspondence to: Sonja Y. Hess, Institute for Global Nutrition, University of California Davis, One Shields Avenue, Davis, CA 95616, USA. Email: syhess@ucdavis.edu Phone: 530 752 1992

Co-author contact information:
Sonja Y Hess: syhess@ucdavis.edu
Taryn J Smith: tjsm@ucdavis.edu
Philip R Fischer: Fischer.Phil@mayo.edu
Indi Trehan: indi@alum.berkeley.edu
Laurent Hiffler: Laurenthiffler@gmail.com
Charles D Arnold: cdarnold@ucdavis.edu
Dalaphone Sitthideth: dsitthideth@yahoo.com
Daniel J Tancredi: djtancredi@ucdavis.edu
Michael A Schick: mschick@ucdavis.edu
Jay Yeh: jayyeh@ucdavis.edu
Rebecca Stein-Wexler: rsteinwexler@ucdavis.edu

For peer review only - http://bmjopen.bmj.com/site/about/guidelines.xhtml

Christine N McBeth: cmcbeth@UCDAVIS.EDU
Xiuping Tan: xptan@ucdavis.edu
Kouyang Nhiacha: nhiacha.kouyang@gmail.com
Sengchanh Kounnavong: sengchanhkounnavong@hotmail.com

Word count: 7073

Word limit: 4000

Key words: Thiamine deficiency, beriberi, infant, children, Lao PDR

For peer review only

For peer review only - http://bmjopen.bmj.com/site/about/guidelines.xhtml

ABSTRACT

Introduction Diagnosis of infantile thiamine deficiency disorders (TDD) is challenging due to the non-specific, highly variable clinical presentation, often leading to misdiagnosis. Our primary objective is to develop a case definition for thiamine responsive disorders (TRD) to determine among hospitalised infants and young children, which clinical features and risk factors identify those who respond positively to thiamine administration.

Methods and analysis This prospective study will enrol 662 children (aged 21 days - <18 months) seeking treatment for TDD symptoms. Children will be treated with intravenous or intramuscular thiamine (100mg daily for a minimum of 3 days) alongside other interventions deemed appropriate. Baseline assessments, prior to thiamine administration, include a physical exam, echocardiogram and venous blood draw for determination of thiamine biomarkers. Follow up assessments include physical exams (after 4, 8, 12, 24, 36, 48 and 72 hours), echocardiogram (after 24 and 48 hours) and one cranial ultrasound. During the hospital stay, maternal blood and breastmilk samples and diet, health, anthropometric and sociodemographic information will be collected for mother-child pairs. Using this data, a panel of expert paediatricians will determine TRD status for use as the dependent variable in logistic regression models. Models identifying predictors of TRD will be developed and validated for various scenarios. Clinical prediction model performance will be quantified by empirical area under the receiver operating characteristic curve, using resampling cross-validation. A frequency matched community-based cohort of mother-child pairs (n = 265) will serve as comparison group for evaluation of potential risk factors for TRD.

Ethics and dissemination Ethical approval has been obtained from The National Ethics Committee for Health Research, Ministry of Health, Lao PDR and the Institutional Review Board of the University of California Davis. Results will be disseminated via scientific articles, presentations and workshops with representatives of the Ministry of Health.

Trial registration number NCT03626337.

For peer review only

For peer review only - http://bmjopen.bmj.com/site/about/guidelines.xhtml

ARTICLE SUMMARY

STRENGTHS AND LIMITATIONS OF THIS STUDY

- A standardised case definition for thiamine responsive disorder (TRD) will be proposed, through understanding of the interplay between clinical presentation, response to therapeutic thiamine administration and biochemical analyses, which will help more accurately diagnose infantile TRD.
- The broad spectrum of eligibility criteria will ensure inclusion of hospitalised infants and young children who may have milder, underlying thiamine deficiency and may respond clinically and benefit from thiamine administration, as well as those with suspected beriberi.
- Associations between TRD and biomarkers of thiamine status will help establish deficiency cut-off thresholds for thiamine diphosphate (ThDP) and erythrocyte transketolase activity coefficient (ETKac) that are relevant to a population with thiamine deficiency.
- Due to ethical considerations, the study does not include a placebo control group and will rely on a community comparison group to cross-check risk factors identified in the hospitalised cohort to a healthier population.
- In addition to the thiamine administration, hospitalised children will continue to receive routine care, such as antibiotics and other medications for concurrent illnesses that may affect the children's recovery, making the clear distinction between TRD cases and non-responders more challenging.

For peer review only - http://bmjopen.bmj.com/site/about/guidelines.xhtml

INTRODUCTION

Thiamine (vitamin B1) is an essential micronutrient, required as a cofactor for several enzymes involved in carbohydrate metabolism and energy production. Thiamine deficiency has been described in several low and middle income countries and remains a public health concern in many countries of Southeast Asia[1,2] , including Lao People's Democratic Republic (Lao PDR)[3-5]. Diets that lack in dietary diversity and rely too heavily on low-thiamine staple crops (such as polished white rice), food preparation practices (repetitive washing and soaking rice for many hours) and traditional postpartum restrictive diets, all place lactating women at high risk of thiamine deficiency in Lao PDR[6-8]. Breastmilk thiamine concentrations and infant thiamine status are strongly dependent on maternal thiamine intake and status, and maternal thiamine deficiency rapidly results in low breastmilk thiamine concentrations[9]. Infants are therefore especially vulnerable to thiamine deficiency in the first months of life, and exclusively breastfed infants of thiamine-deficient mothers are at the highest risk. According to the World Health Organization (WHO), infantile thiamine deficiency is the only serious form of malnutrition that occurs in adequately breastfed infants and only when the mother herself is thiamine deficient[10].

Thiamine deficiency presents as a broad spectrum of non-specific clinical abnormalities, referred to as thiamine deficiency disorders (TDD), of which the classic forms of beriberi in adults and children are best known, although even classic beriberi often goes unrecognised[11]. TDD can be characterised to some degree by age and features of clinical presentation[2]. Infantile TDD commonly occurs between 1 and 9 months of age, with non-specific early signs such as refusal to breastfeed, vomiting and irritability (1-3 months of age), which can progress to oedema, respiratory distress, heart failure and death if not rapidly treated. During later infancy, the cry may sound hoarse or change to a soundless cry (4-6 months of age) and neurological symptoms may present, including nystagmus, convulsions and unconsciousness (7-9 months of age). Infants with encephalitic beriberi were found to have abnormal cranial ultrasonography, with normalisation within 8 weeks of thiamine administration[12]. In the second year of life and later in childhood, TDD more typically presents with a broad range of neurological symptoms, such as anorexia, irritability, agitation, muscle pain, reduced deep tendon reflexes, ataxia, paralysis and altered levels of consciousness [11,13-15]. Due to uncertainties regarding the diagnostic criteria and overlapping symptoms with other conditions, TDD may frequently be misdiagnosed as bronchiolitis, pneumonia, typhoid fever, sepsis, meningitis or encephalitis, and the underlying thiamine deficiency may remain untreated[1,2,11]. Cases of TDD are often suspected rather than confirmed, and a final diagnosis is usually based upon a positive response to intramuscular or intravenous thiamine administration.

For peer review only - http://bmjopen.bmj.com/site/about/guidelines.xhtml

Diagnosis of TDD is further complicated by the fact that several studies have found little association between biomarkers of thiamine status and TDD-like symptoms[16-18]. It has been suggested that low thiamine status alone cannot reliably discriminate between symptomatic and asymptomatic infants, and infectious co-pathologies and other physiological stressors may precipitate the onset of TDD[17]. Conversely, low thiamine status appears to increase vulnerability to disease, as indicated by the observation that hospitalised infants with low thiamine status, but no clinical signs of beriberi, showed higher mortality rates in a region of Lao PDR[4].

Thiamine status can be determined by assessing thiamine metabolites in blood or urine. The most widely used biochemical indicator of thiamine status is the activity of the thiamine dependent transketolase enzyme in erythrocytes (ETK), which reflects the biological functionality of the vitamin and is sensitive to marginal thiamine deficiency[19]. Thiamine diphosphate (ThDP) concentration in whole blood or erythrocytes is also a useful indicator as ThDP is the biologically active vitamer and is reflective of body stores. There are however many challenges to assessing biomarkers of thiamine status, especially in resource-limited settings, including cost, the required cold chain and limited availability of laboratories able to analyse biological specimens. Hence, thiamine biomarkers are not routinely assessed and a rapid positive response to therapeutic thiamine is considered confirmation of a deficient status. Furthermore, there are currently no universally agreed upon adequacy or deficiency cut-off thresholds for erythrocyte or whole blood ThDP or ETK activity coefficient (ETKac), which have largely been studied in thiamine adequate adults in high-income countries. Consequently a wide range of cut-offs has been used[1]. Limited data derived from two studies in European adults at risk of thiamine deficiency from either chronic alcoholism or from an acute or chronic medical condition are available on associations between ETKac and ThDP[20,21]. These studies suggest that elevated ETKac (≥ 25%) occurs when ThDP concentrations are below 280 mg/g haemoglobin. However, it is not clear if similar results would be apparent in other populations. Thus, there is a need to develop clinically meaningful cut-off thresholds for biomarkers indicative of thiamine deficiency in other at-risk populations, especially infants and young children in countries where thiamine intake is inadequate and exclusive breastfeeding prevalent.

In summary, owing to the wide variability in clinical presentation, a barrier for reliable diagnosis and targeted treatment of TDD is the lack of a clear clinical case definition with broad consensus. There is a need to understand the interaction between thiamine status, exposure to risk factors (including infections) and onset of clinical symptoms in areas where thiamine deficiency is prevalent. Thus, this study aims to develop a

For peer review only - http://bmjopen.bmj.com/site/about/guidelines.xhtml

standardised case definition for thiamine responsive disorders (TRD), based on a combination of presenting clinical features most predictive of responding to therapeutic thiamine doses, alongside assessment of biochemical indicators of thiamine status and potential risk factors, in infants and young children aged 21 days to <18 months in Lao PDR.

OBJECTIVES OF STUDY

The study's primary objective is the development of a case definition for TRD among infants and young children with symptoms consistent with TDD, with the case definition being based on those clinical symptoms and other predictors that we find are better able to distinguish those who respond positively to thiamine administration from those who do not respond. As diagnostic tools available to treating physicians may differ by setting, we will repeat analyses after excluding from the set of candidate predictors those that are derived from assessments only available in higher-resource settings, such as laboratory biomarkers and ultrasonography.

A secondary objective is to fill the knowledge gap surrounding biomarkers of thiamine status in at-risk populations. Biomarker cut-offs for TRD will be developed in the hospital cohort and distributions will be characterised in both the hospital and community cohorts. The performance of our proposed cut-off will be compared to the performance of existing literature cut-offs.

Additionally, all identified predictors and biomarker cut-offs will be compared across the community cohort, non-TRD hospital cohort, and TRD hospital cohort to assess the prevalence of risk factors among apparently healthy infants and young children and assess the usefulness of the TRD case definition in various settings.

METHODS AND ANALYSIS

Study design and procedures

Study design

For peer review only - http://bmjopen.bmj.com/site/about/guidelines.xhtml

The study uses a prospective study design and includes a cohort of infants and young children 21 days to <18 months of age who are identified as having TDD-compatible symptoms (**Figure 1**). Children will be recruited at the Lao Friends Hospital for Children (LFHC) in Luang Prabang, Lao PDR, and potentially eligible children presenting with any of the below described inclusion criteria will be identified through close collaboration between the LFHC hospital staff and the study team. Enroled children will be treated with thiamine and all other clinically indicated treatments and followed closely over 48 – 72 hours, as described in more detail below. Based on the response to thiamine, children will either be defined as TRD cases or non-responders.

A community-based cohort of children frequency matched by age, sex and village of residence will be included in the study to serve as a comparison group for evaluation of potential predictors of TRD and thiamine deficiency in a non-hospitalised cohort. We will aim to achieve a comparison group that has a similar distribution of age, sex and location of residence as the hospitalised children, to minimise confounding biases. To achieve a well-balanced match, we will summarise the characteristics of the hospitalised children and enrol frequency matched community-based children weekly, to avoid seasonal discordance. Prior to visiting the health district, the head of the health centre and the head of the village will be contacted to ask for assistance in identifying a child of the right target age (within a 2-week period) and living in a selected village or, if not available, a neighbouring village. The health centre staff will establish contact with the child's parents/caregivers, briefly explain the study and invite the parents/caregivers for a study appointment at the health centre. All mothers of participating children in the hospital and community will also be invited into the study to obtain in-depth information on potential risk factors, including maternal thiamine status and thiamine concentration in breastmilk.

Inclusion and exclusion criteria

Inclusion and exclusion criteria of children at the hospital

Initially, infants 21 days to <12 months of age were the target population for the present study. This target age range was chosen because the risk of beriberi is highest during infancy, with highest mortality rates between 2-4 months of age[10]. Very young infants from 0 – 20 days of age are not eligible, because beriberi is rarely reported in the first few weeks of life and we did not want to include very young infants at greater risk of other serious illnesses, such as newborn sepsis, which could confound the study findings. The target age range was expanded to <18 months of age after 18.1% of the target hospital sample size had been enroled. This extension

For peer review only - http://bmjopen.bmj.com/site/about/guidelines.xhtml

For peer review only

of eligibility criteria was implemented due to much lower enrolment rates than anticipated in the first few months of the study, after further review of the scientific literature, and in consultation with expert paediatricians and the hospital leadership. Thus, throughout the majority of the study duration, the target age is 21 days to <18 months.

Children in the target age range, who are admitted to inpatient care at LFHC, will be screened by a study physician and/or study nurse to determine the presence of at least one of the following inclusion criteria:

- Liver enlargement (>2cm below right costal margin, supine exam)
- Oedema
- Tachypnea (respiratory rate > 60/min for 3-8 weeks; > 50/min for 2-11 months; >40/min for 12 – 18 months)
- Tachycardia (heart rate >160/min for <12 months; >120/min for 12-18 months)
- Oxygen saturation < 92%
- Difficulty breathing (chest in-drawing, or nasal flaring)
- Refusal to breastfeed or refusal of infant formula or food for greater than 24 hours
- Repetitive or recurrent vomiting with no obvious other cause (i.e. vomiting >3 times in past 24 hours)
- Persistent crying not relieved by soothing or feeding with no obvious other cause
- Hoarse voice/cry or loss of voice
- Nystagmus or other unusual eye movements
- Muscle twitching
- Loss of consciousness
- Convulsion
- Opisthotonus/abnormal posturing
- Acute paralysis/flaccid paralysis

The list of inclusion criteria has been developed based on TDD-compatible signs and symptoms in the broadest sense, in an effort to reduce the risk of potentially omitting children who would respond clinically to thiamine administration to correct any deficiencies. Tachycardia and two other inclusion criteria (opisthotonus/abnormal posturing and acute paralysis/flaccid paralysis) were added once the target age range was extended to <18 months of age to capture the full range of neurological signs and symptoms that might occur in the second year of life.

For peer review only - http://bmjopen.bmj.com/site/about/guidelines.xhtml

Inclusion and exclusion criteria of children in the community

The eligibility age for the community children is identical to that of the hospital. Thus, initially the target age range was 21 days to <12 months, and this was expanded to 21 days to <18 months after 13.6% of the community sample size had been enroled. Other inclusion criteria for the community cohort are that community children match the characteristics of the hospitalised children with regard to age, sex and community of residence characteristics. Children are not excluded based on any ineligibility criteria, except in case of severe acute illness warranting immediate hospital referral.

Inclusion and exclusion criteria of study participants' mothers

Once the hospitalised and community-based children are enroled, their mothers will also be invited for study participation, unless the women meet one of the following exclusion criteria: 1) severe acute illness warranting immediate hospital referral; or 2) unable to provide informed consent due to reduced decision-making ability.

Informed consent

Informed consent will be obtained from at least one primary caregiver (parent or legal guardian) of children for the child's study participation as well as the mothers (or other female primary caregiver) for their own study participation. Because the study activities vary depending on the study participant (child *vs*. mother) and the location (hospital *vs*. community), different consent forms will be used. If the parents and/or mother cannot read, an impartial witness will be present during the entire consent discussion to attest that the information in the consent form was accurately explained. If the parents do not understand Lao, but speak Hmong, a Hmong speaking study team member will lead the consenting procedure. In case of other languages, an individual will be identified to help translate all information into the appropriate language. The consent form will be read with the parents/mother and all information will be provided ensuring that the parents/mother understand what it will be like for their child or the mother, respectively, to participate in the study, and that the choice to participate will not affect the medical care that the child will receive while in the hospital or at any health centre. If the primary caregivers decide for the child to participate, one parent, either the mother, father or a legal guardian, or both, will be asked to sign or fingerprint the consent form of the child. The mother will be asked to sign or fingerprint her own consent form to confirm voluntary study participation. An additional

For peer review only - http://bmjopen.bmj.com/site/about/guidelines.xhtml

signature or fingerprint will be asked to confirm voluntary consent to storage and future analyses of biological specimen.

Once a child is identified as potentially eligible during the initial physical exam by the hospital medical team, a brief summary of the research study will be provided to explore the interest in study participation. In case the parent expresses an initial interest, and the child's health is stable, a study team member will give a copy of the approved consent form translated into Lao to the parent and the above described consenting procedure will be implemented. The same consenting procedure will be followed, but delayed, if the child's condition is critical or life threatening. In case the child's health does not allow postponing any exam or treatment, the child will be examined by a study physician and/or nurse and all health information will be recorded. A venous blood sample will be drawn and treatments (including intravenous or intramuscular thiamine) will be initiated prior to obtaining parental consent. Parental consent will be obtained as soon as the child's condition has stabilised, following the above described procedures. Any data and blood specimen that were collected prior to consent will only be used if parental written consent is obtained.

Intervention

Children in the hospital cohort will be treated by LFHC staff following standard care practices at the discretion of the primary medical team. The only intervention provided in the present study is thiamine. There are currently no evidence-based recommendations for thiamine dosage to treat infants and young children with severe acute illness[11]. Treatment guidelines in the pocket book of the WHO Western Pacific Region recommend treating an infant with heart failure with 25 mg thiamine intravenously and 25 mg intramuscularly, then 25 mg thiamine daily until the infant can eat, followed by 10 mg oral thiamine supplementation per day for 2 – 3 weeks[22]. Considering that previous studies used dosages ranging from 50 mg to 1500 mg depending on the clinical condition[17,23-26], and neurological presentations of TDD tend to require higher doses and longer recovery time[11], a daily dose of 25 - 50 mg was considered potentially insufficient. Consequently, daily doses of 100 mg thiamine, the standard treatment procedure of infants presenting with TDD at the LFHC, was deemed most appropriate for the present study. All children in the hospital cohort will be treated with a daily dose of 100 mg thiamine by intramuscular or intravenous administration for a minimum of three days. In general, intravenous dosing will be preferred, unless intravenous access cannot be obtained after several

For peer review only - http://bmjopen.bmj.com/site/about/guidelines.xhtml

attempts by LFHC nurses. Vials containing 100 mg thiamine as thiamine hydrochloride will be obtained locally (CBF Pharmaceutical Factory, Ban Kang, Pakse District, Lao PDR).

Procedures

Procedures at the hospital

Once the LFHC medical team have assessed the health of a potentially eligible patient, the study physician and/or nurse will complete the first standardised physical exam (i.e. baseline health status) by following a structured data collection form. The physical exam includes questions for caregivers on the child's recent health history, assessment of the child's general appearance, presence of cyanosis, breathing difficulties, bulging fontanelle, hypotonia, unusual eye movements, ptosis, sound of the child's voice/cry, muscle twitching, level of consciousness (assessed by the "alert, response to voice, response to pain, unresponsive" (AVPU) scale[22]), liver palpation and oedema. Vital signs, such as body temperature (Thermo Buddy Infrared Ear Thermometer TB-100, HuBDIC Co. Ltd., Anyang-si, Gyeonggi-do, Korea) and oxygen saturation of arterial haemoglobin, pulse rate, perfusion index, and respiration rate from pulse oximeter plethysmogram analysis will be measured using a Rad-G™ Pulse Oximeter (Masimo, Irvine, CA, USA) (Supplementary Table 1). A venous blood sample will be drawn and an echocardiogram will be performed, as described in more detail below. These data collection activities are performed prior to the first thiamine administration, unless the child's condition is critical, in which case the echocardiogram may be delayed until after the LFHC medical staff have initiated the thiamine administration and any appropriate treatments and the child's health is more stable. The date and time of each activity will be recorded for later consideration during statistical analyses. A cranial ultrasound will be performed during the hospital stay, typically within the first 12 hours.

The data collection will follow a structured timeline after the first thiamine dose has been administered (**Table 1**). In particular, the thiamine administration will be defined as hour zero, and the above described physical exam will be repeated 4, 8, 12, 24, 36, 48, and 72 hours after the initial thiamine administration (**Figure 2**). At each follow up time point, recovery status will also be assessed. Namely, at 4, 8, 12, 24, 36, 48, and 72 hours the child's health status will be described by the study physicians/nurses using a 5-point scale (seems worse; no change; seems a little bit better; seems a lot better; seems recovered). In addition, during the follow up exam after 24, 36, 48 and 72 hours, the study physicians will consult with the LFHC treating physicians whether the child is well enough to be discharged from the hospital. If a child's health has stabilised before 48 hours, the

For peer review only - http://bmjopen.bmj.com/site/about/guidelines.xhtml

parents will be asked if they are willing to remain at the hospital until 48 hours. If a child's health has stabilised by 48 hours and is ready to be discharged, direct observation will end after 48 hours. Otherwise data collection will continue until 72 hours. If a patient is unstable after 72 hours, he/she will remain at the hospital for further consultation by the hospital, but no further data will be collected until a final physical exam at discharge. Children who show abnormalities in the ultrasound scans that may be due to suspected thiamine deficiency and those with neurological symptoms at the time of discharge will be invited for a follow up physical exam after 2-6 weeks.

Procedures in the community

After obtaining written consent for the child's and mother's participation, a study nurse will perform a brief physical exam of the child, assessing the same vital signs as for hospitalised children (Supplementary Table 1). A venous blood sample will be collected from both child and mother for immediate processing in a mobile laboratory, anthropometric measurements will be conducted, breastmilk collected and questionnaires completed.

Children will be referred to the nearest health centre in the case of severe acute malnutrition (weight-for-length z-score <-3 SD[27]) and acute illnesses. In case of haemoglobin concentrations <100 g/L in both children and their mothers, the parent of the anemic child and/or the anemic mother will be informed by phone and referred to the nearest health centre for moderate or severe anaemia.

Blood sample collection

Venous blood will be collected from children and their mothers by trained nurses or laboratory technicians. Prior to the first thiamine administration, 5 mL venous blood will be collected from children into 6 mL BD vacutainer® tubes coated with K_2 EDTA (ref 367863, Becton, Dickinson & Company, Franklin Lakes, NJ, USA). From the children's mothers, 10 mL venous blood samples will be collected into 10 mL BD vacutainer® tubes coated with K_2 EDTA (ref 368589, Becton, Dickinson & Company, Franklin Lakes, NJ, USA). Blood samples will be processed immediately after they are collected, either at the hospital laboratory or in a mobile field laboratory station that will be established in each community. In particular, efforts will be made to analyse the blood for

For peer review only - http://bmjopen.bmj.com/site/about/guidelines.xhtml

lactate concentration within 30 minutes and to wash the red blood cells within 2 hours of the blood draw, as described in more detail below.

Fresh venous blood will be used for determination of the lactate concentration by StatStrip® Lactate (Nova Biomedical, Waltham, MA, USA) among hospitalised children, and analysis of the complete blood count (CBC) by haematology analyser (BC-3000 Plus Auto Hematology Analyzer, Shenzhen Mindray Bio-Medical Electronics Co. Ltd., Nanshan, Shenzhen, China). In the community, a small whole blood aliquot will be stored at 4-10°C and analysed for CBC upon arrival at the hospital laboratory, and within 8 hours of blood collection. For later assessment of ThDP, whole blood will be aliquoted into amber microcentrifuge tubes and stored at -80°C. From a convenience sample of mothers (n~200) with a complete 10 mL blood sample, 75 µl of blood will be pipetted into each of five circles of a dried blood spot card (Whatman 903 Protein Saver).

The remaining blood will be centrifuged at 3200 rpm for 10 minutes (DM0412S, DLAB Scientific Co., Ltd., Beijing, China). This speed corresponds to 1202 x g for the children's and to 1278 x g for the mothers' vacutainer tubes, respectively. Plasma will be aliquoted into microcentrifuge tubes of various volumes (0.2 to 1.5 mL) and types (amber and clear). After transferring the entire buffy coat carefully into a 1.5 mL microcentrifuge tube, packed erythrocytes will be washed three times in physiological saline (0.9% NaCl) and centrifuged at 4000 rpm for 10 minutes each time, which corresponds to 1878 x g and 2057 x g for children's and mother's tubes. All samples aliquoted in the community for later assessment of thiamine and other nutritional and health indicators will be frozen in an electronic cool box at -20°C and transferred to the -80°C freezer upon arrival at the hospital.

Blood biomarker analysis

The concentration of ThDP will be measured using the well-established thiochrome reaction coupled with high-performance liquid-chromatography fluorescence detection method[28,29]. The assay of ETK will be completed by UV spectrophotometer at the NIHR BRC Nutritional Biomarker Laboratory at the University of Cambridge in the United Kingdom. The activity of transketolase is measured in several steps: 1) the basal activity of transketolase is measured in erythrocytes (basal ETK); 2) the ETK activity is then determined in-vitro by adding exogenous thiamine diphosphate in excess (ETK activity), and; 3) the ETKac is then calculated by dividing the ETK activity by the basal ETK. C-reactive protein (CRP) and alpha-1-acid-glycoprotein (AGP) will be analysed by the enzyme-linked immunosorbent assay (ELISA).

For peer review only - http://bmjopen.bmj.com/site/about/guidelines.xhtml

Breastmilk sample collection and biomarker analysis

Breastmilk collection will be attempted from all lactating mothers. Because mothers of children with suspected beriberi are routinely prescribed oral thiamine supplementation at LFHC (100 mg twice daily for 30 days as per WHO recommendations[22]), breastmilk and maternal blood will be collected prior to any supplement intake.

Prior to breastmilk collection, the breast will be cleaned with a sanitary wipe (Hygea® Obstetrical Towelette, PDI Healthcare, Orangeburg, NY, USA). Breastmilk of the fuller breast will be collected into a breastmilk bag with an electronic hospital-grade breast pump (Symphony pump, Medela AG, Baar, Switzerland) until the breast is emptied. Because circadian differences in concentrations of thiamine and other vitamins are small[28], the breastmilk sample will be collected at any time of day. Time of day, time since last meal, breast side, time since last emptying of that side, and milk volume will be recorded. Samples will be mixed well before aliquoting into 1.5 mL amber and clear microcentrifuge tubes and frozen at -80°C until analysis at the USDA Western Human Nutrition Research Center at UC Davis (USA). Samples will be analysed for thiamine concentration by ultra-performance liquid chromatography tandem mass-spectrometry using internal pooled breastmilk samples for quality control[30].

Specimen banking

Any remaining biochemical samples (whole blood, red blood cells, plasma, dried blood spots and breastmilk) will be stored at UC Davis for possible future analyses of indicators of interest.

Ultrasound performance and interpretation

An echocardiogram and cranial ultrasound will be performed with a portable ultrasound machine (M9, Shenzhen Mindray Bio-Medical Electronics Co. Ltd., Nanshan, Shenzhen, China) in all hospitalised children. For the echocardiogram, study physicians will capture two second video clips of the heart in apical four chamber, parasternal long axis and parasternal short axis views. Required still images for standard measurements will include: left ventricular dimensions in diastole and systole by M-mode, E-point septal separation in the parasternal long axis, and tricuspid annular plane systolic excursion[31]. For the cranial ultrasound, study physicians will capture six standard coronal and five standard sagittal still images of the brain, with additional

For peer review only - http://bmjopen.bmj.com/site/about/guidelines.xhtml

images of the putamina, caudate nuclei, and thalamus. Cranial ultrasound will primarily be performed among infants. However, we will attempt cranial ultrasound among >12 months old children in case the anterior fontanelle has not yet closed entirely, which may allow ultrasonography of the basal ganglia. The echocardiogram will be completed at baseline (i.e. prior to or soon after the first thiamine administration) and repeated at 24 and 48 hours. Because normalisation of encephalitic beriberi occurs slowly[12], the cranial ultrasound will be performed once during the hospital stay, typically within the first 12 hours.

All ultrasonography completed by study physicians or nurses will be uploaded to Box (Box Inc, Redwood City, CA, USA). All baseline echocardiograms and cranial ultrasounds will be reviewed by one of two expert sonographers at UC Davis. If the baseline echocardiogram is flagged as abnormal by one of these reviewers, the 24 and 48 hour echocardiograms will also be reviewed by these same reviewers. In addition, all abnormal echocardiograms and cranial ultrasounds will be reviewed by a pediatric cardiologist and a pediatric radiologist at UC Davis respectively, who will provide a description of the observed abnormality. Any abnormality identified by the pediatric cardiologist and/or pediatric radiologist will be communicated to the medical leadership of the LFHC to initiate appropriate action. In case of abnormalities suggestive of thiamine deficiency, the study children will be invited for a follow up exam after 2-4 weeks in case of an abnormal echocardiogram and after 6 weeks in case of an abnormal cranial ultrasound, respectively.

For quality assurance, 10% of echocardiograms and cranial scans judged as normal will be randomly selected and re-read by one of the pediatric cardiologists and the pediatric radiologist at UC Davis, respectively. If an echocardiogram is judged as normal during the first review, the 24 and 48 hour echocardiograms will typically not be reviewed. However, 10% of randomly selected 24 and 48 hour echocardiograms will be re-read by the UC Davis expert sonographers to verify the description of the baseline echocardiography.

Anthropometric assessment

Anthropometry of both children and their mothers will be assessed in the hospital and the community following protocols proposed by the Food and Nutrition Technical Assistance III Project[32]. Unclothed or lightly dressed children will be weighed to the nearest 20 g (SECA 383, Hamburg, Germany). Children's recumbent length (SECA 417, Hamburg, Germany), left mid-upper am circumference (MUAC; Shorr Child MUAC Tape, Weigh and Measure, Olney, MD, USA) and head circumference (ShorrTape© 65cm Measuring Tape, Weigh and Measure, Olney, MD, USA) will be measured to the nearest 0.1 cm. All measurements will be

For peer review only - http://bmjopen.bmj.com/site/about/guidelines.xhtml

collected in duplicate and the average of the two measurements will be calculated. If measurements differ by >0.1 kg (weight), or >0.5 cm (length, MUAC, head circumference), the measurement will be repeated a third time and the average of the two closest measurements will be calculated. Maternal weight (SECA 874, Hamburg, Germany), height (SECA 213, Hamburg, Germany) and left MUAC (ShorrTape© 65cm Measuring Tape) will be assessed using a similar approach. Regular standardisation sessions will be implemented to compare the performance of anthropometry teams among themselves and with their supervisors[33,34].

Risk factor and predictor assessment

A broad range of information will be collected using structured questionnaires (**Supplementary Table 1**). Data collection forms were developed in English, and programmed in Survey CTO (Dobility Inc., Cambridge, MA, USA) and translated into Lao for electronic data collection.

Specific questionnaires were developed to assess the child's birth history, and the child and mother's health history. Because traditional restrictive postpartum diets are very common in the study population, an extensive questionnaire was developed to record dietary patterns both during pregnancy and the postpartum period. Other questionnaires, such as the assessment of socio-economic status, and child and mother dietary practice questionnaires, were adapted from the Lao Zinc Study[35]. Because women and children may change their dietary intake due to their presence at the hospital in Luang Prabang, we will ask the mother to report food intake for the days prior to their arrival at the hospital. Household food security will be assessed using the Household Food Insecurity Access Scale[36], and social desirability of respondents will be assessed as proposed by Crowne and Marlowe [37,38]. Two brief questionnaires will be used to record the reason for exiting the study by children and mothers, respectively (i.e. completion of data collection, withdrawal of consent, death, or other reasons).

Patient and public involvement

Patients and the public were not involved in the design of this study.

For peer review only - http://bmjopen.bmj.com/site/about/guidelines.xhtml

Case report review

To develop a clinician guided diagnostic impression of TRD, three expert paediatricians specialised in tropical medicine will review the case reports of each hospitalised child to judge whether a child had TRD using a 3 point scale (probable TRD (including classic beriberi); possible TRD; not likely TRD). Specifically, each case report will be reviewed by three expert paediatricians. When there is disagreement (defined as separate raters classifying the same case as probable TRD and not likely TRD) or when there is no agreement (i.e. each rater classifies a case into a different category) efforts will be made to reach consensus between the three experts. A case report template was developed for this purpose, which includes the basic demographics of the child (sex, age), the primary concerns of the parents for taking their child to the hospital, relevant data collected during the baseline and follow up physical exams (4, 8, 12, 24, 36, 48, 72 hrs and hospital discharge), the recovery status over the course of the hospital stay, administered treatments, findings from the echocardiogram and the cranial ultrasound, primary and secondary diagnosis by the LFHC treating physicians at the child's discharge from hospital, dietary practices and maternal characteristics. The latter include exclusive breastfeeding, maternal dietary practices with regard to a restricted postpartum diet and whether the mother experiences tingling in her fingers, an indication that the mother herself may experience clinical manifestations of severe thiamine deficiency. In addition to the case report, the expert panel will be able to review additional study data as needed.

For peer review only

Data management and statistics

Electronic data collection, data management and data quality control

Participants will be given a unique 4-digit numeric study ID code, which will link all data collected from this individual. To link the data from a child mother pair, the same 4-digit ID code will be given to each with the exception that the mothers' data will be identified with the letter "M" before the 4-digit ID code.

All data will be collected electronically using Survey CTO, and pre-designed, pre-coded paper forms will be available for back up, if needed. Data collection forms were developed in a customised Survey CTO application by a UC Davis data manager, and, where appropriate, forms include pre-determined checks and range validations. Forms will be deployed onto Samsung tablets (Samsung Galaxy Tab. 3 V, Seoul, South Korea)

For peer review only - http://bmjopen.bmj.com/site/about/guidelines.xhtml

equipped for mobile internet access. All data forms collected will first be reviewed by one of two study data managers and then synchronised onto a central server.

Data quality checks will be run weekly and track information such as form completion, internal consistency, specimen sample processing, realistic data ranges, eligibility criteria, child's timeline within the study, and translation of local languages to English. Feedback will be regularly communicated to study staff to ensure consistent high quality data collection.

Sample size

For our primary research question, we aim to develop and compare clinical prediction rules that are useful in determining which children with TDD-like symptoms would respond favourably to thiamine treatment. Given the context of the study, there is uncertainty around the expected prevalence of TRD in our sample and so we anticipate that between 10 to 90% of the hospitalised cohort will be classified as responders. Assuming that a clinically useful discriminative capacity will correspond to an area under the receiver operating characteristic curve (AUROC) of 0.70 for the responders *vs.* nonresponder classification, we will test the null hypothesis that the AUROC is 0.5 using a two-sided test with alpha = 0.05. To account for the selection of independent variables for our multivariable model, we derived a variance inflation factor of 1.88 by computing the ratio of the Chi-square noncentrality parameters corresponding to 95% power, 2-sided alpha, with 10 and 1 degrees of freedom, respectively (24.386 (for 10 df) divided by 12.995 (for 1 df)). Additional variance inflation factors of 1.25 and 1.25 were needed to account for 20% loss to follow-up and 20% probability of blood collection failure, respectively. Hence, we anticipate that a total enrolment of 662 hospitalised children will, after correcting for these three variance inflation factors, result in an effective sample size of 225 for power and sample size purposes. According to the SAS ROCPOWER macro (SAS 9.4; SAS Institute), an effective sample size of 225 would provide at least 90% power to detect a model with a discriminative capacity corresponding to an AUROC of 0.70, under two-sided testing with alpha=5% given a TRD prevalence of between 11 and 89%.

For our secondary research questions concerned with describing TRD risk factors across a community comparison cohort and our two hospital cohorts, we plan to enrol 212 region, age and sex frequency-matched children (increased to 265 after adding the inflation factor of 1.25 for potential blood collection failure). Given a hospital cohort split between TRD groups of sizes 60 to 106 and 424 to 470, this would provide at least 80% power to detect an independent variable for which a 1-standard deviation increase is associated with an adjusted odds ratio of 1.6, under two-sided testing (of the null hypothesis that the adjusted odds ratio is 1)

For peer review only - http://bmjopen.bmj.com/site/about/guidelines.xhtml

with alpha=5%, even when other covariates in the models account for as much as 20% of the variation in that independent variable. For a binary risk factor present in 20% of children, the detectable adjusted odds-ratio under the stated assumptions would be 2.45. Hence, we anticipate having adequate statistical power to detect risk factors associated with moderate to strong incremental discriminative capacity.

Statistical analysis

Prior to performing statistical analyses, detailed statistical analysis plans will be developed and published online[39].

Our primary model development objective will seek to achieve case definition models that provide high discrimination capacity and acceptable calibration in external samples, using principled approaches to guard against overfitting biases. It is likely there will be ambiguity in TRD determination due to a range of potential thresholds for what is considered "recovery". Consequently, sensitivity analyses may be repeated with different variations of TRD definitions. These definitions will be defined prior to analysis by expert paediatricians. We will follow a three-staged approach to identifying candidate risk factors and selecting from among them. In the first stage, investigators will specify risk predictors that could be reliably and validly measured early in the hospitalisation in limited-resource settings and which are plausibly associated with TRD response status. In the second stage, we will develop and validate logistic regression models, using TRD response status as the dependent variable, and using elastic net regression and related approaches to principled variable selection. In the third stage, we will repeat the analysis incorporating more sophisticated diagnostic assessments, such as biomarkers and ultrasound, which are available in higher resource settings. Finally, after each clinical prediction model is developed, we will use resampling methods with 10-fold cross-validation to estimate model performance in terms of discriminative capacity, as measured by empirical AUROC, internally and externally.

Our secondary objective of establishing biomarker cut-offs for TRD will follow a similar receiver operating characteristic curve based approach, except with specific biomarker concentrations as the principle predictor. A weighted evaluation of each cut-offs sensitivity and specificity will be used in a 10-fold cross-validation framework to select the optimal cut-off[40].

The comparison of predictors, biomarkers, and risk factors across the community cohort, non-TRD hospital cohort, and TRD hospital cohort will be contextual and dependent on the type of variable considered. Analyses will be primarily descriptive, focused on substantive differences between cohorts, accompanied by visualisations and distribution appropriate statistical testing.

For peer review only - http://bmjopen.bmj.com/site/about/guidelines.xhtml

ETHICS AND DISSEMINATION

The study protocol was reviewed and approved by a steering committee at the Ministry of Health (MOH) and the Ministry of Foreign Affairs in Lao PDR. The study and consenting procedures were approved by the National Ethics Committee for Health Research, Ministry of Health, Lao PDR (ref. 11/2019) and the Institutional Review Board (IRB) of the UC Davis (ref. 1329444). The trial is registered at https://clinicaltrials.gov (NCT03626337). The executive leadership of LFHC also approved the conduct of the study as described here.

The presented protocol version (Version 2019-11-12) includes changes to the inclusion criteria, described in detail above. Any future modifications to the protocol including changes of study objectives, study design, inclusion criteria, sample sizes, study procedures, or significant administrative aspects will be agreed upon by the principal investigators and co-investigators, and approved by the National Ethics Committee for Health Research, Ministry of Health, Lao PDR and the UC Davis IRB prior to implementation. The present study will be implemented among children who will be admitted to the hospital for TDD like symptoms. Study participants will be treated by the hospital physicians following the usual hospital procedures, and the study team will not interfere with the administration of required treatments and interventions. The one study-specific intervention administered to all hospitalised study participants is thiamine. Thiamine is a water-soluble vitamin prescribed in case of beriberi or thiamine deficiency[10,22]. Documented side effects include: nausea, flushing sensation, restlessness, sweating and weakness, and there may be hypersensitivity reaction to intravenous injection or tenderness at the intramuscular injection site[41]. All children will be examined for these symptoms over the course of the study (**Table 1**), and any observed adverse events will be documented. Serious adverse events that are unexpected and/or probably related to the research procedures will be reported immediately to the UC Davis IRB and the National Ethics Committee for Health Research in Lao PDR.

A Data and Safety Monitoring Board (DSMB) will be established for the purpose of this project. Three independent paediatricians will monitor patient safety while the study is ongoing. Interim analyses will be completed only for safety considerations. Reports of the occurrence of potential adverse events will be submitted to and reviewed by the DSMB members. A new report will be shared with the DSMB committee each time a quarter of study participants are enroled. The principal investigators have the right to terminate

For peer review only - http://bmjopen.bmj.com/site/about/guidelines.xhtml

the study if the incidence of side effects and/or severity of adverse events outweigh the benefits of the study. Because thiamine administration is an established intervention with mild temporary side effects, it is expected that the study will continue until planned completion. Detailed stopping rules have not been designed at this time.

Study results will be reported following recommendations by the International Committee of Medical Journal Editors[42], and in accordance with the TRIPOD Statement and other relevant guidelines from the EQUATOR Network[43,44]. The knowledge and information gained from the present study will be promptly and broadly disseminated through peer-reviewed scientific open access articles, and through communications and presentations at conferences. Moreover, the present study is part of a larger multi-country effort to address critical questions related to thiamine deficiency and public health in Asia and Africa[45,46]. An important aspect of the overall project is to exchange research results among the collaborators. In-country result dissemination will be led by the Lao Tropical and Public Health Institute through meetings and workshops with representatives of the Ministry of Health, the Provincial Health Departments and other key stakeholders. Research briefs will also be produced for in-country distribution. In addition, the findings will be presented at the Annual Lao Pediatric Continuing Medical Education Conference organised by the Lao Pediatric Association and at the National Health Research Forum in Vientiane, Lao PDR.

TRIAL STATUS

The first participants of the hospital and community cohort were enroled in June 2019. Changes to the enrolment criteria were implemented in November 2019 (after 18.1% of the hospital and 13.6% of the community target sample size had been enroled). We anticipate data collection will be completed by December 2020.

ACKNOWLEDGEMENTS

For peer review only

We are grateful for the valuable input during the proposal development by Professor Paul Newton (Lao-Oxford-Mahosot Hospital-Wellcome Trust Research Unit, Vientiane, Lao PDR), Professor Kenneth Brown (Institute for Global Nutrition, UC Davis, Davis, CA, USA), and Dr. Sommanikhone Phangmanixay (Children Hospital, Vientiane, Lao PDR), and for Dr. Somphou Sayasone's (Lao Tropical and Public Health Institute, Vientiane, Lao PDR) administrative support throughout the study preparation and implementation. We appreciate that Dr. David Brown (Department of Cardiology, Boston Children's Hospital, Boston, MA, USA) and Kirk Stonick (Kaiser Permanente, Portland, OR, USA) traveled to Luang Prabang to conduct a one-week training in echocardiograms and cranial ultrasonography, respectively, and Dr. Kethmany Ratsavong's (Lao Tropical and Public Health Institute, Vientiane, Lao PDR) help with English to Lao translations during study staff trainings. We thank all physicians, nurses, laboratory technicians and administrative staff of the Lao Friends Hospital for Children (Luang Prabang, Lao PDR) for their collaboration, with special thanks to the Medical Director, Dr. Lisa Rynn. We thank Dr. Aviva Fattal-Valevski (Tel Aviv University, Tel Aviv, Israel) for providing input on the physical data collection form and the members of the DSMB (Dr. Charles Larson, School of Population and Global Health, McGill University, Montreal, Canada; Dr. Bandith Soumpholphakdy, Mahosot Hospital, Vientiane, Lao PDR; Dr. Daniel Roth, Pediatrician, The Hospital for Sick Children, Toronto, Canada) for their willingness to review data. Lastly, we are grateful to Medela AG (Baar, Switzerland) to lend hospital-grade breastmilk pumps for the duration of the study and to donate breastmilk collection supplies.

For peer review only

AUTHOR CONTRIBUTORS

SYH led the proposal writing. SYH and TJS developed all data collection tools and standard operational procedure manuals. PRF, IT and LH developed the thiamine treatment and physical examination protocols. MAS, JY, RSW and CNM developed different aspects of the sonography protocols; MAS, CNM and KN prepared the sonography training plan. CDA and DJT developed the statistical analyses plan. XT programmed all data collection tools and developed the data management protocol together with CDA. SK, DS, TJS and SYH planned local study implementation. The manuscript was drafted by SYH, TJS and CDA. All coauthors reviewed the manuscript critically and approved the final content.

FUNDING

This work is supported by the Bill & Melinda Gates Foundation (grant number OPP1182864). Dr. Kenneth Brown provided technical support in the design of this study, when working for the Bill & Melinda Gates

For peer review only - http://bmjopen.bmj.com/site/about/guidelines.xhtml

Foundation. The funding source will not have any role during its execution, analyses, interpretation of the data, or decision to submit results.

COMPETING INTERESTS

All authors have completed the ICMJE uniform disclosure form at www.icmje.org/coi_disclosure.pdf and declare: SYH has completed a consultancy for the Bill & Melinda Gates Foundation and her spouse previously worked for the Bill & Melinda Gates Foundation and; no other relationships or activities that could appear to have influenced the submitted work.

DATA SHARING STATEMENT

The complete de-identified dataset will be made publicly available 3 years after completion of data collection on Open Science Framework at https://osf.io/jfke3.

REFERENCES

1. Johnson CR, Fischer PR, Thacher TD, et al. Thiamin deficiency in low- and middle-income countries: Disorders, prevalences, previous interventions and current recommendations. *Nutrition and Health* 2019;25:127-51.
2. Whitfield KC, Bourassa MW, Adamolekun B, et al. Thiamine deficiency disorders: diagnosis, prevalence, and a roadmap for global control programs. *Annals of the New York Academy of Sciences* 2018;1430:3-43.
3. Barennes H, Sengkhamyong K, Rene JP, et al. Beriberi (thiamine deficiency) and high infant mortality in northern Laos. *PLoS Negl Trop Dis* 2015;9:e0003581.
4. Khounnorath S, Chamberlain K, Taylor AM, et al. Clinically unapparent infantile thiamin deficiency in Vientiane, Laos. *PLoS Negl Trop Dis* 2011;5:e969.
5. Soukaloun D, Lee SJ, Chamberlain K, et al. Erythrocyte transketolase activity, markers of cardiac dysfunction and the diagnosis of infantile beriberi. *PLoS Negl Trop Dis* 2011;5:e971.
6. Barennes H, Simmala C, Odermatt P, et al. Postpartum traditions and nutrition practices among urban Lao women and their infants in Vientiane, Lao PDR. *European Journal of Clinical Nutrition* 2009;63:323-31.
7. Holmes W, Hoy D, Lockley A, et al. Influences on maternal and child nutrition in the highlands of the northern Lao PDR. *Asia Pacific Journal of Clinical Nutrition* 2007;16:537-45.
8. Soukaloun D, Kounnavong S, Phengdy B, et al. Dietary and socio-economic factors associated with beriberi in breastfed Lao infants. *Annals of Tropical Paediatrics* 2003;23:181-6.

For peer review only - http://bmjopen.bmj.com/site/about/guidelines.xhtml

For peer review only

9. Allen LH. B vitamins in breast milk: relative importance of maternal status and intake, and effects on infant status and function. *Adv Nutr* 2012;3:362-9.
10. World Health Organization. Thiamine deficiency and its prevention and control in major emergencies. Geneva: World Health Organization, 1999.
11. Hiffler L, Rakotoambinina B, Lafferty N, et al. Thiamine Deficiency in Tropical Pediatrics: New Insights into a Neglected but Vital Metabolic Challenge. *Front Nutr* 2016;3:16.
12. Wani NA, Qureshi UA, Ahmad K, et al. Cranial Ultrasonography in Infantile Encephalitic Beriberi: A Useful First-Line Imaging Tool for Screening and Diagnosis in Suspected Cases. *AJNR American Journal of Neuroradiology* 2016;37:1535-40.
13. Carreras I, Martinesz Garcia D, Indi EP, et al. Thiamine deficiency in neurological acute disorders in resource-limited settings: case reports. 3rd World Congress on Controversies in Pediatrics; 2016; Barcelona, Spain.
14. Duce M, Escriba J, Masuet C, et al. Suspected thiamine deficiency in Angola. *Field Exch* 2003;20:26-28.
15. Ogunlesi TA. Thiamine deficiency: a cause of childhood ataxia not to be ignored. *Annals of Tropical Paediatrics* 2004;24:357-60.
16. Keating EM, Nget P, Kea S, et al. Thiamine deficiency in tachypnoeic Cambodian infants. *Paediatr Int Child Health* 2015;35:312-8.
17. Coats D, Shelton-Dodge K, Ou K, et al. Thiamine deficiency in Cambodian infants with and without beriberi. *Journal of Pediatrics* 2012;161:843-7.
18. Lima LF, Leite HP, Taddei JA. Low blood thiamine concentrations in children upon admission to the intensive care unit: risk factors and prognostic significance. *American Journal of Clinical Nutrition* 2011;93:57-61.
19. Gibson RS. Principles of nutritional assessment. 2nd ed. New York: Oxford University Press 2005.
20. Talwar D, Davidson H, Cooney J, et al. Vitamin B(1) status assessed by direct measurement of thiamin pyrophosphate in erythrocytes or whole blood by HPLC: comparison with erythrocyte transketolase activation assay. *Clinical Chemistry* 2000;46:704-10.
21. Herve C, Beyne P, Letteron P, et al. Comparison of erythrocyte transketolase activity with thiamine and thiamine phosphate ester levels in chronic alcoholic patients. *Clinica Chimica Acta* 1995;234:91-100.
22. World Health Organization Western Pacific Region. Pocket book of hospital care for children. Guidelines for the management of common childhood illnesses. 4 ed. Manila, The Philippines: World Health Organization Western Pacific Region 2017.
23. Frank LL. Thiamin in Clinical Practice. *JPEN Journal of Parenteral and Enteral Nutrition* 2015;39:503-20.
24. Crook MA, Sriram K. Thiamine deficiency: the importance of recognition and prompt management. *Nutrition* 2014;30:953-4.
25. McCandless DW. Beriberi. Thiamine Deficiency and Associated Clinical Disorders Contemporary Clinical Neuroscience: Humana Press 2010.
26. Park SW, Yi YY, Han JW, et al. Wernicke's encephalopathy in a child with high dose thiamine therapy. *Korean J Pediatr* 2014;57:496-9.
27. WHO Multicentre Growth Reference Study Group. WHO Child Growth Standards: Length/height-for-age, weight-for-age, weight-for-length, weight-for-height and body mass index-for-age: Methods and development. Geneva: World Health Organization, 2006.
28. Hampel D, Shahab-Ferdows S, Islam MM, et al. Vitamin Concentrations in Human Milk Vary with Time within Feed, Circadian Rhythm, and Single-Dose Supplementation. *Journal of Nutrition* 2017;147:603-11.
29. Lu J, Frank EL. Rapid HPLC measurement of thiamine and its phosphate esters in whole blood. *Clinical Chemistry* 2008;54:901-6.
30. Hampel D, York ER, Allen LH. Ultra-performance liquid chromatography tandem mass-spectrometry (UPLC–MS/MS) for the rapid, simultaneous analysis of thiamin, riboflavin, flavin adenine dinucleotide, nicotinamide and pyridoxal in human milk. *Journal of Chromatography B* 2012;903:7-13.

For peer review only

For peer review only - http://bmjopen.bmj.com/site/about/guidelines.xhtml

31. Lopez L, Colan SD, Frommelt PC, et al. Recommendations for quantification methods during the performance of a pediatric echocardiogram: a report from the Pediatric Measurements Writing Group of the American Society of Echocardiography Pediatric and Congenital Heart Disease Council. *Journal of the American Society of Echocardiography* 2010;23:465-95; quiz 576-7.
32. Cashin K, Oot L. Guide to anthropometry: A practical tool for program planners, managers, and implementers. Washington DC: Food and Nutrition Technical Assistance III Project (FANTA)/ FHI 360, 2018.
33. de Onis M, Onyango AW, Van den Broeck J, et al. Measurement and standardization protocols for anthropometry used in the construction of a new international growth reference. *Food and Nutrition Bulletin* 2004;25:S27-36.
34. World Health Organization. Recommendations for data collection, analysis and reporting on anthropometric indicators in children under 5 years old. Geneva, Switzerland: World Health Organization and United Nations Children's Fund, 2019.
35. Wessells KR, Brown KH, Kounnavong S, et al. Comparison of two forms of daily preventive zinc supplementation versus therapeutic zinc supplementation for diarrhea on young children's physical growth and risk of infection: study design and rationale for a randomized controlled trial. *BMC Nutrition* 2018;4:39.
36. Coates J, Swindale A, Bilinsky P. Household Food Insecurity Access Scale (HFIAS) for measurement of food access: Indicator guide. In: FANTA, ed. Washington, DC: USAID, 2007.
37. Crowne DP, Marlowe D. A new scale of social desirability independent of psychopathology. *Journal of Consulting Psychology* 1960;24:349-54.
38. Menon P, Nguyen PH, Saha KK, et al. Combining Intensive Counseling by Frontline Workers with a Nationwide Mass Media Campaign Has Large Differential Impacts on Complementary Feeding Practices but Not on Child Growth: Results of a Cluster-Randomized Program Evaluation in Bangladesh. *Journal of Nutrition* 2016;146:2075-84.
39. Hess SY, Smith TJ, Arnold CD. Lao Thiamine Study: Open Science Framework; 2019 [Available from: https://osf.io/jfke3 accessed July 31 2019.
40. Perkins NJ, Schisterman EF. The inconsistency of "optimal" cutpoints obtained using two criteria based on the receiver operating characteristic curve. *American Journal of Epidemiology* 2006;163:670-5.
41. American Pharmacists Association. Lexicomp Drug Information Handbook: A Clinically Relevant Resource for All Healthcare Professionals 27 ed: Wolters Kluver 2018-2019.
42. International Committee of Medical Journal Editors. Recommendations for the conduct, reporting, editing, and publication of scholarly work in medical journals: ICMJE; 2019 [Available from: http://www.icmje.org/recommendations/ accessed November 19 2019.
43. Collins GS, Reitsma JB, Altman DG, et al. Transparent reporting of a multivariable prediction model for individual prognosis or diagnosis (TRIPOD): the TRIPOD statement. *BMJ* 2015;350:g7594.
44. Equator Network. Enhancing the quality and transparency of health research 2019 [cited 2019. Available from: http://www.equator-network.org/ accessed September 9 2019.
45. Whitfield KC, Kroeun H, Green T, et al. Thiamine dose response in human milk with supplementation among lactating women in Cambodia: study protocol for a double-blind, four-parallel arm randomised controlled trial. *BMJ Open* 2019;9:e029255.
46. Bergeron G, Bourassa M. Overcoming the current challenges of thiamine deficiency disorders *Current Developments in Nutrition* 2019;3:nzz034.P10-91-19.

For peer review only - http://bmjopen.bmj.com/site/about/guidelines.xhtml

Table 1: Timing of study procedures of hospitalised study children and their mothers

Activity	Prior to enrolment	Baseline	Medical treatment	Hours and weeks after thiamine administration										
				0-1 hr	4 hr	8 hr	12 hr	24 hr	36 hr	48 hr	72 hr	>72 hr[2]	2-4 wks[3]	6 wks[4]
Child's study participation and data collection														
Care seeking at hospital	X													
Parental consent for child's participation[1]	X													
Enrolment		X												
Venous blood sample		X												
Thiamine administration			X					X		X				
Other clinically indicated treatments			XXXXXXXXXXXXXXXXXXXXXXXXXXXXXXXXXXXXXXXXXXXXXXXXXXXXXXXXXXXXXXXXXXXX											
General physical exam		X			X	X	X	X	X	X	X	X	X	X
Liver palpation		X			X	X	X	X	X	X	X	X	X	X
Heart rate		X			X	X	X	X	X	X	X	X	X	X
Exam for oedema		X			X	X	X	X	X	X	X	X	X	X
Respiratory rate, oxygen saturation		X			X	X	X	X	X	X	X	X	X	X
Eye movement/contact assessment		X			X	X	X	X	X	X	X	X	X	X
Signs of ptosis and ophthalmoplegia		X			X	X	X	X	X	X	X	X	X	X
Hypotonia (head lag, floppy arms or legs in face-down position)		X			X	X	X	X	X	X	X	X	X	X
Consciousness evaluation		X			X	X	X	X	X	X	X	X	X	X
Vomiting frequency		X			X	X	X	X	X	X	X	X	X	X
Feeding behaviour		X			X	X	X	X	X	X	X	X	X	X
Anthropometry								X						
Echocardiogram		X						X		X			X	
Cranial ultrasound				X										X
Potential adverse events from thiamine injection			X	X	X	X	X	X	X	X	X	X		

For peer review only

Questionnaires														
Basic child characteristics		X												
Child's birth and health history[5]					X									
SES characteristics and food security of child's family[5]					X									
Dietary practices of child[5]					X									
Mother's study participation and data collection														
Maternal consent	X[6]													
Maternal health[5]					X									
Maternal dietary practices[5]					X									
Maternal blood[7]					X									
Breast milk[7]					X									
Maternal anthropometry[7]										X				

[1] In case of life threatening condition, the child will be examined, blood will be drawn and all required medical interventions will be provided (including thiamine administration) prior to obtaining parental consent. Parental consent will be obtained as soon as the child's condition has stabilised. Any data that has been collected will only be used, if parental written consent is obtained.

[2] Final physical exam prior to hospital discharge, timing will depend on hospital duration and time of discharge.

[3]Children with abnormal echocardiograms due to suspected thiamine deficiency will be invited for a follow up physical exam and echocardiogram after 2-4 weeks

[4] Children with abnormal cranial ultrasounds or neurological symptoms due to suspected thiamine deficiency will be invited for a follow up physical exam and cranial ultrasound after 6 weeks.

[5] Questionnaires will be administered during the hospital stay of the child. Timing will depend on hospital procedures and physical exams.

[6] Maternal consent will be obtained prior to enrolment into the study. The timing of obtaining maternal consent will depend on the health status of her child and hospital procedures.

[7] Maternal blood and breast milk will be collected as soon as possible after the mother's written consent is obtained. Maternal anthropometry will be completed at a convenient time before the child's discharge.

For peer review only

For peer review only - http://bmjopen.bmj.com/site/about/guidelines.xhtml

Figure 1: Study design overview of hospitalised and community cohort in the Lao Thiamine Study[1]

[1] At the onset of the study, the eligibility age was 21 days to <12 months. The target age was expanded to <18 months after 18.1% of hospital infants had been enroled, due to much lower enrolment rates than anticipated, and after consultation with expert paediatricians and the hospital leadership.

Figure 2: Flow chart of data collection timing among hospitalised infants and young children in the Lao Thiamine Study

For peer review only

For peer review only - http://bmjopen.bmj.com/site/about/guidelines.xhtml

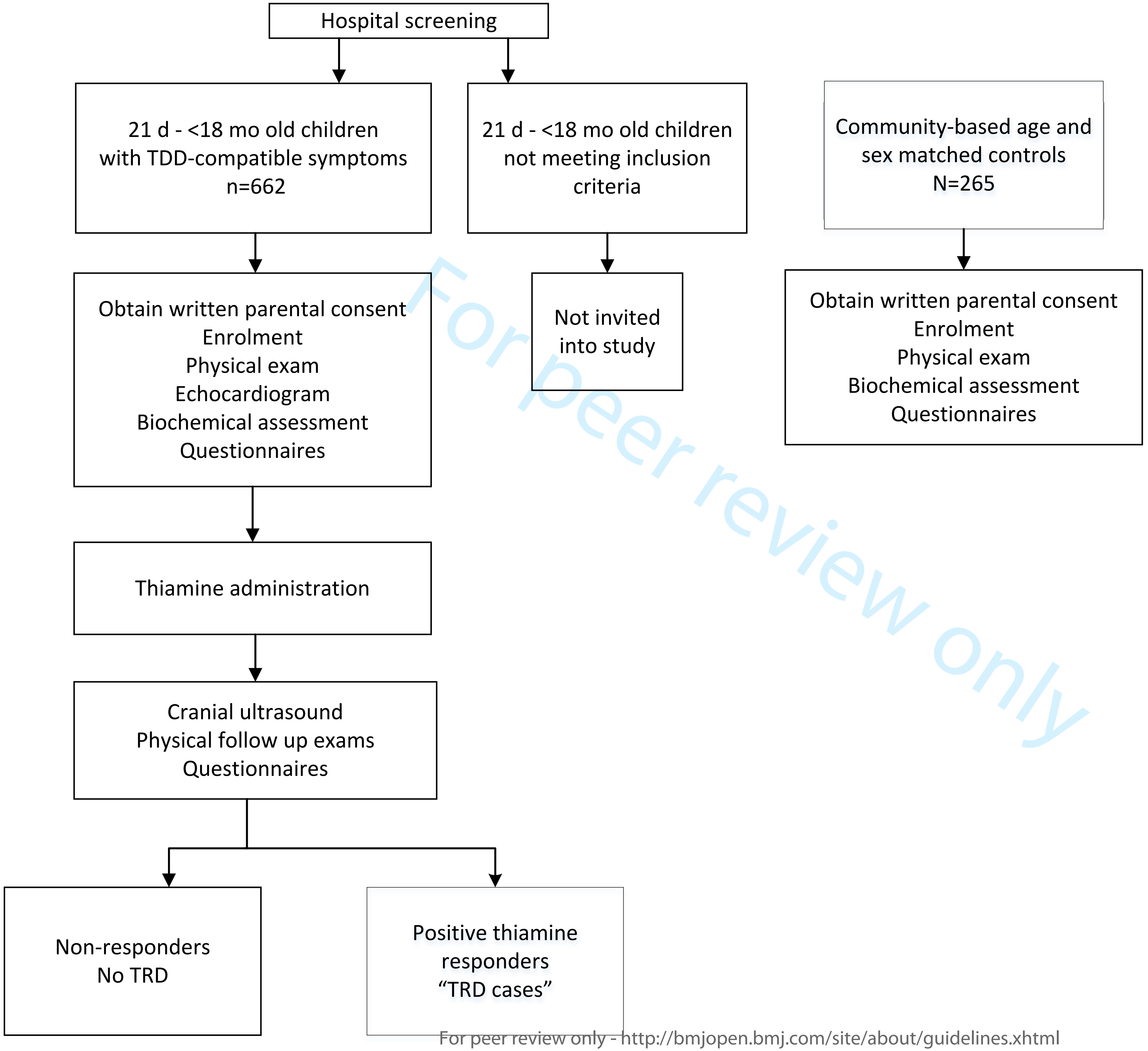

For peer review only - http://bmjopen.bmj.com/site/about/guidelines.xhtml

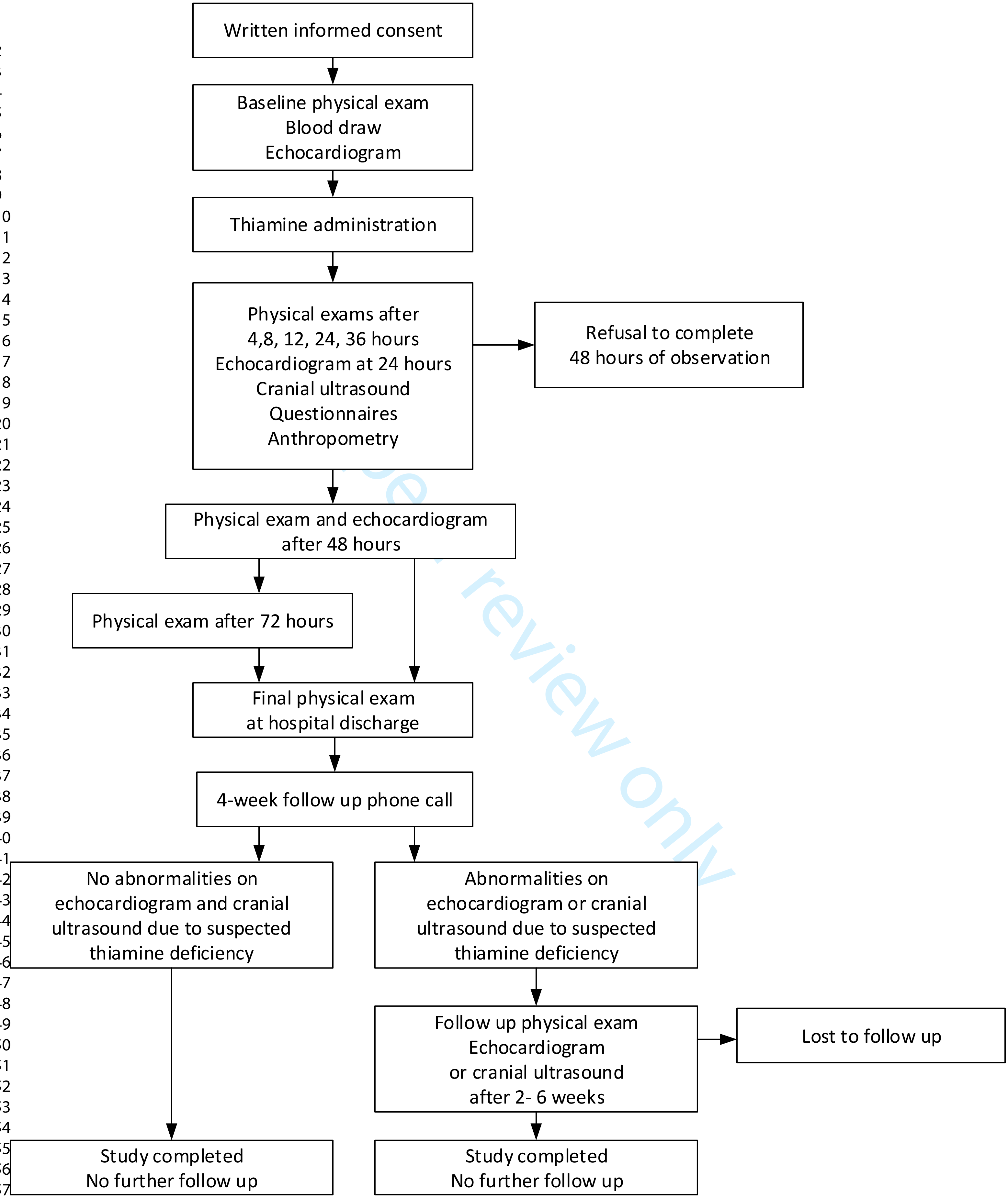
Written informed consent
Baseline physical exam
Blood draw
Echocardiogram
Thiamine administration
Physical exams after
4,8, 12, 24, 36 hours
Echocardiogram at 24 hours
Cranial ultrasound
Questionnaires
Anthropometry
Refusal to complete
48 hours of observation
Physical exam and echocardiogram
after 48 hours
Physical exam after 72 hours
Final physical exam
at hospital discharge
4-week follow up phone call
No abnormalities on
echocardiogram and cranial
ultrasound due to suspected
thiamine deficiency
Abnormalities on
echocardiogram or cranial
ultrasound due to suspected
thiamine deficiency
Follow up physical exam
Echocardiogram
or cranial ultrasound
after 2- 6 weeks
Lost to follow up
Study completed
No further follow up
Study completed
No further follow up

Supplementary Table 1: Inclusion criteria for study participants and outcomes of interest in the Lao Thiamine Study

**Inclusion criteria**	**Outcomes**		
	**Physical exams**	**Biochemical exams**	**Data collection via questionnaires**
Hospitalised children			
Target age range:<br>- 21 days to <18 months[1]<br><br>Plus at least one of the following criteria:<br>- Liver enlargement (>2cm below right costal margin, supine exam)<br>- OedemaTachypnea (> 60/min for 3-8 weeks; > 50/min for 2-11 moths; >40/min for 12 - 18 months)<br>- Tachycardia (heart rate >160/min for <12 months; >120/min for 12-18 months)<br>- Oxygen saturation < 92%<br>- Difficulty breathing (chest in-drawing or nasal flaring)<br>- Refusal to breastfeed or refusal of infant formula or food for greater than 24 hours<br>- Repetitive or recurrent vomiting with no obvious other cause (i.e. vomiting >3 times in past 24 hours)<br>- Persistent crying not relieved by soothing or feeding with no obvious other cause<br>- Hoarse voice/cry or loss of voice<br>- Nystagmus or other unusual eye movements<br>- Muscle twitching<br>- Loss of consciousness<br>- Convulsion<br>- Opisthotonus /abnormal posturing[2]<br>- Acute paralysis/flaccid paralysis[2]	- General physical exam performed by physician/nurse (calm, fussy, interactive; looks sick or well)<br>- Liver palpation<br>- Heart rate<br>- Exam for oedema (puffy eyelids, pitting oedema of back, genitals or ankles, whole body puffy)<br>- Respiratory distress (retractions, nasal flaring, paradoxical breathing, head bobbing)<br>- Respiratory rate, oxygen saturation, perfusion index<br>- Temperature<br>- Documentation of abnormal eye movements and eye contact<br>- Examine for signs of ptosis<br>- Hypotonia (head lag, floppy arms or legs in face-down position)<br>- Consciousness evaluation (AVPU scale)<br>- Documentation of timing of onset of each symptom<br>- Anthropometric indices (length, weight, mid-upper arm circumference, head circumference)<br>- Ultrasounds:<br>- Echocardiogram<br>- Cranial ultrasound<br><br>Follow up after thiamine administration:<br>- Repetition of physical exam after 4, 8 and 12 hours and every 12 hours for first 48 hours<br>- After 24 and 48 hrs: Echocardiogram<br>- After 72 hrs: Repetition of physical exam	- ETKac in washed RBC<br>- Whole blood ThDP<br>- CBC<br>- Troponin T<br>- Lactate<br>- Indicators of inflammation (CRP, AGP)	- Basic demographics of child (age, sex, location of residence)<br>- Socioeconomic status of mother and family<br>- Gestational age at birth<br>- Birth/perinatal history<br>- Birth weight and length<br>- History of severe illnesses since birth and hospitalisations<br>- History of any illnesses in 2 weeks prior to onset of current illness<br>- History of bowel movements in past 24 hours (number and consistency), urinary outputs in past 24 hours and constipation in previous 7 days<br>- Recent and current intake of medications, dietary supplements and use of Tiger Balm<br>- Documentation of treatments and medications prescribed due to current illness, and thiamine at discharge<br>- Vaccination record<br>- Dietary practices of child (breastfeeding, complementary feeding)<br>- Maternal report on recent changes in physical movements, motor skills and behaviour

For peer review only

For peer review only - http://bmjopen.bmj.com/site/about/guidelines.xhtml

	For DSMB:<br>- Response to thiamine injection/administration (such as local rash/redness, infection/swelling, temporary lethargy)		
Mothers of hospitalised children			
Exclude if:<br>- Severe acute illness warranting immediate hospital referral<br>- Unable to provide informed consent due to reduced decision making ability	- Maternal height, weight and mid-upper arm circumference	- ETKac in washed RBC<br>- Whole blood ThDP<br>- Indicators of inflammation (CRP, AGP)<br>- CBC<br>- Dried blood spots<br>- Breast milk thiamine concentration of lactating mothers	- Maternal characteristics (age, education, profession, marital status, ethnicity, religion, location of residence, family structure, number of children, etc.)<br>- Recent and current intake of multi-micronutrient supplements, medications and use of Tiger Balm<br>- Thiamine given at discharge<br>- Dietary practices of mother (dietary diversity questionnaire, food preparation, dietary beliefs/avoidance of certain foods, consumption of raw fish/fish paste, betel nut, tea and alcohol)<br>- Food security questionnaire<br>- History of beriberi (maternal beriberi and mother's children)<br>- Social desirability bias
Community-based age- and sex-matched children			
- Excluded if immediate hospital referral is required<br>- No other exclusion criteria	- General physical exam performed by study nurse<br>- Heart rate<br>- Respiratory rate, oxygen saturation, perfusion index<br>- Temperature<br>- Hypotonia (head lag, floppy arms or legs in face-down position)<br>- Anthropometric indices (length, weight, mid-upper arm circumference, head circumference)	- ETKac in washed RBC<br>- Whole blood ThDP<br>- CBC<br>- Indicators of inflammation (CRP, AGP)	- Basic demographics of child (age, sex, location of residence)<br>- Socioeconomic status of mother and family<br>- Gestational age at birth<br>- Birth/perinatal history<br>- Birth weight and length<br>- History of severe illnesses since birth and hospitalisations<br>- History of any illnesses in past 2 weeks<br>- History of bowel movements in past 24 hours (number and consistency), urinary outputs in past 24 hours and constipation in previous 7 days<br>- Recent and current intake of medications, dietary supplements and use of Tiger Balm<br>- Vaccination record<br>- Dietary practices of child (breastfeeding, complementary feeding)

For peer review only

			- Maternal report on recent changes in physical movements, motor skills and behaviour
Mothers of children in the community			
Exclude if:<br>- Severe acute illness warranting immediate hospital referral<br>- Unable to provide informed consent due to reduced decision making ability	- Maternal height and weight and mid-upper arm circumference	- ETKac in washed RBC<br>- Whole blood ThDP<br>- Indicators of inflammation (CRP, AGP)<br>- CBC<br>- Dried blood spots<br>- Breast milk thiamine concentration of lactating mothers	- Maternal characteristics (age, education, profession, marital status, ethnicity, religion, location of residence, family structure, number of children, etc.)<br>- Recent and current intake of multi-micronutrient supplements, medications and Tiger Balm<br>- Dietary practices of mother (dietary diversity questionnaire, food preparation, dietary beliefs/avoidance of certain foods, consumption of raw fish/fish paste, betel nut, tea and alcohol)<br>- Food security questionnaire<br>- History of beriberi (maternal beriberi and mother's children)<br>- Social desirability bias

AGP, α-1-acid glycoprotein; CBC, complete blood count; CRP, C-reactive protein; DSMB, data safety monitoring board; ETKac, erythrocyte transketolase activity coefficient; RBC, red blood cells; ThDP, thiamine diphosphate

[1] At the onset of the study, the eligibility age was 21 days to <12 months. The target age was expanded to <18 months after 18.1% hospital infants were enroled due to much lower enrolment rates than anticipated, and after consultation with expert paediatricians and the hospital leadership.
[2] Tachycardia, opisthotonus/abnormal posturing and acute paralysis/flaccid paralysis were added as inclusion criteria at the time when the target age range was extended to <18 months of age.

For peer review only

SPIRIT
STANDARD PROTOCOL ITEMS: RECOMMENDATIONS FOR INTERVENTIONAL TRIALS

SPIRIT 2013 Checklist: Recommended items to address in a clinical trial protocol and related documents*

Section/item	ItemNo	Description	Page
**Administrative information**			
Title	1	Descriptive title identifying the study design, population, interventions, and, if applicable, trial acronym	1
Trial registration	2a	Trial identifier and registry name. If not yet registered, name of intended registry	20
	2b	All items from the World Health Organization Trial Registration Data Set	n/a
Protocol version	3	Date and version identifier	22
Funding	4	Sources and types of financial, material, and other support	23
Roles and responsibilities	5a	Names, affiliations, and roles of protocol contributors	22
	5b	Name and contact information for the trial sponsor	1
	5c	Role of study sponsor and funders, if any, in study design; collection, management, analysis, and interpretation of data; writing of the report; and the decision to submit the report for publication, including whether they will have ultimate authority over any of these activities	23
	5d	Composition, roles, and responsibilities of the coordinating centre, steering committee, endpoint adjudication committee, data management team, and other individuals or groups overseeing the trial, if applicable (see Item 21a for data monitoring committee)	n/a
**Introduction**			
Background and rationale	6a	Description of research question and justification for undertaking the trial, including summary of relevant studies (published and unpublished) examining benefits and harms for each intervention	6-8

For peer review only

	6b	Explanation for choice of comparators	5
Objectives	7	Specific objectives or hypotheses	8
Trial design	8	Description of trial design including type of trial (eg, parallel group, crossover, factorial, single group), allocation ratio, and framework (eg, superiority, equivalence, noninferiority, exploratory)	8
**Methods: Participants, interventions, and outcomes**			
Study setting	9	Description of study settings (eg, community clinic, academic hospital) and list of countries where data will be collected. Reference to where list of study sites can be obtained	9
Eligibility criteria	10	Inclusion and exclusion criteria for participants. If applicable, eligibility criteria for study centres and individuals who will perform the interventions (eg, surgeons, psychotherapists)	9-11
Interventions	11a	Interventions for each group with sufficient detail to allow replication, including how and when they will be administered	12
	11b	Criteria for discontinuing or modifying allocated interventions for a given trial participant (eg, drug dose change in response to harms, participant request, or improving/worsening disease)	n/a
	11c	Strategies to improve adherence to intervention protocols, and any procedures for monitoring adherence (eg, drug tablet return, laboratory tests)	n/a
	11d	Relevant concomitant care and interventions that are permitted or prohibited during the trial	13
Outcomes	12	Primary, secondary, and other outcomes, including the specific measurement variable (eg, systolic blood pressure), analysis metric (eg, change from baseline, final value, time to event), method of aggregation (eg, median, proportion), and time point for each outcome. Explanation of the clinical relevance of chosen efficacy and harm outcomes is strongly recommended	21-22
Participant timeline	13	Time schedule of enrolment, interventions (including any run-ins and washouts), assessments, and visits for participants. A schematic diagram is highly recommended (see Figure)	14 Figure 2

For peer review only - http://bmjopen.bmj.com/site/about/guidelines.xhtml

Sample size	14	Estimated number of participants needed to achieve study objectives and how it was determined, including clinical and statistical assumptions supporting any sample size calculations	20
Recruitment	15	Strategies for achieving adequate participant enrolment to reach target sample size	9-10

Methods: Assignment of interventions (for controlled trials)

Allocation:			
Sequence generation	16a	Method of generating the allocation sequence (eg, computer-generated random numbers), and list of any factors for stratification. To reduce predictability of a random sequence, details of any planned restriction (eg, blocking) should be provided in a separate document that is unavailable to those who enrol participants or assign interventions	n/a
Allocation concealment mechanism	16b	Mechanism of implementing the allocation sequence (eg, central telephone; sequentially numbered, opaque, sealed envelopes), describing any steps to conceal the sequence until interventions are assigned	n/a
Implementation	16c	Who will generate the allocation sequence, who will enrol participants, and who will assign participants to interventions	n/a
Blinding (masking)	17a	Who will be blinded after assignment to interventions (eg, trial participants, care providers, outcome assessors, data analysts), and how	n/a
	17b	If blinded, circumstances under which unblinding is permissible, and procedure for revealing a participant's allocated intervention during the trial	n/a

Methods: Data collection, management, and analysis

Data collection methods	18a	Plans for assessment and collection of outcome, baseline, and other trial data, including any related processes to promote data quality (eg, duplicate measurements, training of assessors) and a description of study instruments (eg, questionnaires, laboratory tests) along with their reliability and validity, if known. Reference to where data collection forms can be found, if not in the protocol	18

	18b	Plans to promote participant retention and complete follow-up, including list of any outcome data to be collected for participants who discontinue or deviate from intervention protocols	14
Data management	19	Plans for data entry, coding, security, and storage, including any related processes to promote data quality (eg, double data entry; range checks for data values). Reference to where details of data management procedures can be found, if not in the protocol	20
Statistical methods	20a	Statistical methods for analysing primary and secondary outcomes. Reference to where other details of the statistical analysis plan can be found, if not in the protocol	21-22
	20b	Methods for any additional analyses (eg, subgroup and adjusted analyses)	22
	20c	Definition of analysis population relating to protocol non-adherence (eg, as randomised analysis), and any statistical methods to handle missing data (eg, multiple imputation)	21-22
**Methods: Monitoring**			
Data monitoring	21a	Composition of data monitoring committee (DMC); summary of its role and reporting structure; statement of whether it is independent from the sponsor and competing interests; and reference to where further details about its charter can be found, if not in the protocol. Alternatively, an explanation of why a DMC is not needed	23
	21b	Description of any interim analyses and stopping guidelines, including who will have access to these interim results and make the final decision to terminate the trial	23
Harms	22	Plans for collecting, assessing, reporting, and managing solicited and spontaneously reported adverse events and other unintended effects of trial interventions or trial conduct	22
Auditing	23	Frequency and procedures for auditing trial conduct, if any, and whether the process will be independent from investigators and the sponsor	n/a
**Ethics and dissemination**			

For peer review only - http://bmjopen.bmj.com/site/about/guidelines.xhtml

Research ethics approval	24	Plans for seeking research ethics committee/institutional review board (REC/IRB) approval	22
Protocol amendments	25	Plans for communicating important protocol modifications (eg, changes to eligibility criteria, outcomes, analyses) to relevant parties (eg, investigators, REC/IRBs, trial participants, trial registries, journals, regulators)	22
Consent or assent	26a	Who will obtain informed consent or assent from potential trial participants or authorised surrogates, and how (see Item 32)	11-12
	26b	Additional consent provisions for collection and use of participant data and biological specimens in ancillary studies, if applicable	10
Confidentiality	27	How personal information about potential and enrolled participants will be collected, shared, and maintained in order to protect confidentiality before, during, and after the trial	
Declaration of interests	28	Financial and other competing interests for principal investigators for the overall trial and each study site	23
Access to data	29	Statement of who will have access to the final trial dataset, and disclosure of contractual agreements that limit such access for investigators	18
Ancillary and post-trial care	30	Provisions, if any, for ancillary and post-trial care, and for compensation to those who suffer harm from trial participation	n/a
Dissemination policy	31a	Plans for investigators and sponsor to communicate trial results to participants, healthcare professionals, the public, and other relevant groups (eg, via publication, reporting in results databases, or other data sharing arrangements), including any publication restrictions	21
	31b	Authorship eligibility guidelines and any intended use of professional writers	21
	31c	Plans, if any, for granting public access to the full protocol, participant-level dataset, and statistical code	23

Appendices

For peer review only - http://bmjopen.bmj.com/site/about/guidelines.xhtml

Informed consent materials	32	Model consent form and other related documentation given to participants and authorised surrogates	osf
Biological specimens	33	Plans for collection, laboratory evaluation, and storage of biological specimens for genetic or molecular analysis in the current trial and for future use in ancillary studies, if applicable	n/a

*It is strongly recommended that this checklist be read in conjunction with the SPIRIT 2013 Explanation & Elaboration for important clarification on the items. Amendments to the protocol should be tracked and dated. The SPIRIT checklist is copyrighted by the SPIRIT Group under the Creative Commons "Attribution-NonCommercial-NoDerivs 3.0 Unported" license.

For peer review only

For peer review only - http://bmjopen.bmj.com/site/about/guidelines.xhtml
