# Supplementary material for: Establishing a case definition of thiamine responsive disorders among infants and young children in Lao PDR: protocol for a prospective cohort study
Source: BMJ Open. 2020 Feb 13;10(2):e036539. doi: 10.1136/bmjopen-2019-036539 (PMC7044841; doi:10.1136/bmjopen-2019-036539)
Supplement: Reviewer comments [file bmjopen-2019-036539.reviewer_comments.pdf]

PEER REVIEW HISTORY

BMJ Open publishes all reviews undertaken for accepted manuscripts. Reviewers are asked to complete a checklist review form (**http://bmjopen.bmj.com/site/about/resources/checklist.pdf**) and are provided with free text boxes to elaborate on their assessment. These free text comments are reproduced below.

ARTICLE DETAILS

**TITLE (PROVISIONAL)**	Establishing a case definition of thiamine responsive disorders among infants and young children in Lao PDR: Protocol for a prospective cohort study
**AUTHORS**	Hess, Sonja; Smith, Taryn; Fischer, Philip; Trehan, Indi; Hiffler, Laurent; Arnold, Charles D; Sitthideth, Dalaphone; Tancredi, Daniel; Schick, Michael; Yeh, Jay; Stein-Wexler, Rebecca; McBeth, Christine; Tan, Xiuping; Nhiacha, Kouyang; Kounnavong, Sengchanh

This article was not externally reviewed at BMJ Open. Protocols that have been independently assessed prior to submission to BMJ Open are usually fast-tracked to publication on the grounds that further substantial changes will not be possible. This independent assessment will usually be external, independent review for both a substantial grant award from a non-commercial or government funder and ethics approval.
